# Natural Product-Derived Ianthelliformisamines Inhibit Protein Translation and Block Bacterial Flagellum Assembly

**DOI:** 10.1021/acschembio.5c01018

**Published:** 2026-04-21

**Authors:** Max Bottlinger, Martino Morici, Elena Fajardo-Ruiz, Isabella Gantner, Sophie Brameyer, Michael Isselstein, Max Berger, Daniel N. Wilson, Kirsten Jung, Stephan A. Sieber

**Affiliations:** † Center for Functional Protein Assemblies, Department of Bioscience, TUM School of Natural Sciences, Technische Universität München, Ernst-Otto-Fischer-Straße 8, 85748 Garching, Germany; ‡ Institute for Biochemistry and Molecular Biology, 14915 University of Hamburg, 20146 Hamburg, Germany; § Faculty of Biology, Microbiology, Ludwig-Maximilians-Universität München, 82152 Martinsried, Germany; ∥ Faculty of Biology, Plant Development and Electron Microscopy, Ludwig-Maximilians-Universität München, 82152 Martinsried, Germany; ⊥ Faculty of Biology, Ludwig-Maximilians-Universität München, 82152 Martinsried, Germany; # Center for Geometrically Engineered Cellular Membranes, Department of Chemistry, University of Copenhagen, Copenhagen DK-2100, Denmark

## Abstract

Ianthelliformisamines (Ian) represent a poorly characterized natural product class reported to inhibit Gram-negative bacteria such as *Escherichia coli*. Given the current antibiotic crisis, revisiting poorly characterized antibacterial natural products may reveal novel modes of action (MoA) as inspiration for drug development. Thus, we elucidated the Ian mode of action and synthesized three Ian analogs along with a chemical probe for activity-based protein profiling (ABPP). All molecules retained antibacterial effects, which were enhanced in the presence of bicarbonate, an abundant ingredient of human serum. Chemical proteomics with the probe unraveled InfA, involved in the initiation of bacterial ribosomal protein biosynthesis, as an essential target, which was confirmed by translation assays. Intriguingly, a virulence-associated target stood out as an additional hit, FliC, with a crucial role in flagellum assembly. The recombinant protein was probe-labeled, and motility assays together with transmission electron microscopy revealed impaired motility and disrupted flagellum assembly, respectively. Consistent with this dual antibacterial/antivirulence profile, Ian treatment reduced invasion of pathogenic *E. coli* into human host cells. This work illustrates how activity-based chemical proteomics can uncover previously unrecognized cellular targets for natural product scaffolds, thereby revealing distinct modes of action and supporting further therapeutic development.

## Graphical abstract

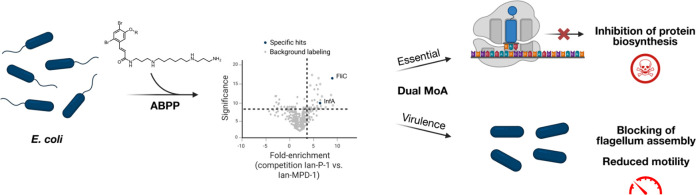

## Introduction

Many currently prescribed antibiotics address essential cellular functions such as cell wall synthesis, transcription, or translation.^1^ For example, the inhibition of translation is a hot spot mechanism for many drugs, such as tetracyclines or macrolides.^2,3^ In addition, associated proteins such as initiation factors have been identified as powerful targets to inhibit the start of protein biosynthesis by preventing the assembly of the initiation complex.^4,5^ Despite the continuous development of novel and innovative protein biosynthesis inhibitors, several drugs on the market suffer from resistance due to mutations in conserved ribosomal binding sites.^6,7^ Addressing the challenge of resistance requires not only new translation-directed chemotypes and underexploited targets, but also complementary modes of action that may reduce the selective pressure associated with bactericidal treatments. One emerging approach is the development of antivirulence agents (“pathoblockers”) that disarm pathogenic traits such as toxin production, biofilm formation, or motility (e.g., flagellum-driven swimming), thereby limiting infection without necessarily killing the pathogen.^8–12^ Natural products remain a rich source of chemically diverse scaffolds and bioactivities, but for many candidates, the relevant cellular targets and modes of action are still unknown.^13–15^ This knowledge is of high relevance, given that even nondruglike natural products can inspire target discovery and subsequent medicinal chemistry.^16–18^

In the search for novel MoAs, we and others utilize chemical proteomics to decipher the cellular targets of less-exploited scaffolds directly in bacterial cells.^19–21^ Of particular interest are compounds bearing electrophilic moieties, which often covalently react with nucleophilic sites of target proteins. This is advantageous in bacteria as covalent, irreversible binding enhances the duration of the biological effect and reduces the susceptibility to efflux.^22,23^ In the search for antibacterial natural products bearing electrophilic moieties and lacking functional characterization, we came across Ianthelliformisamine A–C (**Ian-A–C**), brominated aryl-polyamines with a benzylic Michael acceptor moiety (Figure 1a). Ianthelliformisamine A–C were isolated from an Australian marine sponge (*Suberea ianthelliformis*) in 2012, and exhibited moderate activity against *Pseudomonas aeruginosa* (17–35 μM).^24^ Recently, additional members of this compound class were discovered (Ianthelliformisamine D–G), but they lacked antibiotic activity.^25^ Interestingly, Ianthelliformisamine A and B synergized with ciprofloxacin and inhibited biofilm formation of *P. aeruginosa*, while direct effects on the bacterial membrane could be excluded.^26,27^ Of note, structure–activity relationship (SAR) studies revealed a derivative **Ian-MPD-1** with extensions in the polyamine alkyl chain and slightly different substitution pattern at the aromatic system which significantly enhanced antibiotic potency against *Escherichia coli* and *Staphylococcus aureus* (Figure 1a).^28^

**Figure 1:**
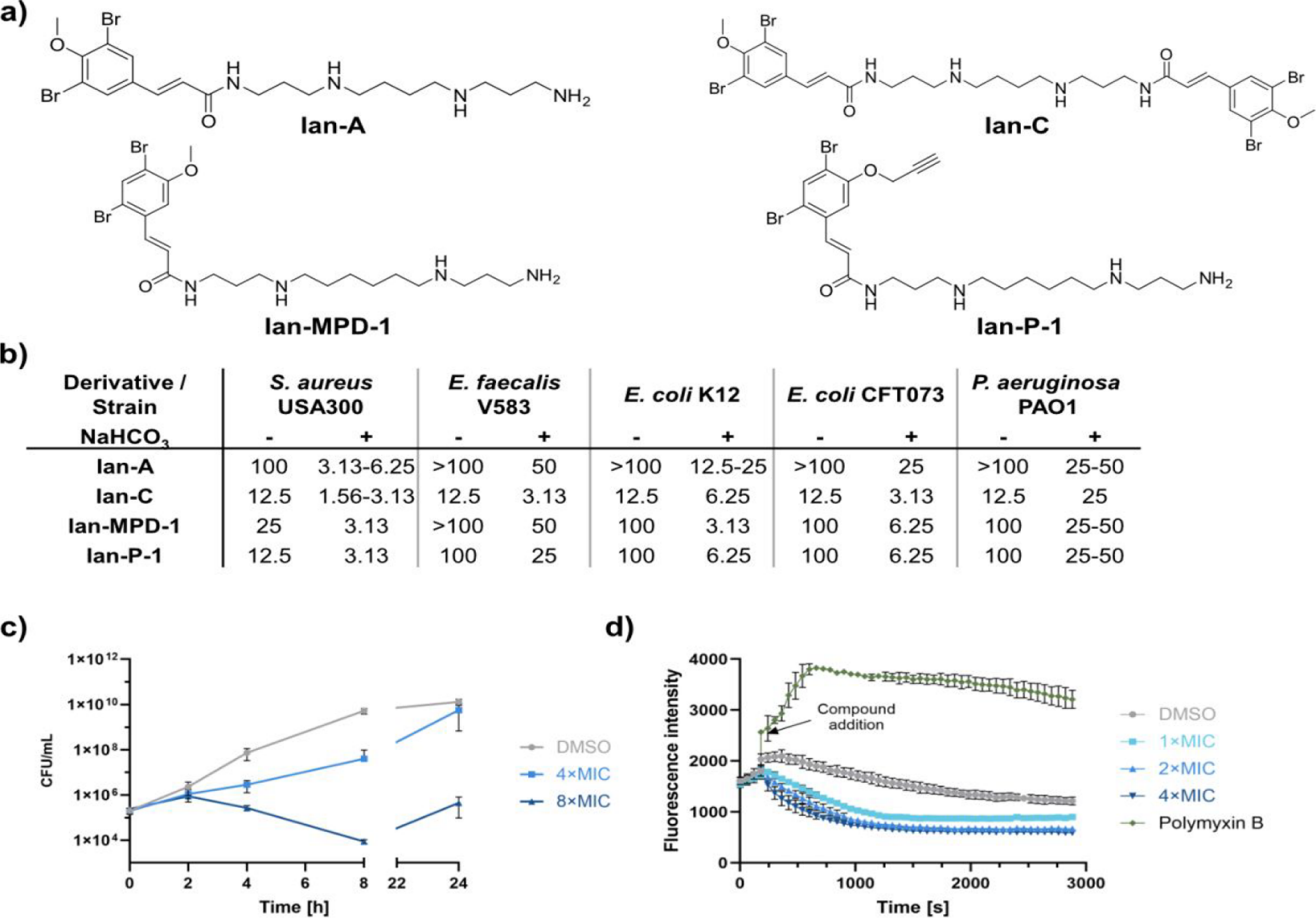
Synthesis and antibiotic activity of Ianthelliformisamines. (a) Structure of **Ian-A, Ian-C,** the most potent derivative **Ian-MPD-1**, and the respective ABPP-probe **Ian-P-1**. (b) Minimal inhibitory concentration [μM] against different Gram-positive and Gram-negative bacteria in the presence of and without sodium bicarbonate (25 mM). Results are the means of technical replicates and are confirmed in independent measurements. Ranges are given for variations between independent measurements. (c) Time-kill assay in *E. coli* K12 with **Ian-MPD-1** in the presence of sodium bicarbonate (25 mM). After 0, 2, 4, 8, and 24 h, viable cells (CFU/mL) were determined in quadruplicates. Data represent the mean values + SD of *n* = 2 independent experiments. (d) Membrane potential assay in *E. coli* K12. Cells are treated with **Ian-MPD-1** and resulting fluorescence intensity of membrane potential-sensitive dye 3,3′-dipropylthiadicarbocyanine iodide (DiSC_3_(5)) is measured. DMSO and Polymyxin B are used as negative and positive controls, respectively. Data represent the mean + SD of three technical replicates and are representative of at least two independent biological replicates.

Given these interesting biological properties along with the distinct aryl Michael acceptor, we synthesized Ianthelliformisamine natural products and derivatives which exhibited potent antibacterial activity in the presence of sodium bicarbonate, a highly abundant ingredient of human serum. Activity-based protein profiling (ABPP) revealed initiation factor InfA as the sole essential target and the flagellum-associated protein FliC as a motility and thus pathogenesis-associated target. In-depth validation via ribosome assays, mutational studies, proteomics, electron microscopy, and swimming assays confirmed the functional impact of Ianthelliformisamine binding to FliC and InfA. Importantly, this dual MoA resulted in reduced human cell invasion of compound-treated bacteria. Given the therapeutic window, Ianthelliformisamines represent promising candidates for further antibacterial development.

## Results and Discussion

### Synthesis and Antibiotic Activity of Ianthelliformisamines

Prior to MoA studies, we synthesized a representative set of Ianthelliformisamine (Ian) natural products, i.e., **Ian-A** and **Ian-C**, a dimeric derivative of **Ian-A**. Given the reported enhanced antibiotic activity of an **Ian-A** derivative with an extended alkyl chain and a slightly different substitution pattern on the phenyl ring, we also prepared this compound, termed **Ian-MPD-1** (Scheme 1). The syntheses followed published procedures initiated by Khan et al.^28^ The synthetic route involves the late-stage peptide coupling of a carboxylic acid fragment (CA) with a diamine fragment (DA) (Scheme 1). The CA fragment was prepared over four steps, featuring a key olefination reaction, and was obtained in an overall yield of 18–58%. The DA fragment was synthesized in three steps with an overall yield of 36–49%, involving an initial aza-Michael addition, followed by a protection step and a final reduction.

**Scheme 1:**
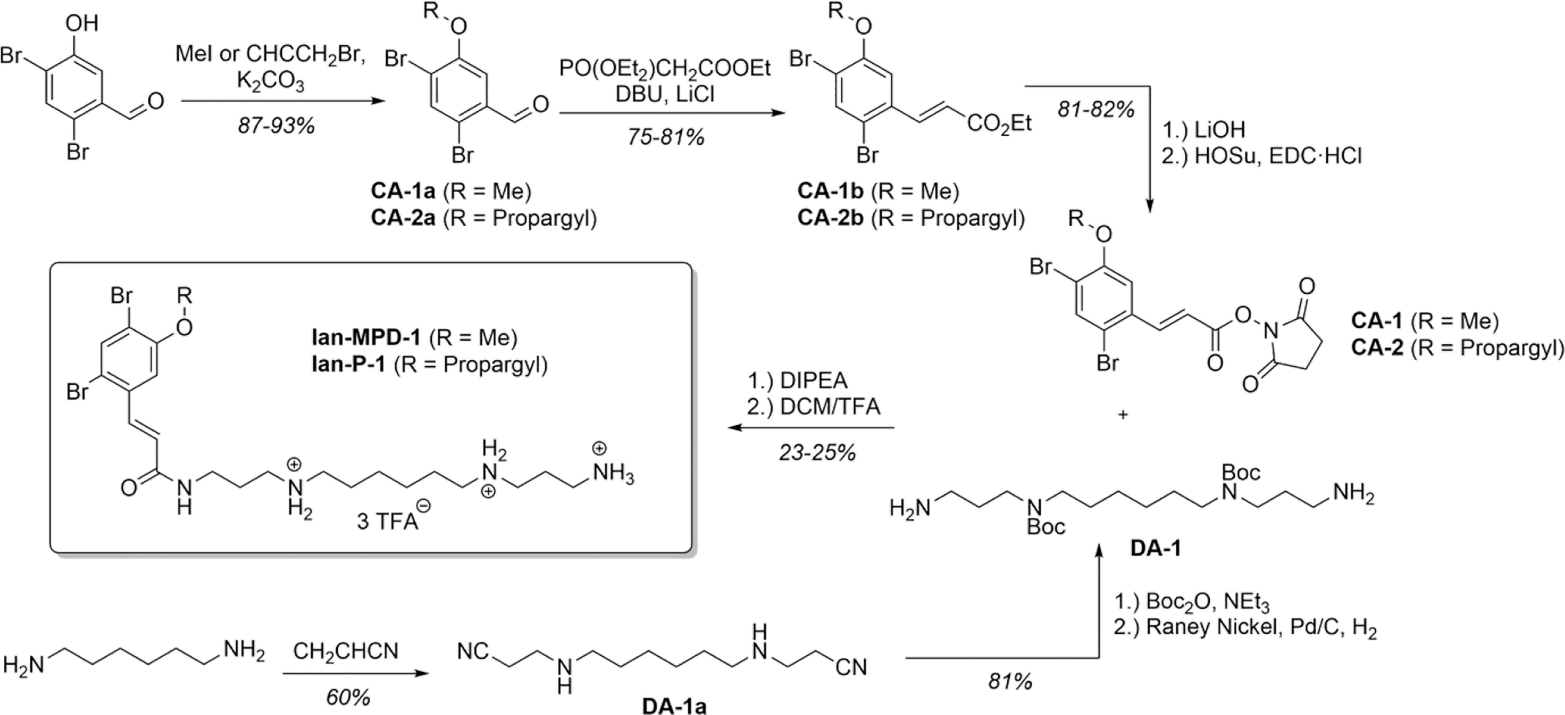
Synthesis Scheme for the Synthesis of **Ian-MPD-1** or **Ian-P-1** ^a^

a The carboxylic acid (CA) fragment is synthesized via the alkylation of 2,4-dibromo-5-hydroxybenzaldehyde at the phenolic position, followed by Horner-Wadsworth-Emmons reaction and NHS-active ester formation. The Diamine fragment (DA) synthesis starts with a Michael addition of hexane-1,6-diamine and acrylonitrile, followed by protection and reduction. The two fragments **CA-1**/**CA-2** and **DA-1** are combined by peptide coupling and deprotection, yielding either **Ian-MPD-1** or **Ian-P-1**. The synthesis was previously described and adapted with slight modifications.^28^ Me = Methyl, Et = Ethyl, DBU = 1,8-Diazabicyclo(5.4.0)­undec-7-ene, HOSu = *N*-Hydroxysuccinimide, EDC·HCl = 1-Ethyl-3-(3-dimethylaminopropyl)­carbodiimide Hydrochloride, Boc_2_O = Di-*tert*-Butylpyrocarbonat, DIPEA = *N*-Ethyldiisopropylamin, DCM = Dichloromethane, TFA = Trifluoroacetic acid.

With different derivatives of Ianthelliformisamines in hand, we tested their activity against a panel of Gram-negative and Gram-positive bacteria (Figure 1b). Moderate activity against Gram-positive cells such as *S. aureus* with minimal inhibitory concentrations (MIC) ranging from 12.5 to 25 μM for **Ian-C** and **Ian-MPD-1** and 100 μM for **Ian-A** were observed. Similarly, Gram-negative bacteria such as *P. aeruginosa* or *E. coli* bearing an impermeable double cell membrane exhibited growth reduction in the presence of 100 μM **Ian-A** and 12.5 μM **Ian-C**, respectively, which is in line with previous reports.^24,27^ Furthermore, testing against *E. coli* variants, either deficient in efflux or LPS biosynthesis, did not enhance antibiotic activity (Table S1).

As bicarbonate effects have been previously observed, we examined the possibility of enhanced activity in the presence of bicarbonate, a component of human plasma (physiological concentration: 25 mM) known to stimulate the activity of certain antibiotics.^29,30^ Bicarbonate has been shown to modulate the bacterial proton motive force by perturbing the proton gradient of the membrane, which is compensated by an increase in the electrical potential. Farha et al. showed that the uptake of cationic antibiotics is mainly driven by the electrical potential, leading to an increased uptake with increasing cationic charge. In addition, the study’s results indicate that bicarbonate compromises the energetic basis of efflux. In fact, in the presence of bicarbonate the MIC values of all compounds significantly dropped, and ranged, e.g., for *E. coli* between 3 and 6 μM for **Ian-C** as well as **Ian-MPD-1** and 12–25 μM for **Ian-A**. Time-kill assays in the presence of bicarbonate demonstrated a bactericidal MoA which was independent of the bacterial membrane integrity, as shown by negative depolarization and permeabilization assays (Figures 1c,d, S1, and S2). Moreover, DNA was excluded as a binding partner by MIC shift assays (Figure S3). Overall, our results show that Ianthelliformisamines are antibacterially active but require adjuvants such as bicarbonate in order to exhibit pronounced antibacterial effects, which do not involve the membrane or DNA binding.

### Chemical Proteomics with a Ianthelliformisamine Probe Unravels Translation as Well as Motility-Associated Targets

Prior to target identification, we monitored changes in the expression of *E. coli* proteins upon incubation with **Ian-MPD-1** via mass-spectrometric (MS) full proteome analysis. To maximize antibacterial-associated changes, the cells were suspended in buffer containing 25 mM bicarbonate followed by treatment with the compound. LC–MS/MS analysis via data-independent acquisition (DIA) and label-free quantification (LFQ) of treated vs untreated cells and subsequent pathway analysis revealed a significant up-regulation of proteins associated with the flagellum or cell motility, respectively (Figure S4, Table S2).

Based on these initial insights, we designed an ABPP probe for the direct binding to protein targets inside living cells. Given the structure of **Ian-MPD-1**, we selected the methoxy group of the aromatic ring system as a suitable position for propargylation, ensuring minimal perturbation of the overall structural composition. We deliberately did not incorporate a photo-cross-linking unit to decipher if the Michael acceptor would facilitate covalent binding and thus protein labeling. The synthesis followed our established procedure, starting with 2,4-dibromo-5-hydroxybenzaldehyde, yielding probe **Ian-P-1** (Scheme 1). Satisfyingly, **Ian-P-1** exhibited the same spectrum of activity as **Ian-MPD-1,** highlighting its utility for target identification studies (Figure 1b). These were performed by incubating live *E. coli* cells with various concentrations of **Ian-P-1** for 1 h in the presence of sodium bicarbonate, followed by cell lysis and click chemistry to rhodamine azide for fluorescent analysis of labeled proteins via SDS-PAGE. The corresponding gels revealed a clear concentration-dependent labeling of distinct protein bands with an optimal concentration for saturated labeling of 6 μM (Figure S5a). Comparison of labeling efficiency between in situ treated *E. coli* cells and lysate labeling in the absence of bicarbonate showed target engagement in intact cells only in the presence of bicarbonate, whereas lysate labeling was sufficient in its absence (Figure S5b,c). Target identification commenced by click chemistry of in situ treated *E. coli* proteomes to biotin azide, avidin enrichment, tryptic digest, and LC–MS/MS analysis via LFQ and DIA (Figure 2a). Proteins that were significantly enriched compared to a DMSO control are depicted in a volcano plot (significance criteria log_2_ fold change = 1, *P*-value = 0.05, Figure 2a). In addition, we cotreated cells by probe addition and a 4-fold excess of **Ian-MPD-1** to narrow down targets that are not only probe-enriched but also bind to the parent compound. Overall, we identified six protein hits that fulfilled our selection criteria in the enrichment experiment (Figure 2b, Table S3). Out of those six enriched proteins, only two proteins were competed by a 4-fold excess of the parent compound (Figure 2c). Given the lack of any photo-cross-linking unit, the enrichment of these proteins indeed confirms a covalent binding mechanism of the compound. A closer inspection of the proteins that are enriched and outcompeted, FliC and InfA, revealed their roles in flagellum motility and protein synthesis, respectively. Of note, among these, InfA, an initiation translation factor crucial for ribosomal protein synthesis, is the only essential protein that could be attributed to the observed antibacterial effect of Ianthelliformisamines.

**Figure 2:**
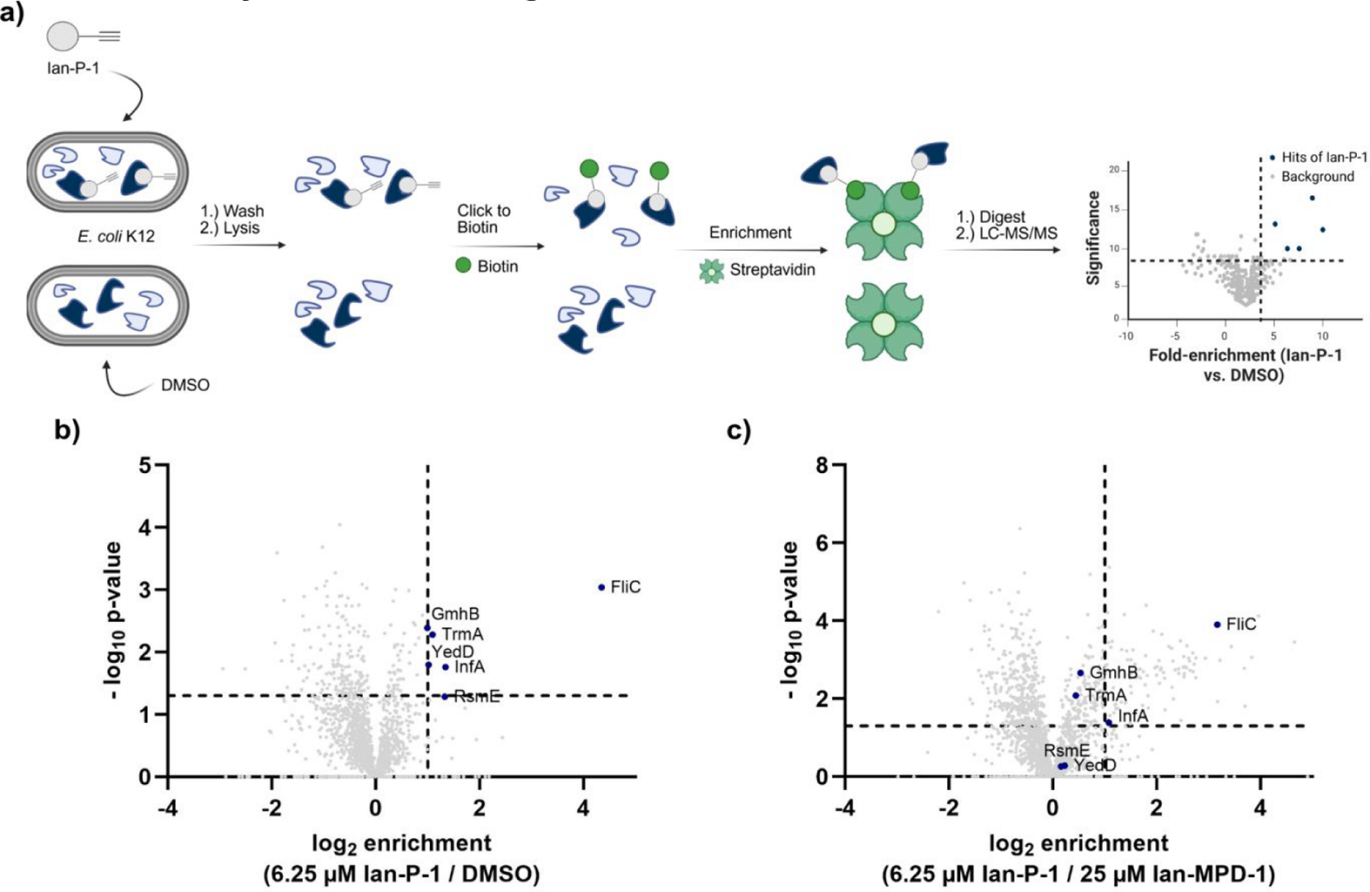
ABPP to identify protein targets. (a) Schematic overview of the ABPP workflow for protein target identification used in this study. *E. coli* K12 cultures are treated with **Ian-P-1** or vehicle control (DMSO), washed, and lysed. After clicking to biotin-azide, the probe-bound proteins are enriched on streptavidin beads. Proteins are digested by trypsin and submitted to LC–MS/MS analysis for protein identification. (b) Volcano plot of *E. coli* K12 cells treated with 6.25 μM **Ian-P-1** (alkynylated probe) compared to DMSO (1%). Dotted lines indicate significance cutoff *P*-value ≤ 0.05 (*n* = 4) and log_2_ fold change ≥ 1 and proteins meeting both criteria are highlighted in blue. (c) Volcano plot of *E. coli* K12 cells treated with 6.25 μM **Ian-P-1** (alkynylated probe) compared to 6.25 μM **Ian-P-1** in competition with 25 μM **Ian-MPD-1** (parent compound). Proteins enriched in (b) and competed in (c) are shown by name (InfA and FliC). All other proteins were not enriched in (b). Dotted lines indicate significance cutoff *P*-value ≤ 0.05 (*n* = 4) and log_2_ fold change ≥ 1. Proteins significantly enriched in (b) are highlighted in blue.

### Ianthelliformisamines Exhibit a Dual Mode of Action by Inhibiting Bacterial Translation and Motility

We first focused our follow-up studies on the essential translation initiation factor InfA and performed an *E. coli* cell-free lysate-based in vitro translation assay. **Ian-MPD-1** showed a potent IC_50_ of 5 μM, confirming a pronounced effect on protein biosynthesis (Figure 3a). In line with their weaker potency, **Ian-A** and **Ian-C** exhibited IC_50_ values of 39 μM and 30 μM, respectively, in this assay. Of note, the presence of bicarbonate in these assays had only a slight impact on the activity, suggesting that the adjuvant has no direct effect on the compound or target (Figure S7).

**Figure 3:**
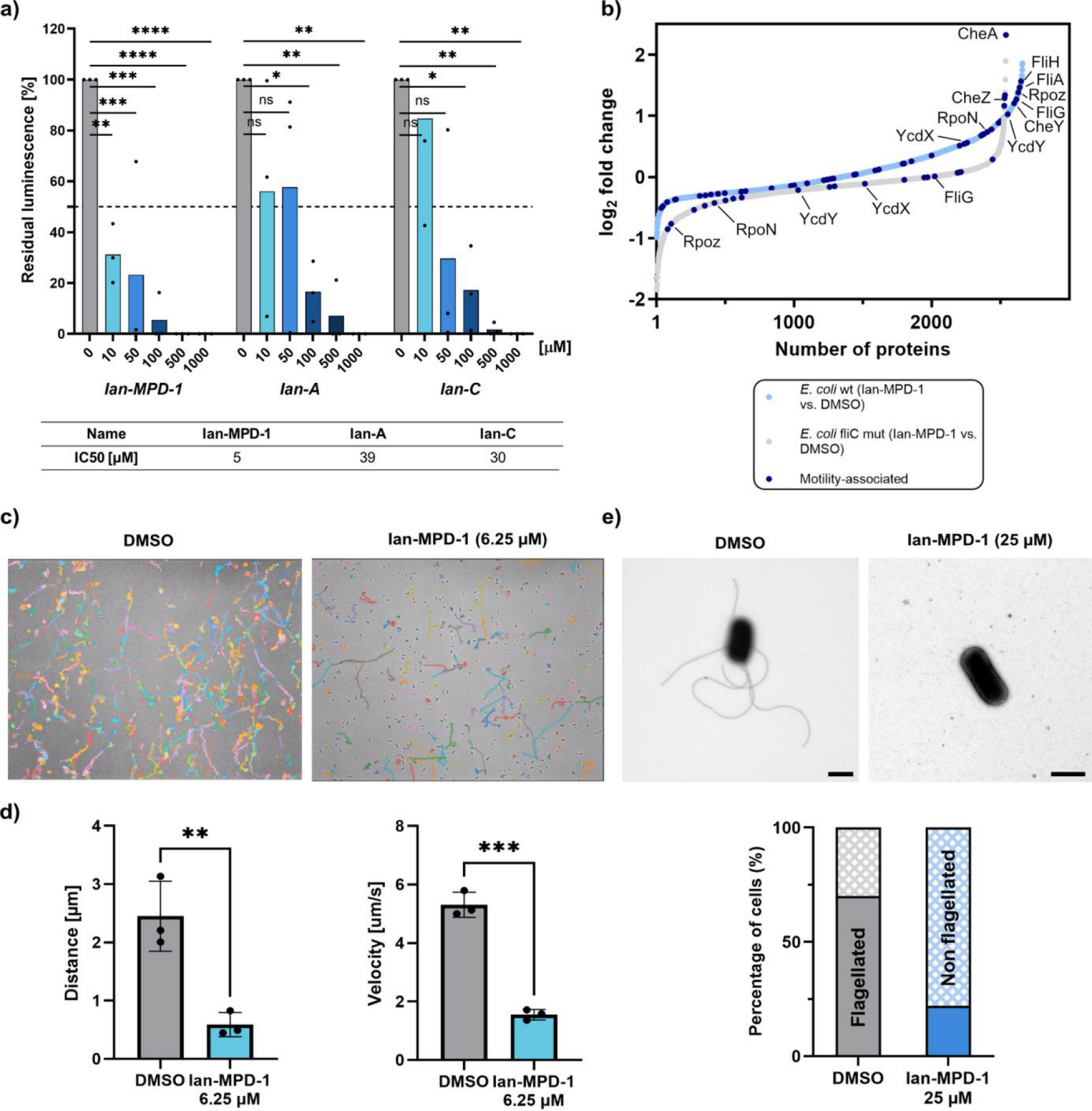
**Ian-MPD-1** is targeting protein biosynthesis, flagella motility, and assembly. (a) Concentration-dependent in vitro assay in the absence and presence of **Ian-MPD-1**, **Ian-A**, and **Ian-C** with an *E. coli* cell-free lysate-based translation system expressing a firefly luciferase (Fluc) reporter. IC_50_ values were calculated using GraphPad Prism by fitting a sigmoidal dose–response curve (variable slope). Experiments were performed in three independent replicates. Statistical relevance is shown based on a paired one-way ANOVA test (*P-value <0.02, **P-value < 0.01, ***P-value <0.001, ****P-value <0.0001, ns = nonsignificant). Assay quality was assessed by *Z*′ (see Table S4). (b) Waterfall plot of proteins detected in a full proteome analysis of treated (6.25 μM **Ian-MPD-1**) vs untreated *E. coli* K12 wt or *E. coli* fliC mutant (Keio collection, JW1908),^31^ respectively. Proteins highlighted in blue: Cell motility (GO-term: “cell motility,” code: 0048870) related proteins. (c) Motility paths of *E. coli* K12 treated (6.25 μM **Ian-MPD-1**) and untreated, tracked by microscopy for 10 s (10 fps). Each line represents the path of a bacterium’s movement. (d) Comparison of the distance and velocity of treated (6.25 μM **Ian-MPD-1**) and untreated *E. coli* K12 from motility tracking experiments. Data are shown as the mean + SD of three independent tracking experiments. Statistical relevance is shown based on an unpaired *t* test (**P-value < 0.01, ***P-value < 0.001). (e) Transmission electron microscopy (TEM) experiments. Cultures of *E. coli* K12 in the exponential phase (OD = 0.6) were treated with DMSO or **Ian-MPD-1** (25 μM) for 1 h. Flagellation of bacteria was analyzed using TEM, and exemplary pictures for each condition are depicted. The scale bars represent 1 μm. Statistical analysis of the flagellation status in different TEM pictures (*n* = 248) is shown for each condition.

Given the upregulation of flagellum-associated proteins in our whole-proteome study, we drew our attention to the main part of the bacterial flagellum, FliC. Flagellin is essential for bacterial motility and thus an important virulence trait, i.e. to facilitate host cell invasion.^12,32–36^ Interestingly, a full proteome analysis with a single-gene knockout mutant of *fliC* (Keio collection, JW1908)^31^ in the presence of **Ian-MPD-1** did almost fully abolish the upregulation of cell motility (GO-term: “cell motility,” code: 0048870) related proteins observed in the wildtype strain such as transcriptional regulators and stabilizers RpoN,^37,38^ FliA^39,40^ and RpoZ,^41,42^ swarming motility proteins YcdX^43^ and YcdY,^43,44^ flagellar proteins FliG^45,46^ and FliH,^47^ chemotaxis protein CheY,^48,49^ highlighting the role of **Ian-MPD-1** induced changes (Figure 3b).

To further elucidate the interaction with Ianthelliformisamines on a molecular level, *fliC* was cloned, overexpressed, and FliC was purified for direct validation of **Ian-P-1** binding via ABPP. The FliC protein showed concentration-dependent labeling on fluorescent SDS-PAGE while heat denaturation of FliC abolished this interaction, demonstrating that a folded protein is required for compound binding (Figure S8).

To investigate if **Ian-MPD-1** binding to FliC has a direct impact on bacterial motility, we performed microscopical motility assays based on Pottash et al. (Figure 3c).^50^ Recording the swimming of **Ian-MPD-1** treated (6 μM) and untreated *E. coli* (Supporting Videos 1 and 2) and analyzing the bacterial paths by a Python script revealed a 3.4- and 4.2-fold reduction of velocity and distance, respectively, confirming indeed a major influence of **Ian-MPD-1** on bacterial motility (Figure 3d, Supporting Figure S9). Consistent with a flagella-dependent mechanism, Ian-MPD-1 treatment reduced wild-type motility to levels similar to a Δ*fliC* mutant (Figure S10). Transmission electron microscopy (TEM) analysis comparing **Ian-MPD-1** treated and untreated *E. coli* K12 cells revealed a significant impairment in flagella formation following treatment (Figure 3e). Manual evaluation of 248 cells showed a reduction in the proportion of flagellated cells from 70% in the control group (110/157) to 22% in the treated group (21/91). These results confirm inhibition of flagellum assembly as a major target of **Ian-MPD-1**. Strikingly, both the motility-tracking and TEM experiments were performed in the absence of sodium bicarbonate, under conditions where the compound does not measurably inhibit growth in our assays. While we cannot fully exclude additional downstream contributions, the rapid loss of motility and reduced flagellation observed within 30–60 min are consistent with a primary perturbation of flagellar function rather than a secondary effect of generalized cellular shutdown.

### Ianthelliformisamines Show Therapeutic Potential against Gram-Negative Bacteria

To elucidate the therapeutic potential of Ianthelliformisamines as antibacterials, we first determined the toxicity against human cells. A MTT assay of **Ian-MPD-1** in HeLa cells revealed an IC_50_ of about 60 μM which provides a suitable window for therapeutic applications (Figure 4a). Based on the low toxicity, we commenced with invasion assays of *E. coli* into human cells. Therefore, we switched from the nonpathogenic *E. coli* K12 strain to the uropathogenic *E. coli* CFT073 to enable sufficient invasion into human cells. FoldMason-based alignments of AlphaFold2-predicted models showed structural conservation of FliC (MSA-LDDT: 0.568) and InfA (MSA-LDDT: 1.000) between *E. coli* K12 and CFT073 (Figure S11).^51^ These findings support the use of CFT073 as a structurally representative surrogate for experiments in a pathogenic background. Indeed, **Ian-MPD-1** reduced the invasion of *E. coli* CFT073 in HeLa cells in a concentration-dependent manner (Figure 4b). At a multiplicity of infection of 25 (MOI = 25), the addition of 25 μM **Ian-MPD-1** reduced the intracellular number of bacteria by a factor of 2, demonstrating a significant impact on bacterial pathogenicity.

**Figure 4:**
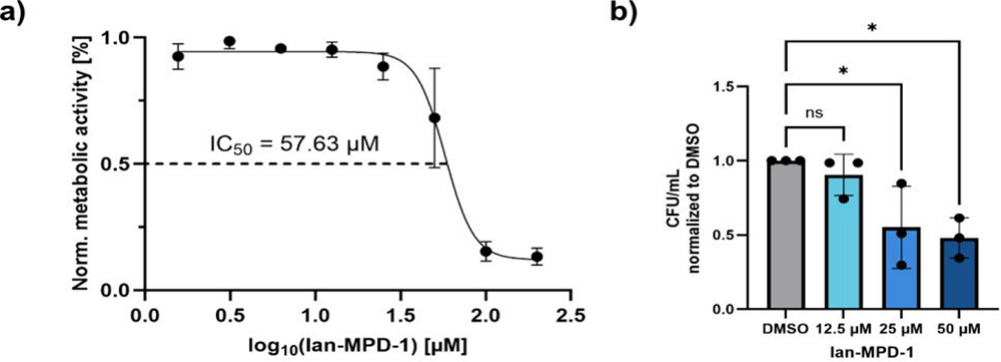
**Ian-MPD-1** exhibits a therapeutic window and reduces bacterial invasion into human cells. (a) MTT-assay of **Ian-MPD-1** in HeLa cells. The remaining metabolic activity was determined relative to DMSO control after 24 h treatment of HeLa cells with increasing concentrations of **Ian-MPD-1**. The experiment was performed in technical sextuplicates, and the data are presented as the mean ± SD of two independent experiments (*n* = 2). (b) Invasion assay of *E. coli* CFT073 into HeLa cells. Invasion is shown in CFU/mL normalized to the DMSO control after infection (MOI = 25) of HeLa cells with *E. coli* CFT073 and treatment with increasing concentrations of **Ian-MPD-1**. The experiment was performed in technical duplicates, and data are shown as mean ± SD of three independent experiments (*n* = 3). Statistical relevance is shown based on an ordinary one-way ANOVA test (ns = *P*-value > 0.05, *P-value < 0.05).

## Conclusions

This study emphasizes the great potential of natural products in combination with chemical proteomics to decipher unprecedented MoAs. Inhibition of ribosomal translation is a hot spot of many natural product-derived antibiotics. Our chemoproteomic data nominate InfA as a putative engagement target of Ianthelliformisamines. To our knowledge, InfA has not previously been reported as a direct antibacterial target, which makes this finding an intriguing entry point for future mechanistic validation and for overcoming existing resistance in this pathway.

Without the use of chemical proteomics, the second, nonessential target, FliC, would have escaped our attention. It is involved in motility, a rather specialized trait that is not often used in the initial characterization of natural products. Thus, based on this knowledge, we demonstrated an inhibition of bacterial swimming in the presence of **Ian-MPD-1** and a major blockage in the assembly of the flagellum.

Both targets synergistically impair the ability of *E. coli* to invade host cells and survive within the host environment as shown by our invasion assay. Overall, this study showcases the potential of dual antibiotic/antivirulence strategies for antibacterial drug development.

## Supplementary Material









## Data Availability

The mass spectrometry proteomics data have been deposited to the ProteomeXchange Consortium via the PRIDE partner repository with the data set identifier PXD070366.^52^

## Notes

DMSO control (MP4)
Ian-MPD-1-treated (MP4)
FliC mutant (MP4)
Processed and summarized data (e.g., MIC values in mutant strains, proteomics results, assay performance, membrane and DNA interactions, SDS PAGE, translation assay, motility tracking experiments) (Figures S1–S11 and Tables S1–S7); experimental details, materials and methods, including biochemical and chemical methods, and NMR spectra (PDF)
