# Supplementary material for: Natural Product-Derived Ianthelliformisamines Inhibit Protein Translation and Block Bacterial Flagellum Assembly
Source: ACS Chem Biol. 2026 Apr 21;21(5):999–1006. doi: 10.1021/acschembio.5c01018 (PMC13184987; doi:10.1021/acschembio.5c01018)

NATURAL PRODUCT-DERIVED IANTHELLIFORMISAMINES INHIBIT PROTEIN TRANSLATION AND BLOCK BACTERIAL FLAGELLUM ASSEMBLY

Max Bottlinger, Martino Morici, Elena Fajardo-Ruiz, Isabella Gantner, Sophie Brameyer, Michael Isselstein, Max Berger, Daniel N. Wilson, Kirsten Jung, Stephan A. Sieber*

[*] M. Bottlinger, S. A. Sieber
Center for Functional Protein Assemblies
Department of Bioscience, TUM School of Natural Sciences
Technische Universität München
Ernst-Otto-Fischer-Straße 8, 85748 Garching, Deutschland.
E-mail: stephan.sieber@tum.de

M. Morici, M. Berger, D. N. Wilson
Institute for Biochemistry and Molecular Biology
University of Hamburg
20146 Hamburg, Germany

E. Fajardo-Ruiz, S. Brameyer, K. Jung
Faculty of Biology, Microbiology
Ludwig-Maximilians-Universität München
82152 Martinsried, Germany

I. Gantner
Faculty of Biology, Plant Development and Electron Microscopy
Ludwig-Maximilians-Universität München
82152 Martinsried, Germany

M. Isselstein
Faculty of Biology
Ludwig-Maximilians-Universität München,
82152 Martinsried, Germany
Center for Geometrically Engineered Cellular Membranes
Department of Chemistry
University of Copenhagen
Copenhagen, Denmark

Table of Content

SUPPLEMENTARY TABLES 4

SUPPLEMENTARY FIGURES 7

EXPERIMENTAL 12

Biochemical Methods 12
Bacterial culture conditions 12
Cell culture 13
Minimal inhibitory concentration (MIC) 13
DNA-shift assay 13
Membrane depolarization assay 13
Membrane Integrity assay 14
MTT assay 14
Time-kill assay 14
Gel-based fluorescent labeling 15
Preparative labeling for Mass spectrometry analysis 15
Full proteome analysis in *E. coli* K12 wt and mutant 17
LC-MS measurements and data analysis Orbitrap Eclipse samples 18
In vitro translation inhibition assay 20
PCR & Cloning 20
FliC Protein overexpression & purification 21
Gel-based Fluorescence labeling of recombinant FliC 22
Microscopical Motility tracking of *E. coli* K12 22
Transmission electron microscopy (TEM) 23
Invasion assay 23

Chemical Synthesis 24
General 24
Reaction control 24
Chromatography 24
NMR spectroscopy 25
High-resolution mass spectrometry 25
3,3'-(hexane-1,6-diylbis(azanediyl))dipropanenitrile (DA-1a) 26
Di-*tert*-butyl hexane-1,6-diylbis((2-cyanoethyl)carbamate) (DA-1b) 26
Di-*tert*-butyl hexane-1,6-diylbis((3-aminopropyl)carbamate) (DA-1) 27
Di-tert-butyl butane-1,4-diylbis((2-cyanoethyl)carbamate) (DA-2a) 28
Di-tert-butyl butane-1,4-diylbis((3-aminopropyl)carbamate) 28
2,4-dibromo-5-methoxybenzaldehyde (CA-1a) 29
Ethyl (*E*)-3-(2,4-dibromo-5-methoxyphenyl)acrylate (CA-1b) 30
(*E*)-3-(2,4-dibromo-5-methoxyphenyl)acrylic acid (CA-1c) 31
2,5-dioxopyrrolidin-1-yl-(*E*)-3-(2,4-dibromo-5-methoxyphenyl)acrylate (CA-1) 32
2,4-dibromo-5-(prop-2-yn-1-yloxy)benzaldehyde (CA-2a) 32
Ethyl (*E*)-3-(2,4-dibromo-5-(prop-2-yn-1-yloxy)phenyl)acrylate (CA-2b) 33
(*E*)-3-(2,4-dibromo-5-(prop-2-yn-1-yloxy)phenyl)acrylic acid (CA-2c) 34
2,5-dioxopyrrolidin-1-yl-(*E*)-3-(2,4-dibromo-5-(prop-2-yn-1-yloxy)phenyl)acrylate (CA-2) 35
Ethyl (*E*)-3-(3,5-dibromo-4-hydroxyphenyl)acrylate (CA-3a) 36
(*E*)-3-(3,5-dibromo-4-hydroxyphenyl)acrylic acid (CA-3b) 36

2,5-dioxopyrrolidin-1-yl-(*E*)-3-(3,5-dibromo-4-methoxyphenyl)acrylate (CA-3) 37
(E)-N-(3-((6-((3-aminopropyl)amino)hexyl)amino)propyl)-3-(2,4-dibromo-5-methoxyphenyl)acrylamide tris trifluoroacetate(**I**an-MPD-1) 38
(*E*)-N-(3-((6-((3-aminopropyl)amino)hexyl)amino)propyl)-3-(2,4-dibromo-5-(prop-2-yn-1-yloxy)phenyl)acrylamide tris trifluoroacetate (**I**an-P-1) 39
(*E*)-N-(3-((4-((3-aminopropyl)amino)butyl)amino)propyl)-3-(3,5-dibromo-4-methoxyphenyl)acrylamide tris trifluoroacetate (**I**an-A) 40
Di-tert-butyl-butane-1,4-diylbis((3-((*E*)-3-(3,5-dibromo-4-methoxyphenyl)acrylamido)propyl)carbamate) 41
(2*E*,2'*E*)-N,N'-((butane-1,4-diylbis(azanediyl))bis(propane-3,1-diyl))bis(3-(3,5-dibromo-4-methoxyphenyl)acrylamide) bis trifluoroacetate (**I**an-C) 42

LITERATURE 42

^{1}H, ^{13}C-NMR SPECTRA 45

Supplementary Tables

Table S1: Minimal Inhibitory concentration [µM] against *E. coli* knockouts deficient in LPS-biosynthesis (ΔRFM795), in efflux (Δ*tolC_bamB*) or in flagellin (Δ*fliC*) in the presence of and without sodium bicarbonate (c = 25 mM). Results are the means of technical replicates and are confirmed in independent measurements.

Derivative / Strain	*E. coli* ΔRFM795		*E. coli* Δ*tolC_bamB*		*E. coli* ΔFliC	
$NaHCO_3$	-	+	-	+	-	+
**Ian-A**	>100	12.5	>100	25	-	-
**Ian-C**	100	6.25	50	6.25	-	-
**Ian-MPD-1**	>100	6.25	>100	6.25	-	6.25
**Ian-P-1**	>100	3.13	>100	6.25	-	-

Table S2: Cell motility (GO-term: "cell motility", code: 0048870) associated proteins from volcano plot S4. *E. coli* K12 cells and *fliC* mutant treated with 6.25 µM **Ian-MPD-1** or DMSO.

Uniprot ID	Gene name	Protein description	Log_2 fold change (wt)	Log_2 fold change (*fliC* mut)
**P31068**	*fliH*	Flagellar assembly protein FliH	1.57	-
**P0AEM6**	*fliA*	RNA polymerase sigma factor FliA (RNA polymerase sigma factor for flagellar operon) (Sigma F) (Sigma-27) (Sigma-28)	1.47	-
**P0A800**	*rpoZ*	DNA-directed RNA polymerase subunit omega (RNAP omega subunit) (EC 2.7.7.6) (RNA polymerase omega subunit) (Transcriptase subunit omega)	1.39	-0.76328
**P0ABZ1**	*fliG*	Flagellar motor switch protein FliG	1.27	0.014712
**P0AE67**	*cheY*	Chemotaxis protein CheY	1.21	-
**P75915**	*ycdY*	Chaperone protein YcdY	1.03	-0.21451
**P22586**	*fliO*	Flagellar protein FliO	0.91	-
**P02942**	*mcp1*	Methyl-accepting chemotaxis protein I (MCP-I) (Serine chemoreceptor protein)	0.89	1.339304
**P0A9H9**	*cheZ*	Protein phosphatase CheZ (EC 3.1.3.-) (Chemotaxis protein CheZ)	0.78	1.292282
**P24255**	*rp54*	RNA polymerase sigma-54 factor	0.74	-0.42249
**P52612**	*fliI*	Flagellum-specific ATP synthase (EC 7.1.2.2)	0.70	-
**P52614**	*fliK*	Flagellar hook-length control protein	0.70	-
**P0ABX2**	*flgC*	Flagellar basal-body rod protein FlgC (Putative proximal rod protein)	0.68	-
**P29744**	*flgL*	Flagellar hook-associated protein 3 (HAP3) (Hook-filament junction protein)	0.56	-
**P75914**	*ycdX*	Probable phosphatase YcdX (EC 3.1.3.-)	0.55	-0.10761

**P0ABW9**	*flgB*	Flagellar basal body rod protein FlgB (Putative proximal rod protein)	0.51	-
**P21361**	*yciG*	Uncharacterized protein YciG	-0.51	-0.53603
**P76575**	*yfgJ*	Uncharacterized protein YfgJ	-0.47	-0.15947
**P24216**	*fliD*	Flagellar hook-associated protein 2 (HAP2) (Filament cap protein) (Flagellar cap protein)	-0.41	-
**P76010**	*ycgR*	Flagellar brake protein YcgR (Cyclic di-GMP binding protein YcgR)	-0.37	6.553696
**P75937**	*flgE*	Flagellar hook protein FlgE	-0.36	-
**P0A8T7**	*rpoC*	DNA-directed RNA polymerase subunit beta' (RNAP subunit beta') (EC 2.7.7.6) (RNA polymerase subunit beta') (Transcriptase subunit beta')	-0.36	-0.04528
**P30176**	*ribX*	N-glycosidase YbiA (EC 3.2.2.-) (Riboflavin biosynthesis intermediates N-glycosidase)	0.35	-0.35318
**P76299**	*flhB*	Flagellar biosynthetic protein FlhB	-0.31	-
**P0A8V2**	*rpoB*	DNA-directed RNA polymerase subunit beta (RNAP subunit beta) (EC 2.7.7.6) (RNA polymerase subunit beta) (Transcriptase subunit beta)	-0.30	0.083094
**P0A6S0**	*flgH*	Flagellar L-ring protein (Basal body L-ring protein)	-0.29	-0.00656
**P0ABX5**	*flgG*	Flagellar basal-body rod protein FlgG (Distal rod protein)	-0.27	-
**P77804**	*ydgA*	Protein YdgA	-0.27	-0.38092
**P0A6S3**	*flgI*	Flagellar P-ring protein (Basal body P-ring protein)	-0.26	-
**P0ABX8**	*fliL*	Flagellar protein FliL	0.26	-
**P0AE39**	*ypdB*	Transcriptional regulatory protein YpdB	-0.24	0.013374
**P06974**	*fliM*	Flagellar motor switch protein FliM	-0.24	-
**P25798**	*fliF*	Flagellar M-ring protein	-0.23	-
**P15070**	*fliN*	Flagellar motor switch protein FliN	0.22	-
**P0AC05**	*fliP*	Flagellar biosynthetic protein FliP	0.22	1.165987
**P07363**	*cheA*	Chemotaxis protein CheA (EC 2.7.13.3)	-0.18	2.326871
**P76298**	*flhA*	Flagellar biosynthesis protein FlhA	-0.14	-0.46663
**P0A7Z4**	*rpoA*	DNA-directed RNA polymerase subunit alpha (RNAP subunit alpha) (EC 2.7.7.6) (RNA polymerase subunit alpha) (Transcriptase subunit alpha)	-0.13	-0.04388
**P37665**	*yiaD*	Probable lipoprotein YiaD	0.12	0.287672
**P09348**	*motA*	Motility protein A (Chemotaxis protein MotA)	0.11	-
**P0AAC0**	*uspE*	Universal stress protein E	-0.10	-0.33238
**P25889**	*preA*	NAD-dependent dihydropyrimidine dehydrogenase subunit PreA (DPD) (EC 1.3.1.1) (Dihydrothymine	-0.05	0.072036

		dehydrogenase) (Dihydrouracil dehydrogenase)		
**P0AEX9**	*malE*	Maltose/maltodextrin-binding periplasmic protein (MMBP) (Maltodextrin-binding protein) (Maltose-binding protein) (MBP)	0.04	-0.85169
**P77333**	*pgrR*	HTH-type transcriptional regulator PgrR (Regulator of PG recycling)	-0.04	0.077903
**P0AF06**	*motB*	Motility protein B (Chemotaxis protein MotB)	-0.04	-
**P0A7G6**	*recA*	Protein RecA (Recombinase A)	0.04	-0.00198
**P75938**	*flgF*	Flagellar basal-body rod protein FlgF (Putative proximal rod protein)	-0.03	-
**P64548**	*yfiR*	Protein YfiR	-0.02	-0.14933

Table S3: Significant hits from the volcano plot in Figure 2b. *E. coli* K12 treated with 6.25 µM **Ian-P-1** or DMSO.

**Uniprot ID**	**Gene name**	**Protein description**	**Log_2 fold change**	**Competition with 4× excess Ian-MPD-1?**
P04949	*fliC*	Flagellin	4.34	Yes
P69222	*infA*	Translation initiation factor IF-1	1.34	Yes
P0AGL7	*rsmE*	Ribosomal RNA small subunit methyltransferase E	1.33	No
P23003	*trmA*	tRNA/tmRNA (uracil-C(5))-methyltransferase	1.09	No
P31063	*yedD*	Uncharacterized lipoprotein YedD	1.02	No
P63228	*gmhB*	D-glycero-beta-D-manno-heptose-1,7-bisphosphate 7-phosphatase	0.99	No

Table S4: Assay performance metrics for the *in vitro* translation assay.

**Metrics**	**Ian-MPD-1**	**Ian-A**	**Ian-C**
**Positive control (n=3)**	1000 µM	1000 µM	1000 µM
**Mean [%] + SD**	0.0051 ± 0.0006	0.0080 ± 0.0017	0.0096 ± 0.0058
**Negative control (n=3)**	0 µM	0 µM	0 µM
**Mean + SD**	100 ± 0.0	100 ± 0.0	100 ± 0.0
**Z'**	0.99998	0.99995	0.99982

Supplementary Figures

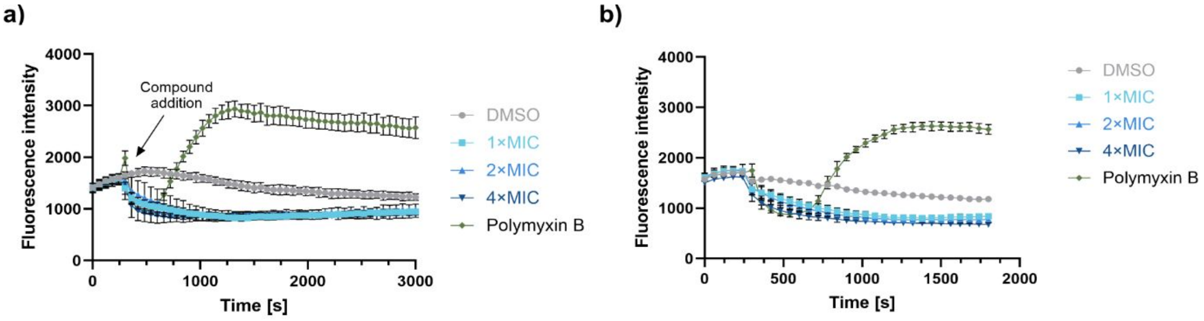

Figure S1: Evaluation of cell membrane depolarization. Membrane potential assay in *E. coli* K12. Cells are treated with a) **lan-A** or b) **lan-C**, and the resulting fluorescence intensity of membrane potential-sensitive dye 3,3′-dipropylthiadicarbocyanine iodide ($DiSC_3(5)$) is measured. DMSO and Polymyxin B are used as negative or positive controls, respectively. Data represent the mean + SD of three technical replicates and are representative of at least two independent biological replicates.

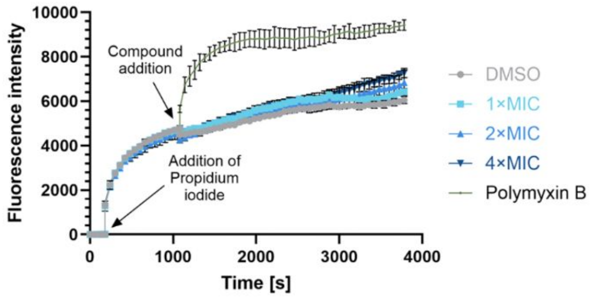

Figure S2: Membrane integrity assay: *E. coli* K12 cells are treated with **lan-MPD-1** and resulting fluorescence intensity of DNA-intercalating dye propidium iodide is measured. DMSO and Polymyxin B are used as negative or positive controls, respectively. Data represent the mean + SD of three technical replicates and are representative of at least two independent biological replicates.

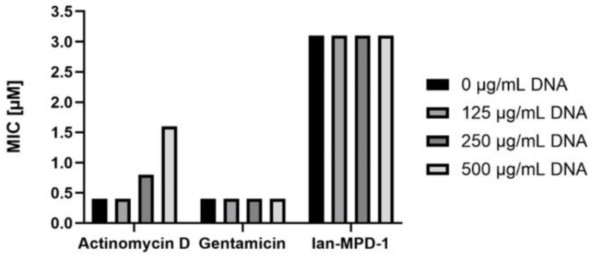

Figure S3: DNA-shift-assay. The MICs of Actinomycin D, Gentamicin and **lan-MPD-1** against *E. coli* K12 were determined in the presence of external DNA (0-500 µg/mL). The depicted data represent averaged technical duplicates.

a)

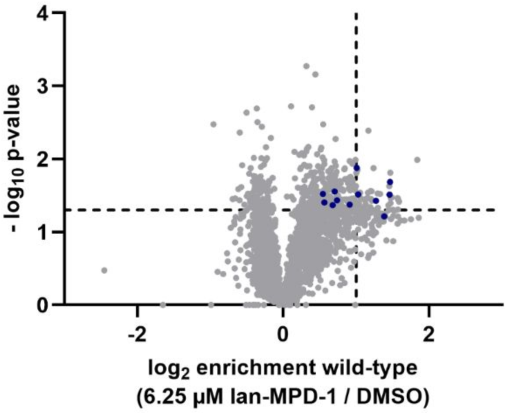

b)

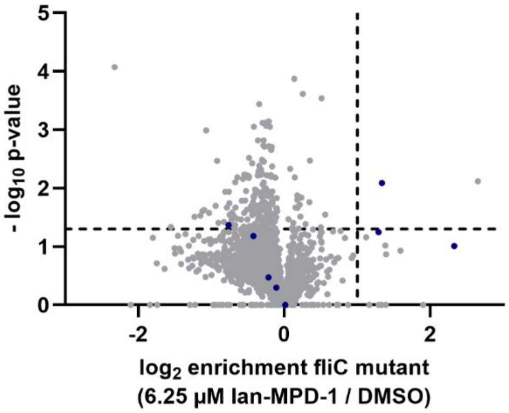

Figure S4: Full proteome experiments of treated wild-type *E. coli* and *fliC* mutant (Keio collection, JW1908). **a)** Volcano plot of *E. coli* K12 cells treated with 6.25 µM **lan-MPD-1** compared to DMSO (1%). Dotted lines indicate significance cutoff p-value ≤ 0.05 (n = 4) and log2 fold change ≥ 1. Upregulated proteins highlighted in blue: Cell motility (GO-term: “cell motility”, code: 0048870) related proteins. b**)** Volcano plot of *E. coli fliC* mutant cells treated with 6.25 µM **lan-MPD-1** compared to DMSO (1%). Dotted lines indicate significance cutoff p-value ≤ 0.05 (n = 4) and log2 fold change ≥ 1. Proteins highlighted in blue: Cell motility (GO-term: “cell motility”, code: 0048870) related proteins.

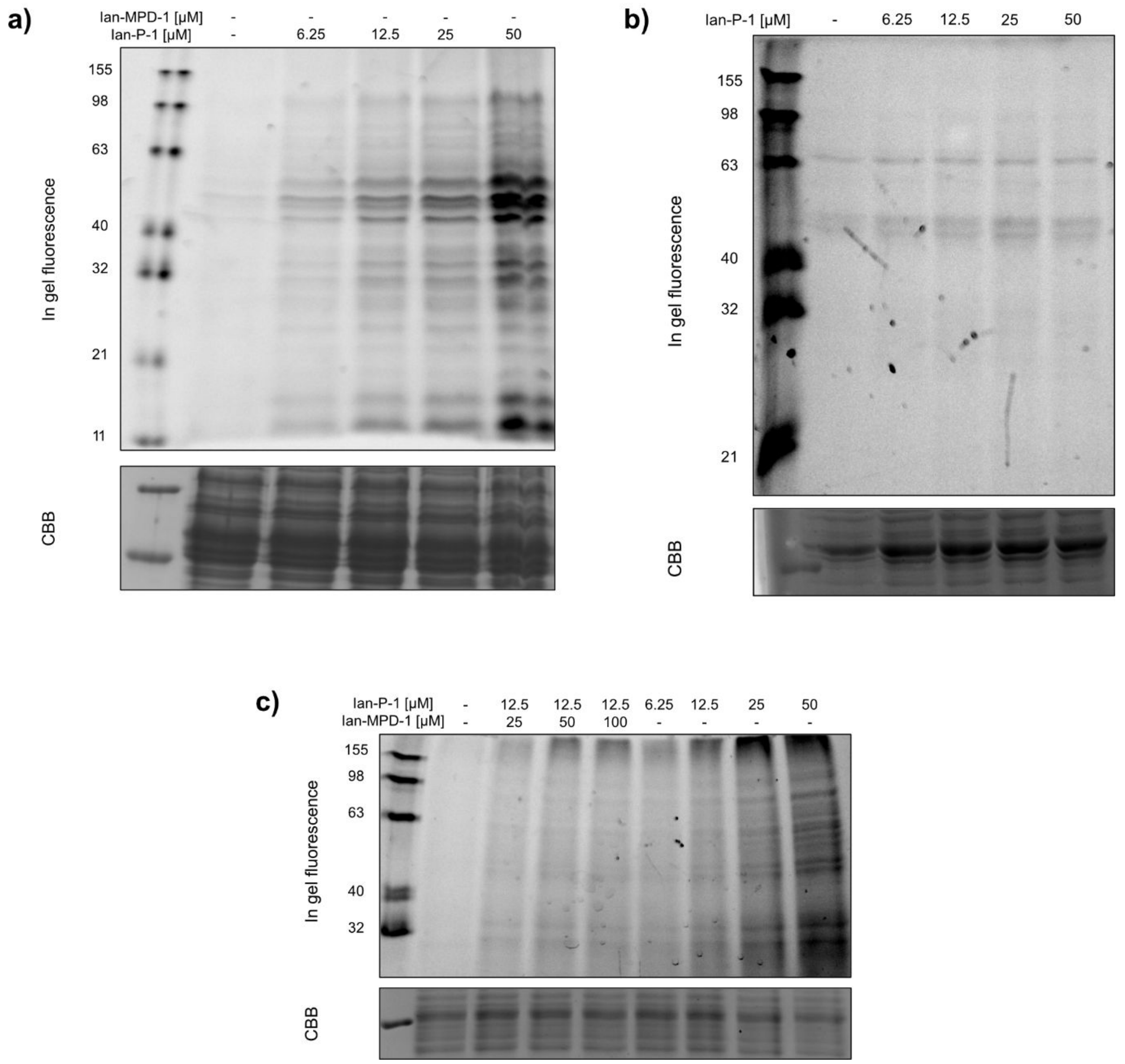

Figure S5: Gel-based ABPP with **Ian-P-1**. a) Live *E. coli* K12 cells were treated for 1h with increasing concentrations of **Ian-P-1** in the presence of sodium bicarbonate, lysed, and clicked to rhodamine azide. In-gel fluorescence shows concentration-dependent labeling with increasing concentrations of **Ian-P-1**. Coomassie blue staining (CBB) as a loading control is depicted. The experiment was repeated in two independent experiments. b) Live *E. coli* K12 cells were treated for 1h with increasing concentrations of **Ian-P-1** (in the absence of sodium bicarbonate), lysed, and clicked to rhodamine azide. In-gel fluorescence shows no detectable labeling of **Ian-P-1** compared to the DMSO control. Coomassie blue staining (CBB) as a loading control is depicted. c) *S. aureus* SH1000 lysate (1 mg/mL in PBS) was treated for 1h at 37 °C with increasing concentrations of Ian-P-1 and/or Ian-MPD-1 (in the absence of sodium bicarbonate) and clicked to rhodamine-azide. In-gel fluorescence shows concentration-dependent labeling with increasing concentrations of **Ian-P-1**. Coomassie blue staining (CBB) as a loading control is depicted.

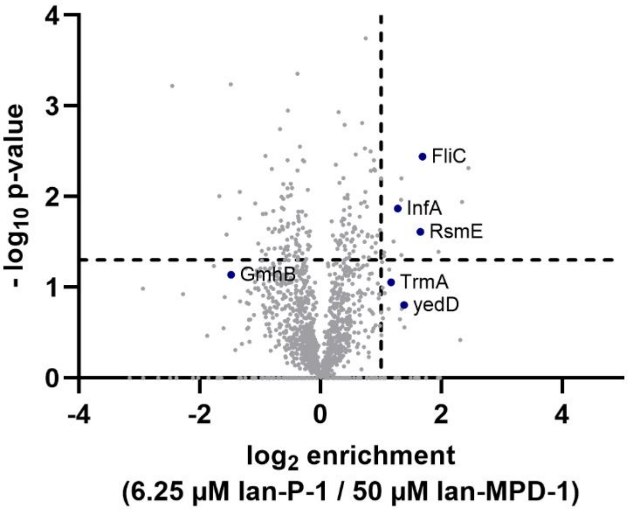

Figure S6: Volcano plot of *E. coli* K12 cells treated with 6.25 µM **lan-P-1** (alkynylated probe) compared to 6.25 µM **lan-P-1** in competition with 50 µM **lan-MPD-1** (parent compound). Proteins enriched in Figure 2b) and competed here are shown by name (InfA, FliC, RsmE). All other proteins were not enriched in b). Dotted lines indicate significance cutoff p-value ≤ 0.05 (n = 4) and $\log_2$ fold change ≥ 1. Proteins significantly enriched in Figure 2b) are highlighted in blue.

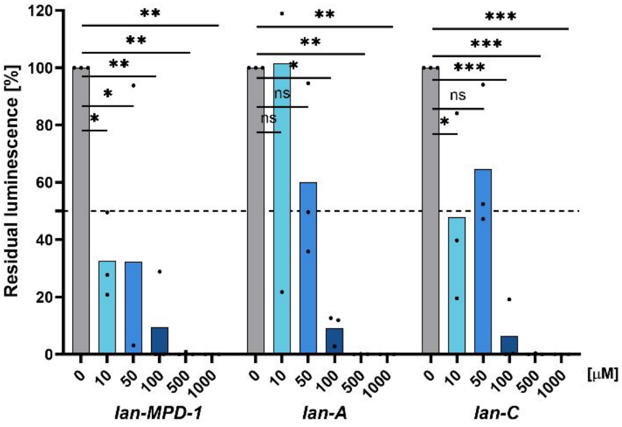

Figure S7: Concentration-dependent *in vitro* assay in the absence and presence of **lan-MPD-1**, **lan-A**, and **lan-C** with an *E. coli* cell-free lysate-based translation system expressing a firefly luciferase (Fluc) reporter. The assay was performed in a buffer system containing sodium bicarbonate (25 mM). Experiments were performed in three independent replicates. Statistical relevance is shown based on a paired one-way ANOVA test (* = p-value < 0.02, ** = p-value < 0.01, *** = p-value < 0.001, ns = non-significant).

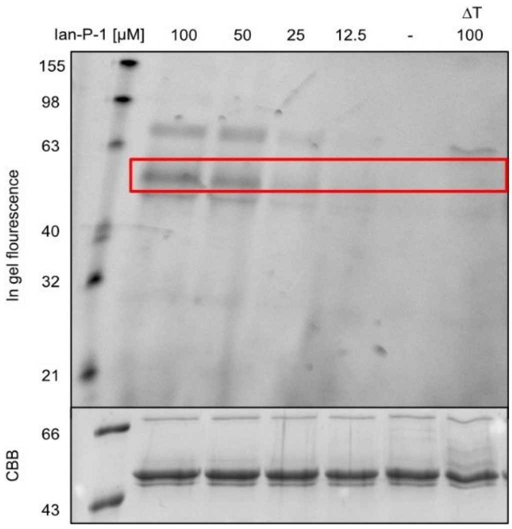

Figure S8: Gel-based ABPP of recombinant purified FliC. Concentration-dependent labeling of FliC with increasing concentrations of **Ian-P-1** and abolished binding in the heat-denatured control sample. Coomassie blue staining (CBB) as a loading control is depicted. Experiment was repeated in two independent experiments. Heat-denatured samples were heated to 95 °C in a buffer containing 0.4% SDS prior to probe-labeling.

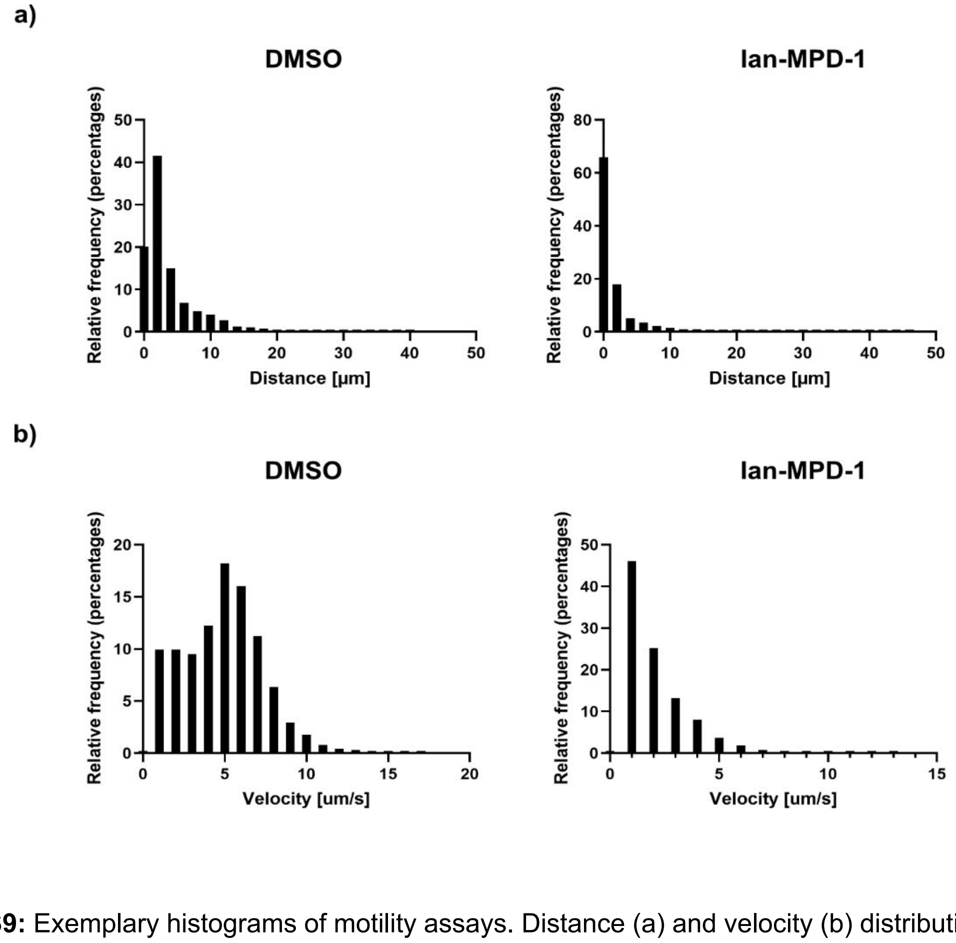

Figure S9: Exemplary histograms of motility assays. Distance (a) and velocity (b) distribution of DMSO or Ian-MPD-1 (6.25 µM) treated *E. coli* K12. The histograms represent an exemplary distribution of the tracking of one biological replicate at three different fields of view (FOVs) of the microscopy slide. The histograms are representative of all of the biological replicates (n = 3).

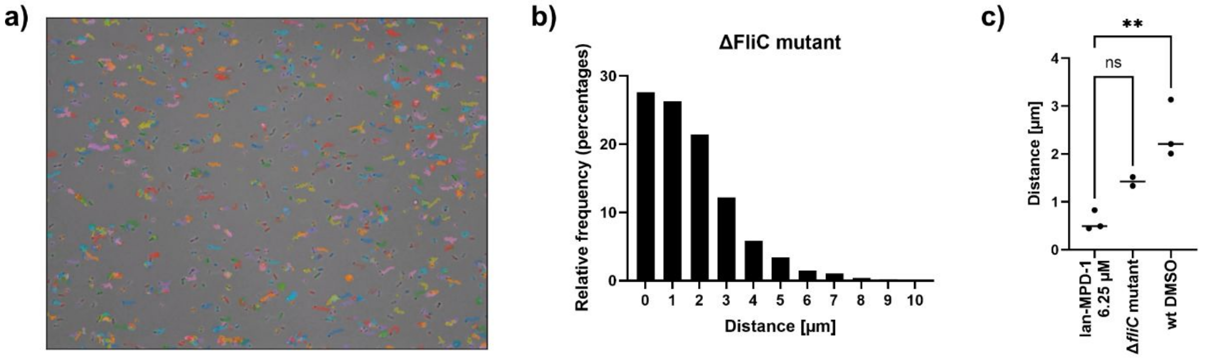

Figure S10: Comparison of motility between Ian-MPD-1 treated/untreated wild-type and *fliC* mutant (Keio collection, JW1908) cells. a) Motility paths of *E. coli fliC* mutant (+1% DMSO) tracked by microscopy for 10 s (10 fps). Each line represents the path of a bacterium's movement. b) Exemplary histograms of motility assays. Distance distribution of DMSO treated *E. coli fliC* mutant. The histograms represent an exemplary distribution of the tracking of one biological replicate at three different fields of view (FOVs) of the microscopy slide. The histograms are representative of all of the biological replicates (n = 2). c) Comparison of the distance of treated (6.25 µM Ian-MPD-1) and untreated *E. coli* K12 as well as untreated *E. coli fliC* mutant from motility tracking experiments. Data are shown as the mean + SD of at least two independent tracking experiments. Statistical relevance is shown based on an unpaired ordinary one-way ANOVA test (** = p-value < 0.01, ns = non-significant).

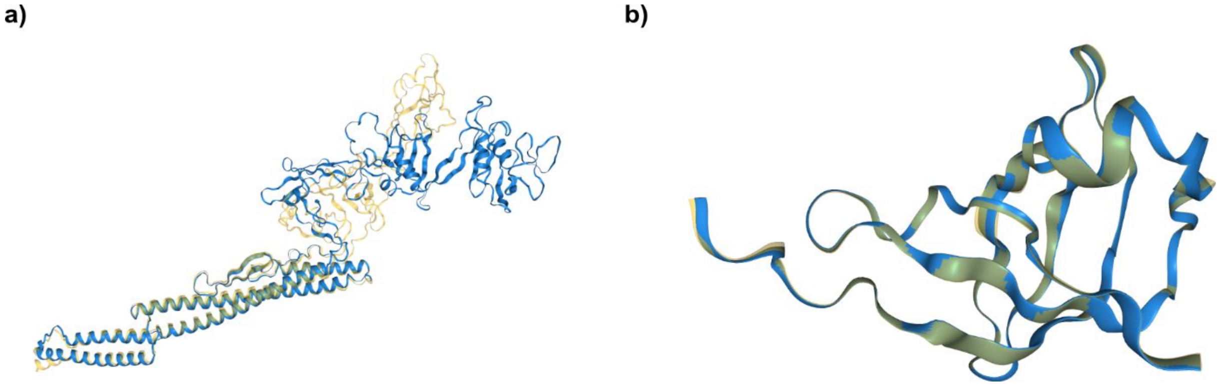

Figure S11: FoldMason[1] structural alignment of AlphaFold predicted structures of FliC and InfA in *E. coli* K12 and CFT073. a) The FoldMason alignment of FliC shows a moderate degree of structural similarity with an MSA-LDDT of 0.568. b) The FoldMason alignment on *InfA* shows a high degree of structural similarity with an MSA-LDDT of 1.

Experimental

Biochemical Methods

Bacterial culture conditions

5 mL pre-cultures were inoculated with 5 µL of a bacterial glycerol stock and grown at 37 °C, 200 rpm, overnight. Unless stated otherwise *E. coli* K12, *E. coli* CFT073, *E. coli fliC* mutant (clone JW1908, cat. OEC4987-213605791, *Horizon Discovery Biosciences Limited*) and *Pseudomonas aeruginosa* PAO1 were cultivated in Lysogeny broth (LB, 10 g/L casein peptone, 5 g/L NaCl, 5 g/L yeast extract). *S. aureus* USA300 was cultivated in Lysogeny broth (B, 10 g/L casein peptone, 5 g/L NaCl, 5 g/L yeast extract, 1 g/L K_2HPO_4). *E. faecalis* V583 was cultivated in brain heart infusion medium (BHB,

7.5 g/L brain infusion, 10 g/L heart infusion, 10 g/L casein peptone, 5 g/L NaCl, 2.5 g/L Na_2HPO_4, 2 g/L glucose).

Cell culture

HeLa cells were cultured in Dulbecco's Modified Eagle Medium (DMEM, high glucose, 4.5 g/L) supplemented with 10% fetal bovine serum (FBS, heat-inactivated) and 2 mM L-glutamine at 37 °C in a 5% CO_2 atmosphere.

Minimal inhibitory concentration (MIC)

Broth microdilution assay was used to determine the minimal inhibitory concentration (MIC). A sterile flat bottom 96-well plate was used and the outer wells were filled up with 200 µL media. 2 µL of the compounds were added from a DMSO stock to 98 µL of media and a serial dilution (1:1) was performed in the wells with 50 µL of media containing 2% DMSO (optional: 50 mM sodium bicarbonate). Finally, the bacterial pre-cultures were diluted (1:10000) in the respective media and 50 µL of the inoculum were added to the wells to get a final volume of 100 µL. Sterile and growth control were included on each well plate. Plates were incubated at 37 °C, 200 rpm for 24 h. The lowest concentration in the dilution series without any microbial growth (indicated by turbidity) was determined as MIC. MIC values were determined in technical replicates and confirmed by two independent biological replicates. If the MIC varied between biological replicates, concentration ranges are given.

DNA-shift assay

The assay was adapted from a literature known protocol.[2] Low molecular weight salmon sperm DNA (Merck) was dissolved in TE buffer (10 mM Tris-HCl, 0.1 mM EDTA, pH = 8). The assay was performed analogously to the above described MIC assay with or without the addition of DNA (0, 125, 250, 500 µg/mL) in the presence of sodium bicarbonate (25 mM). Actinomycin D was included as a positive control, and gentamicin as a negative control. The experiment was conducted in two technical replicates and two independent biological replicates.

Membrane depolarization assay

The assay was adapted from a literature known protocol.[3] A pre-culture of *E. coli* K12 was diluted (1:100) in LB media supplemented with 25 mM sodium bicarbonate. Cells were grown (37 °C, 200 rpm) until OD_{600} = 0.4-0.6. Cells were harvested (6000 g, 10 min, 4 °C) and washed with assay buffer (5 mM HEPES, 5 mM Glucose, pH = 7.2-7.4). The bacteria were resuspended in assay buffer to get OD_{600} = 0.6. 100 mM potassium chloride (2.5 M in 5 mM HEPES, pH = 7.4-7.6) and 0.5 mM EDTA (50 mM in 5 mM HEPES, pH = 7.4-7.6) were added. 3,3'-dipropylthiadicarbo-cyanide iodide (DiSC3(5), 100 µM in DMSO) was added to a final concentration of 1 µM and the bacteria were incubated (37 °C, 200 rpm) for 30-45 minutes in the dark. 99 µL of the bacterial suspension was added to each well of a black 96 well plate and the fluorescence (λ_{ex} = 610 nm, λ_{em} = 660 nm) was measured for 3 minutes at 37 °C in an Infinite M200 Pro (*Tecan* group) microplate reader. 1 µL of DMSO or compound stock (1×, 2× or 4× MIC

final concentration) or the positive control Polymyxin B (7 µM final concentration) was added. The fluorescence (λ_{ex} = 610 nm, λ_{em} = 660 nm) was measured for 30 minutes at 37 °C. Fluorescence quenching of the compounds was excluded by control measurements with the compounds in the absence of bacteria. Measurements were performed in technical triplicate and repeated twice in independent experiments.

Membrane Integrity assay

The assay was adapted from a literature known protocol.[3] A pre-culture of *E. coli* K12 was diluted (1:100) in LB media supplemented with 25 mM sodium bicarbonate. Cells were grown (37 °C, 200 rpm) until OD_{600} = 0.4-0.6. Cells were harvested (6000 g, 10 min, 4 °C) and washed with assay buffer (5 mM HEPES, 5 mM Glucose, pH = 7.2-7.4). The bacteria were resuspended in assay buffer to get OD_{600} = 0.6. 98 µL of the bacterial suspension was added to each well of a black 96 well plate and 1 µL of propidium iodide (1 mM in DMF) was added. The fluorescence (λ_{ex} = 535 nm, λ_{em} = 617 nm) was measured for 3 minutes at 37 °C in an Infinite M200 Pro (*Tecan* group) microplate reader. 1 µL of DMSO or compound stock (1×, 2× or 4× MIC final concentration) or the positive control Polymyxin B (7 µM final concentration) was added. Fluorescence (λ_{ex} = 535 nm, λ_{em} = 617 nm) was measured for 30 minutes at 37 °C. Fluorescence quenching of the compounds was excluded by control measurements with the compounds in the absence of bacteria. Measurements were performed in technical triplicate and repeated twice in independent experiments.

MTT assay

HeLa cells were seeded at a density of 5000 cells per well in a transparent, sterile flat-bottomed 96-well plate (200 µL medium per well). The cells were grown overnight (37 °C, 5% CO_2) to allow the cells to adhere on the surface of the well plate. The medium was aspirated and 100 µL of fresh DMEM medium without FBS supplemented with compound ranging from 1.5 µM to 200 µM (DMSO content 1%) or DMSO (1%) was added. After incubation for 24h (37 °C, 5% CO_2) 20 µL of -(4,5-dimethyl-2-thiazolyl)-2,5-diphenyl-2H-tetrazolium bromide solution (MTT, 5 mg/mL in PBS) was added to each well and the cells were incubated for 4h (37 °C, 5% CO_2). The medium was carefully aspirated, and the resulting formazan crystals were dissolved in DMSO (200 µL per well) by shaking (300 rpm, 10 min). Absorbance at 570 nm with a reference wavelength of 630 nm was recorded using an Infinite F200 pro (*Tecan* group) plate reader. The measured values were normalized to the DMSO-treated controls (100% metabolic activity). Log(inhibitor) vs. response variable slope (four parameters) non-linear regression curves were fitted using Prism (*GraphPad*, V. 10.01) and cytotoxicity is reported as IC_{50} value. Experiments were performed in six technical replicates and repeated twice in independent biological replicates.

Time-kill assay

The assay was adapted from a literature known protocol.[4,5] A pre-culture of *E. coli* K12 was diluted to 1.5 × 10^5 cells/mL into fresh LB medium containing 25 mM sodium bicarbonate. 2 mL of the bacterial suspension was aliquoted to a culture tube and

DMSO (1%) or compound stock (2× or 4× MIC final concentration) was added. Cells were incubated (37 °C, 200 rpm) and serial dilutions were plated on LB agar plates containing 25 mM sodium bicarbonate at indicated time points for the determination of viable cells (CFU/mL). CFU determination was done in three technical triplicates, and the experiments were repeated twice in independent biological replicates.

Gel-based fluorescent labeling

50 mL LB medium was inoculated by a pre-culture of *E. coli* K12 (1:100) and grown for 8 h to OD_{600} = 4.5. The cells were harvested (6000 g, 10 min, 4 °C) and washed with PBS (15 mL). The bacterial pellet was resuspended in M9 minimal media supplemented with 25 mM sodium bicarbonate to get a theoretical OD_{600} = 25. 100 µL of the bacterial suspension was aliquoted to a microcentrifuge tube, and 1 µL of a 100× probe stock or DMSO (1%) was added. The bacterial suspension was incubated (37 °C, 200 rpm) for 1h, harvested (6000 g, 10 min, 4 °C) and washed with PBS (2 × 500 µL each). The cell pellet was reconstituted in 150 µL lysis buffer (0.5% SDS, 1% Triton X-100 in PBS) by sonication (10 s, 10% intensity, Sonopuls HD 2070 ultrasonic rod, *Bandelin electronic* GmbH) and transferred into bead-mill lysis tubes filled with 0.1 mm zirconium beads. The cells were lysed at 6500 rpm for 3 × 30 s using a precellys 24 bead beater (*peqlab*). The lysate was centrifuged (13000 g, 10 min) and the supernatant (120 µL) was transferred into a microcentrifuge tube and centrifuged (21000 g, 10 min). The supernatant (100 µL) was transferred into a new centrifuge tube and 12 µL of click mix (2 µL 5 mM rhodamine azide in DMSO, 2 µL 50 mM $CuSO_4$ in H_2O, 2 µL 50 mM Tris(2-carboxyethyl)phosphine (TCEP) in H_2O and 6 µL 1.67 mM tris(benzyltriazoylmethyl)amine (TBTA) in 80% *t*-BuOH and 20% DMSO) was added per sample. The reaction was incubated (500 rpm, rt) for 1h and quenched by the addition of 600 µL ice-cold acetone. The mixture was stored overnight at -20 °C and pelletized (13000 g, 20 min, 4 °C). The pellet was reconstituted in 1× Laemmli buffer by sonication (10 s, 10% intensity) and analyzed *via* SDS-PAGE. The fluorescence was recorded in a Fujifilm Las-4000 Luminescent Image Analyser with a Fujinon VRF43LMD3 and a 75DF20 filter.

Preparative labeling for Mass spectrometry analysis

Labeling, lysis and click. 4 × 50 mL LB medium was inoculated by 4 different pre-cultures of *E. coli* K12 (1:100) and grown for 8 h to OD_{600} = 4.8. The cells were harvested (6000 g, 10 min, 4 °C) and washed with M9 minimal medium (15 mL). The bacterial pellet was resuspended in M9 minimal media supplemented with 25 mM sodium bicarbonate to get a theoretical OD_{600} = 20. 198 µL of the bacterial suspension was aliquoted to a microcentrifuge tube, and 2 µL of a 100× Ian-P-1 stock, 100× Ian-MPD-1 stock or DMSO (1%) was added. Each treatment condition was conducted in four independent replicates. The bacterial suspension was incubated (37 °C, 200 rpm) for 1h, harvested (6000 g, 10 min, 4 °C) and washed with PBS (2 × 500 µL each). The cell pellet was reconstituted in 130 µL lysis buffer (0.5% SDS, 1% Triton X-100 in PBS) by sonication (10s, 10% intensity, Sonopuls HD 2070 ultrasonic rod, *Bandelin electronic* GmbH) and transferred into bead-mill lysis tubes filled with 0.1 mm zirconium

beads. The cells were lysed at 6500 rpm for 3 × 30 s using a precellys 24 bead beater (*peqlab*). The lysate was centrifuged (13000 g, 10 min) and the supernatant (90 µL) was transferred into a microcentrifuge tube and centrifuged (21000 g, 10 min). The supernatant (80 µL) was transferred into a new centrifuge tube and protein concentration was determined by BCA assay (Roti Quant, *Roth*). The protein concentration of each sample was adjusted to 2.25 mg/mL in a total volume of 40 µL in a 96-well plate (Polypropylene, V-bottom, *Greiner* cat. 651201) and 6.1 µL click-mix (1.2 µL 10 mM biotin-azide in DMSO, 2.5 µL 1.67 mM tris(benzyltriazoylmethyl)amine (TBTA) in 80% *t*-BuOH and 20% DMSO, 1.2 µL 50 mM $CuSO_4$ in H_2O, and 1.2 µL 50 mM Tris(2-carboxyethyl)phosphine (TCEP) in H_2O) was added per sample. The reaction was incubated (950 rpm, rt) for 1.5h and quenched by the addition of 65 µL 8 M urea supplemented with 10 mM TCEP and 20 mM iodoacetamide (IAA) per sample. After 15 minutes at 25 °C, 950 rpm the reaction was quenched by adding 2 µL dithiothreitol (DTT, 500 mM in H_2O) per sample.

Enrichment and Digestion. All reagents used were LC-MS grade. The sample processing is adapted from Mostert *et al.*[6] 10 µL of a 2× concentrated 1:1 mix of washed (3× H_2O) hydrophobic and hydrophilic carboxylate-coated magnetic beads (Cytiva) was added to each sample. To precipitate the proteins onto the beads, 175 µL of ethanol was added to each sample. The plate was now placed into an automated liquid handling system (*Hamilton* Microlab Prep) for further processing. The plate was incubated for 5 minutes, 500 rpm. For each washing step, the plate was placed onto a 96-well ring magnet (Alpaqua, Magnum FLX) for 90 s and the supernatant was removed with a low draw speed (20 µL/s) in order to not remove any beads. The plate was then removed from the magnet and the next washing solution was added, followed by 1 min shaking at 800 rpm. In this way, the samples were washed 3x with 180 µL 80% ethanol and once with 180 µL acetonitrile. To elute the proteins from the carboxyl-coated beads, 75 µL 0.2% SDS in PBS was added to the samples and the plate was incubated for 5 minutes at 40°C, while shaking at 800 rpm. The plate was then placed on the magnet and the supernatant was transferred into new wells. This step was repeated and the additional 75 µL of eluted proteins was added to yield 150 µL of eluted proteins in new wells. Meanwhile, streptavidin magnet beads (*New England Biolabs*, cat# S1420S) were washed 3× with 0.2 % SDS in PBS. 50 µL of the washed streptavidin beads were added to each well containing eluted protein sample. The plate was taken out of the liquid handling system, sealed, and incubated for 1 h at 25 °C, 950 rpm in a plate shaker, with a heated lid to prevent condensation. This allowed the binding of labelled proteins to the streptavidin beads. Afterwards, the plate-seal was removed, and the plate was placed back into the liquid handling system. The beads were washed 3× with 180 µL 0.1% NP-40 in PBS, 2x with 180 µL 6 M urea and 3× with 200 µL H_2O. The bead-bound proteins were digested in 100 µL 50 mM Triethylammonium bicarbonate (TEAB) with 0.5 µg Trypsin (sequencing grade, *Promega*) overnight at 37 °C in a tightly sealed plate and with a heated lid at 950 rpm. After the tryptic digest, the peptides were eluted from the beads in the liquid handling system and subsequently desalted. For this, the

samples were loaded on pre-equilibrated stage tips with two layers of SDP-RPS (*Empore*, 3M). The desalting was performed as previously described by *Coscia et al*. with some minor alterations.[7] In short, the stage-tips were equilibrated with 150 µL wash buffer 1 (1% (v/v) trifluoroacetic acid in isopropanol) before loading the samples. The samples were loaded at 500 g for 10 minutes, followed by a wash step with 170 µL wash buffer 1 (800 g, 10 min) and another washing step with 170 µL wash buffer 2 (0.2% (v/v) trifluoroacetic acid in H_2O). The peptides were eluted with 50 µL elution buffer (1% ammonia, 80% acetonitrile) by centrifugation at 300 g for 5 min, followed by 800 g for 5 min. Samples were dried in a centrifugal evaporator and reconstituted in 25 µL 1% formic acid for LC-MS measurements on an Orbitrap Eclipse Tribrid instrument (*Thermo Fisher Scientific*) in data-independent acquisition.

Full proteome analysis in *E. coli* K12 wt and mutant

4 × 20 mL LB medium was inoculated by 4 different pre-cultures of *E. coli* K12 or *E. coli fliC* mutant (1:100) and grown for 8 h to OD_{600} = 4.8. The cells were harvested (6000 g, 10 min, 4 °C) and washed with M9 minimal medium (15 mL). The bacterial pellet was resuspended in M9 minimal media supplemented with 25 mM sodium bicarbonate to get a theoretical OD_{600} = 20. 198 µL of the bacterial suspension was aliquoted to a microcentrifuge tube, and 2 µL of a 100× Ian-MPD-1 stock or DMSO (1%) was added. Each treatment condition was conducted in four independent replicates. The bacterial suspension was incubated (37 °C, 200 rpm) for 1h, harvested (6000 g, 10 min, 4 °C) and washed with PBS (2 × 500 µL each). The cell pellet was reconstituted in 130 µL lysis buffer (0.5% SDS, 1% Triton X-100 in PBS) by sonication (10 s, 10% intensity, Sonopuls HD 2070 ultrasonic rod, *Bandelin electronic* GmbH) and transferred into bead-mill lysis tubes filled with 0.1 mm zirconium beads. The cells were lysed at 6500 rpm for 3 × 30 s using a precellys 24 bead beater (*peqlab*). The lysate was centrifuged (13000 g, 10 min) and the supernatant (90 µL) was transferred into a microcentrifuge tube and centrifuged (21000 g, 10 min). The supernatant (80 µL) was transferred into a new centrifuge tube and protein concentration was determined by BCA assay (Roti Quant, *Roth*). 50 µg of proteome was transferred to a 96-well plate (Polypropylene, V-bottom, *Greiner* cat. 651201) and filled up with lysis buffer to 50 µL volume. Samples were alkylated with 10 mM TCEP and 20 mM iodoacetamide (IAA). 10 µL of a 1:1 mix of washed (3× H_2O) hydrophobic and hydrophilic carboxylate-coated magnetic beads (*Cytiva*) were added to each sample. To precipitate the proteins onto the beads, 175 µL of ethanol was added to each sample. The plate was now placed into an automated liquid handling system (*Hamilton* Microlab Prep) for further processing. The plate was incubated for 5 minutes, 500 rpm. For each washing step, the plate was placed onto a 96-well ring magnet (*Alpaqua*, Magnum FLX) for 90 s and the supernatant was removed with a low draw speed (20 µL/s) in order to not remove any beads. The plate was then removed from the magnet, and the next washing solution was added, followed by 1 min of shaking at 800 rpm. In this way, the samples were washed 3× with 180 µL 80% ethanol and once with 180 µL acetonitrile. The bead-bound proteins were digested in 100 µL 50 mM TEAB with 0.5 µg Trypsin (sequencing grade, *Promega*) overnight at 37 °C in a tightly sealed plate and with a heated lid at

950 rpm. After the tryptic digest, the peptides were eluted from the beads in the liquid handling system, the beads were washed with 40 µL of 3.5% formic acid and the resulting 140 µL were subsequently desalted. The desalting was performed as previously described by *Coscia et al*. with some minor alterations.[7] In short, the stage-tips were equilibrated with 150 µL wash buffer 1 (1% (v/v) trifluoroacetic acid in isopropanol) before loading the samples. The samples were loaded at 500 g for 10 min, followed by a wash step with 170 µL wash buffer 1 (800 g, 10 min) and another washing step with 170 µL wash buffer 2 (0.2% (v/v) trifluoroacetic acid in H_2O). The peptides were eluted with 50 µL elution buffer (1% ammonia, 80% acetonitrile) by centrifugation at 300 g for 5 minutes, followed by 800 g for 5 minutes. Samples were dried in a centrifugal evaporator and reconstituted in 25 µL 0.1% trifluoroacetic acid for LC-MS measurements on an Orbitrap Eclipse Tribrid instrument (*Thermo Fisher Scientific*) in data-independent acquisition.

LC-MS measurements and data analysis Orbitrap Eclipse samples

Peptide quantification was performed using an HPLC-MS/MS system consisting of a Vanquish Neo UHPLC (*Thermo Fisher Scientific*) coupled to an Orbitrap Eclipse Tribrid instrument (*Thermo Fisher Scientific*). The Vanquish Neo UHPLC was equipped with a PepMap™ Neo.

Enriched samples. 5 µm C18 300 µm × 5 mm Trap Cartridge (*Thermo Fisher Scientific*) and operated in a Trap-and-Elute-Injection mode whereby the samples were loaded to the cartridge before the separation started. The system was run with a flow rate of 400 nL/min with buffer A (0.1% formic acid (FA) in water) and buffer B (0.1% FA in acetonitrile). Peptide separation is achieved using an Aurora UltimateTM separation column (3rd generation, 25 cm, nanoflow UHPLC compatible, *ionoptics*) at 40 °C coupled to a Nanospray Flex Ion Source (*Thermo Fisher Scientific*). The HPLC method comprises 45 min and starts with a gradient from 5% to 22% buffer B over a period of 30 min, followed by a second gradient up to 32% buffer B within 5 min and an isocratic period of 10 min at 90% buffer B. Separation column washing and equilibration are conducted with enabled fast equilibration, equilibration factor “3” at 5% buffer B. Trap column washing and equilibration are conducted with enabled fast wash and equilibration together with zebra wash (2 wash cycles, automatic equilibration factor). The Orbitrap Eclipse mass spectrometer was run with an internal real-time mass calibration using a user-defined lock mass (positive, m/z = 445.12003) and operated in data-independent acquisition mode. The full MS scans were collected in the Orbitrap at a resolution of 60000 and an AGC target of 4e5 with a maximum injection time of 100 ms in a scan range of 400-1000 m/z. MS2 spectra were collected at a resolution of 15,000 and an AGC target of 1e6 with a maximum injection time of 40 ms. The isolation in the quadrupole was conducted with windows of 10 m/z with an overlap of 1 m/z in a scan range of 145-1450 m/z. Higher-energy collision-induced dissociation (HCD) with a normalized collision energy of 30% was used to generate the fragments that were detected in the Orbitrap. Data acquisition was performed using Thermo Scientific Foundation software (version 3.1sp9) and Xcalibur (version 4.6).

Full proteome samples. 5 µm C18 300 µm × 5 mm Trap Cartridge (*Thermo Fisher Scientific*) and operated in a Trap-and-Elute-Injection mode whereby the samples were loaded to the cartridge before the separation started. The system was run with a flow rate of 400 nL/min with buffer A (0.1% formic acid (FA) in water) and buffer B (0.1% FA in acetonitrile). Peptide separation is achieved using an Aurora UltimateTM separation column (3rd generation, 25 cm, nanoflow UHPLC compatible, *ionoptics*) at 40 °C coupled to a Nanospray Flex Ion Source (*Thermo Fisher Scientific*). The HPLC method comprises 45 min and starts with a gradient from 5% to 28% buffer B over a period of 30 min, followed by a second gradient up to 35% buffer B within 5 min and an isocratic period of 10 min at 90% buffer B. Separation column washing and equilibration are conducted with enabled fast equilibration, equilibration factor "3" at 5% buffer B. Trap column washing and equilibration are conducted with enabled fast wash and equilibration together with zebra wash (2 wash cycles, automatic equilibration factor). The Orbitrap Eclipse mass spectrometer was run with an internal real-time mass calibration using a user-defined lock mass (positive, m/z = 445.12003) and operated in data-independent acquisition mode. The full MS scans were collected in the Orbitrap at a resolution of 60000 and an AGC target of 4e5 with a maximum injection time of 100 ms in a scan range of 400-1000 m/z. MS2 spectra were collected at a resolution of 15,000 and an AGC target of 1e6 with a maximum injection time of 40 ms. The isolation in the quadrupole was conducted with windows of 10 m/z with an overlap of 1 m/z in a scan range of 145-1450 m/z. Higher-energy collision-induced dissociation (HCD) with a normalized collision energy of 30% was used to generate the fragments that were detected in the Orbitrap. Data acquisition was performed using Thermo Scientific Foundation software (version 3.1sp9) and Xcalibur (version 4.6).

Data Analysis. Acquired raw files were converted into mzML format using the MSConvert tool (version: 3.0.21193-ccb3e0136) of the ProteoWizard software4 (version: 3.0.21193 64-bit). MS data were processed using DIA-NN (version 1.8.1) in library-free mode.[8] The UniProt reference proteome for *E. coli* K12 (taxon identifier: 83333, downloaded on 22.01.2024) was used for library generation. The settings for precursor ion generation included the FASTA digest for library-free search and library creation and using deep-learning algorithms to predict spectra, retention times (RTs), and ion mobilities (IMs). Trypsin/P was specified as the protease, allowing for a maximum of two missed cleavages. The method involved excising N-terminal methionine and applying carbamidomethylation to cysteines as a fixed modification with no variable modifications. The peptide lengths were set to range from 7 to 30 residues, and the precursor charges were selected to be between 2 and 4. The precursor m/z range was established from 300 to 1,800, and the fragment m/z range was set from 200 to 1,800. The precursor false discovery rate (FDR) was established at 0.01. Settings for mass accuracy, MS1 accuracy, and scan window were all configured to 0. Features such as isotopologues, match-between-runs (MBR), and removal of likely interferences were activated. The neural network classifier was operated in single-pass mode, conducting protein inference at the gene level with heuristic protein inference enabled (--relaxed-prot-inf). Quantification was carried out

using the robust LC (high precision) strategy. Cross-run normalization was dependent on RT, smart profiling was employed for library generation, and optimal settings were used for both speed and RAM usage. After DIA-NN analysis, LFQ quantities for all protein groups were analyzed using Perseus software (version 2.0.9.0).[9] After a log_2 transformation of the LFQ intensities, protein groups with less than three valid values in at least one group were filtered out, and a two-sample Student's t-test with permutation-based multiple testing correction (FDR = 0.05) was used to determine the fold change values and statistical significance. Results tables were exported, and graphs were generated using *GraphPad* Prism (version 10.01).

In vitro translation inhibition assay

To assess translation inhibition, an *E. coli* cell-free lysate-based translation system (RTS100 E. coli HY; biotechrabbit) was used to express the reporter enzyme firefly luciferase, as previously described for other translation inhibiting antibiotics.[10]

Briefly, a 5 µL reaction mix prepared according to manufacturer protocol is mixed with 1 µL tested compound at the indicated concentrations. The reactions were incubated for 30 min at 32°C at 600 rpm and then stopped by adding 3 µL of Kanamycin (50 µg/µL) to each reaction tube, subsequently cooled down on ice. The stopped reactions were transferred to a black 96-well chimney flat-bottom microtiter plate and mixed with 40 µL of Fluc substrate (Promega). Fluorescence was measured using a TECAN infinite 200Pro plate reader. Samples were normalized relative to reactions without antibiotic.

PCR & Cloning

PCR. Genomic DNA of *E. coli* K12 was isolated by using the peqGOLD Bacterial DNA kit (*VWR*) according to the manufacturer's protocol. Recombinant *fliC* (UniProt ID: P04949) was cloned into expression vector pET301CT-DEST™ (*Invitrogen*) by using Gateway® Cloning (*Invitrogen*). The target gene was amplified by PCR with the Phusion High-Fidelity DNA Polymerase (*New England Biolabs*) according to the manufacturer's instructions. The used primers (**Table SS5**) and PCR conditions (**Table S6** & **Table S7**) are listed in the table below. PCR products were verified by a 1% analytical agarose gel and purified by preparative 1% agarose gel followed by DNA extraction using a gel extraction kit (Omega) according to the manufacturer's protocol.

Table S5: List of Primers.

Primer	Sequence
**FliC fwd**	GGGGACAAGTTTGTACAAAAAAGCAGGCTTTGAAGGAGATAGAACCATGGCACAAGTCATTAATACCAACAG
**FliC rev**	GGGGACCACTTTGTACAAGAAAGCTGGGTGGCCCTGAAAATAAAGATTCTCACCCTGCAGCAGAGACAG

Table S6: Conditions used for the PCR reaction.

Reagents	Volume [µL]
**5× Phusion GC buffer**	10
**dNTPs (10 mM)**	1
**Forward Primer (10 µM)**	2.5
**Reverse Primer (10 µM)**	2.5
**Genomic DNA (50 ng/µL)**	2.5
**DMSO**	1.5
**Phusion DNA Polymerase**	0.5
**ddH_2O**	29.5

Table S7: Temperature program used for the thermocycler of the PCR reaction.

Step	Temperature [°C]	Time [s]	Cycles
**Initial denaturation**	98	20	1
**Denaturation**	98	20	
**Annealing**	66.2	30	30
**Extension**	72	25	
**Final extension**	72	600	1
**Hold**	4	∞	-

Cloning. Gateway® cloning (*Invitrogen*) with pDONR™201$_{Kan}$ (*Invitrogen*) as donor vector and pET301CT-DEST™$_{Amp}$ (Invitrogen) as the destination vector was performed according to adopted protocols of Invitrogen.[102] Final expression vectors were confirmed by Sanger sequencing (*Azenta*) and were transformed in *E. coli* BL21 (DE3).

FliC Protein production & purification

LB medium (4×1L) supplemented with 0.1 mg/mL ampicillin (stock in ethanol/ddH_2O = 1:1, c_{stock} = 100 mg /mL) was inoculated with pre- cultures (1:100) of the *fliC* vector containing *E. coli* BL21 (DE3) strain, incubated (37 °C, 200 rpm) and overexpression was induced at OD_{600} = 0.6-0.8 by addition of iso-propyl-1-thio-β-galactopyranoside (IPTG, stock in ddH_2O, c_{stock} = 1 M) to a final concentration of 1 mM. *fliC* was overexpressed for 2h (37 °C, 200 rpm) followed by harvesting (6000 g, 10 min, 4 °C) the cells and washing the cell pellet with cold PBS. Cell pellets were reconstituted in lysis buffer (20 mM Tris–HCl, 200 mM NaCl, pH = 7.6) and lysed by sonication (2x (7 min 30% intensity, 2 min 80% intensity) on ice, Sonopuls HD 2070 ultrasonic rod, *Bandelin electronic* GmbH). Lysozyme and DNAse I were added to the lysate and

incubated for 30 min on ice. The lysate was cleared by centrifugation (18000 rpm, 30 min, 4 °C), and the supernatant was filtered through a syringe filter (0.4 µm). FliC was purified using an Aekta Pure Protein Purification System (*Cytiva*). Lysate was loaded onto an equilibrated 5 mL HisTrapHP column (*Cytiva*) at a flow rate of 2 mL/min. The column was washed with 5 column volumes (CV) of lysis buffer (20 mM Tris–HCl, 200 mM NaCl, pH = 7.6) and then washed with another 7 CVs of wash buffer (20 mM Tris–HCl, 200 mM NaCl, 50 mM Imidazole, pH = 7.6). The bound proteins were eluted by 7 CVs of elution buffer (20 mM Tris–HCl, 200 mM NaCl, 250 mM Imidazole, pH = 7.6). The elution fraction was transferred into a dialysis membrane (3 kDa cutoff) and dialyzed (4 °C, 3h) in 1 L lysis buffer. The lysis buffer was exchanged to 1 L lysis buffer supplemented with EDTA (1 mM final concentration), and TEV protease was added to the protein to cleave off the His-tag. After TEV-cleavage, the protein was loaded in two runs on a Superdex 75 (*Cytiva*) and eluted using lysis buffer (20 mM Tris–HCl, 200 mM NaCl, pH = 7.6). The protein fractions were checked by SDS-PAGE and intact protein mass spectrometry (IPMS). Protein aliquots were stored at -80 °C.

Gel-based Fluorescence labeling of recombinant FliC

Recombinant FliC was diluted in PBS to a final concentration of 6 µM in 50 µL PBS. For the heat-denatured sample a final concentration of 0.4% SDS was added to the buffer and the sample was heated to 95 °C for 5 minutes, before cooled again to room temperature. 100× stock concentrations of Ian-P-1 or DMSO (1%) were added to the solution and incubated (600 rpm, rt) for 1h. 6 µL of click mix (1 µL 5 mM rhodamine azide in DMSO, 1 µL 50 mM $CuSO_4$ in H_2O, 1 µL 50 mM Tris(2-carboxyethyl)phosphine (TCEP) in H_2O and 3 µL 1.67 mM tris(benzyltriazoylmethyl)amine (TBTA) in 80% *t*-BuOH and 20% DMSO) were added per sample and incubated (500 rpm, rt) for 1.5h. 50 µL of 2× Laemmli buffer was added, the sample was vortexed and loaded on a 12.5% SDS-PAGE. The fluorescence was recorded in a Fujifilm Las-4000 Luminescent Image Analyzer with a Fujinon VRF43LMD3 and a 75DF20 filter.

Microscopical Motility tracking of *E. coli* K12

A pre-culture of *E. coli* K12 was used to inoculate (OD_{600} = 0.05) fresh LB medium (5 mL) and bacteria were grown (37 °C, 200 rpm) to an OD_{600} = 0.8-1. 99 µL of the bacterial suspension was transferred to a microcentrifuge tube, and 1 µL of Ian-MPD-1 (625 µM stock concentration) was added. The bacteria were incubated (37 °C, 500 rpm) on an Eppendorf shaker for 30 min, 10 µL transferred to a microscopy slide, and covered with a coverslip (LifterSlip™, *EMS*, catalog no. E72186-36). The bacterial motility was tracked at 37 °C for 10 s (10 fps) on a Leica DMi8 inverted microscope equippedwith a Leica DFC365 FX camera (Wetzlar, Germany) and 40× objective lens. Per slide, three different positions were tracked. The measurement was performed in three independent replicates.

For further processing a custom python script was used (code available at https://github.com/MIsselstein/E.Coli_Tracker).[11–13] First, the pictures were defined as binary images and the 99 pictures of a 10 s tracking experiment were put into one movie. For cell detection, the search range (defined as the maximum distance features

can move between frames) was set to 20 and the memory (defined as the maximum number of frames during which a feature can vanish, then reappear nearby, and be considered the same particle) to 5. The magnification was set to 40 and the pixel size to 6.45. The detected trajectories were exported as a movie and as an Excel file for further processing.

Transmission electron microscopy (TEM)

A pre-culture of *E. coli* K12 was used to inoculate (OD_{600} = 0.05) fresh LB medium (5 mL) per condition, and the bacteria were grown (37 °C, 200 rpm) to an OD_{600} = 0.6. The cells were harvested (6000 g, 10 min, 4 °C) and resuspended in M9 minimal media in the same volume to get a theoretical OD_{600} = 0.6. DMSO (1%) or **Ian-MPD-1** (25 µM) was added, and the bacteria were treated for 1h (37 °C, 200 rpm). Bacterial culture was chemically fixed with glutaraldehyde 2.5%. Copper grids for TEM were coated with a carbon film of 10 nm thickness and additionally hydrophilized. 10 µL of bacterial culture was dropped on the coated side of the grid for 2 min and followed by plotting. For the staining, the carbon-coated side of the grids was washed twice with sterile water and stained for 15 seconds with 1% Uranyl acetate. Between all washing and staining steps, plotting with filter paper was repeated to remove the liquids. Images of the negatively stained samples were taken with the transmission electron microscope (EM912, Zeiss Microscopy GMBH) at 80kV.

TEM pictures were checked manually for the presence of flagella, and statistical analysis of the flagellation status was done by analyzing multiple bacterial cells ($n > 90$) per condition.

Invasion assay

HeLa cells were seeded in a density of 10000 cells per well in a 12-well plate. The human cells were grown (37 °C, 5% CO_2) overnight. Then, the medium was aspirated and the cells were washed once with PBS. A pre-culture of *E. coli* CFT073 was diluted to 2.5 million cells per mL in DMEM medium (without FBS) and 100 µL per well of the bacterial suspension was added to the human cells. 100 µL of Ian-MPD-1 (100 µM, 50 µM or 25 µM final concentration, 1% DMSO) or DMSO (1%) in DMEM medium (without FBS) were added to the wells. The plate was centrifuged (300 g, 3 min) and the human cells were infected for 2h (37 °C, 5% CO_2). The medium was aspirated and the human cells were washed once with PBS. The external bacteria were killed by incubating the human cells with 300 µL DMEM (with FBS) supplemented with 25 µg/mL Gentamicin sulfate for 1h. The medium was aspirated and the human cells washed three times with PBS. Human cells were lysed by the addition of 150 µL Triton X-100 in PBS (0.5%) and serial dilutions of the lysate were plated on LB agar plates. Each experiment was performed in two technical replicates per experiment and repeated twice in independent biological replicates.

Chemical Synthesis

General

All chemicals and reagents were commercially available and used without further purification. Glassware for reactions under an inert atmosphere was purged three times with argon. Dry solvents were purchased and transferred by disposable syringes and added to the reaction flask under argon counterflow.

Reaction control

Reactions were monitored by LC-MS (MSQ Plus, *Thermo Fisher Scientific Inc.*, coupled to a *Dionex* UltiMate 3000 HPLC) or TLC silica gel 60 F254 plates (Merck) with visualization by UV-light (λ = 254 and 366 nm) or staining with appropriate solutions ($KMnO_4$ stain: 1.50 g $KMnO_4$, 10.0 g K_2CO_3, 1.25 mL NaOH 10% (w/v), 200 mL ddH_2O; Ninhydrin stain: 0.3 g ninhydrin, 100 mL *n*-butanol, 3 mL acetic acid). Crude products were purified by silica column chromatography with silica gel 60 (particle size = 40 – 63 µm, *VWR*).

Chromatography

If specified, purification was carried out using reversed-phase high-performance liquid chromatography (HPLC). For analytical scale, a *Waters* 2695 separation module coupled with a *Waters* PDA 2996 and a *Waters* XBridge C18 column (3.5 µm, 4.6 × 100 mm, flow: 1.2 mL/min) was used. For semi-preparative scale a *Waters* 2545 quaternary gradient module coupled to a *Waters* PDA 2998 was used. Depending on the crude product scale different reversed-phase columns were used: P1: *YMC* Triat C18 (3.5 µm, 10 × 250 mm, flow rate: 10 mL/min), P2: *Waters* XBridge™ C18 (5.0 µm, 30 × 150 mm, flow: 50 mL/min). The specific gradient used for purification is provided individually for each compound.

Gradient 1

Time [min]	% H_2O + 0.1% TFA	% Acetonitrile + 0.1% TFA	% H_2O	% Acetonitrile
0			98	2
12			70	30
14			2	98
15			2	98
16			98	2
18			98	2

Gradient 2

Time [min]	% H_2O + 0.1% TFA	% Acetonitrile + 0.1% TFA	% H_2O	% Acetonitrile
0	98	2		

14	50	50		
15	2	98		
17	2	98		
18	98	2		
20	98	2		

Gradient 3

Time [min]	% H_2O + 0.1% TFA	% Acetonitrile + 0.1% TFA	% H_2O	% Acetonitrile
0	98	2		
25	55	45		
27	2	98		
28	2	98		
30	98	2		
32	98	2		

NMR spectroscopy

NMR spectra were recorded at 298 K on a *Bruker* AVHD-400, AVHD500 or AV-II-500 equipped with a cryo probe head. Chemical shifts are reported in parts per million (ppm). Spectra were referenced to residual proton and carbon signals of the deuterated solvent: CD_3OD (δ (^1H) = 3.31 ppm, δ (^{13}C) = 49.0 ppm), $CDCl_3$ (δ (^1H) = 7.26 ppm, δ (^{13}C) = 77.16 ppm), $(CD_3)_2SO$ (δ (^1H) = 2.50 ppm, δ (^{13}C) = 39.52 ppm), $(CD_3)_2CO$ (δ (^1H) = 2.05 ppm, δ (^{13}C) = 29.84 ppm). NMR coupling patterns were abbreviated as follows: s (singlet, d (doublet), t (triplet), q (quartet), m (multiplet), br (broad). The coupling constants J are reported in Hertz.

High-resolution mass spectrometry

High-resolution mass spectrometry (HRMS) was performed on a LTQ-FT Ultra from *Thermo Fisher Scientific* (ESI). EI results were obtained from a *Thermo Fisher Scientific* DFS-HRMS spectrometer.

3,3'-(hexane-1,6-diylbis(azanediyl))dipropanenitrile (DA-1a)

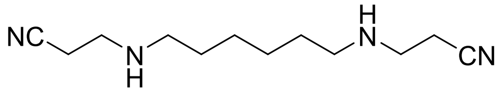

DA-1a

Chemical Formula: $C_{12}H_{22}N_4$
Molecular Weight: 222.33 g/mol

The reaction was performed in an inert atmosphere using argon according to a published procedure.[14]

Hexane-1,6-diamine (1.37 g, 11.8 mmol, 1.0 eq) was dissolved in anhydrous methanol (5 mL) and the solution was cooled to 0 °C. A solution of acrylonitrile (1.25 g, 23.6 mmol, 2.0 eq) in anhydrous methanol (25 mL) was added dropwise to the reaction mixture. The reaction mixture was allowed to warm up to room temperature, covered with aluminum foil, and stirred overnight. The excess solvent was removed under reduced pressure and the crude product was purified *via* HPLC (P2, gradient 1) to afford the desired product (1.56 g, 7.02 mmol, *60%*) as a colorless oil.

^{1}H-NMR (400 MHz, CD_3OD): δ [ppm] = 2.87 (t, J = 6.9 HZ, 4H), 2.61 (td, J = 7.1 Hz, J = 3.5 Hz, 8H), 1.45 (m, 8H).

^{13}C-NMR (101 MHz, $(CD_3)_2SO$): δ [ppm] = 119.7, 48.2, 44.3, 28.7, 26.4,17.2.

HRMS (ESI): *m/z* calcd. for $[C_{12}H_{22}N_4+H]^+$: 223.1917, found: 223.1912.

Di-*tert*-butyl hexane-1,6-diylbis((2-cyanoethyl)carbamate) (DA-1b)

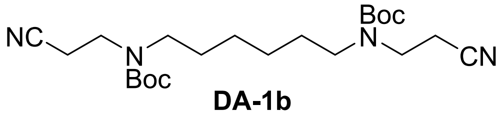

DA-1b

Chemical Formula: $C_{22}H_{38}N_4O_4$
Molecular Weight: 422.57 g/mol

The reaction was performed in an inert atmosphere using argon according to a published procedure.[14]

3,3'-(hexane-1,6-diylbis(azanediyl))dipropanenitrile (617 mg, 2.78 mmol, 1.0 eq) was dissolved in anhydrous methanol (12 mL) and the solution was cooled to 0 °C. A solution of di-*tert*-butylpyrocarbonate (1.82 g, 8.34 mmol, 3.0 eq) in anhydrous methanol (13 mL) was added dropwise to the reaction mixture and the reaction mixture was allowed to warm up to room temperature. After a reaction time of 3 h, the solvent was removed under reduced pressure, and the crude product was purified via column chromatography (pentane:ethyl acetate = 70:30). The desired product (1.14 g, 2.69 mmol, 97%) was obtained as a colorless, viscous oil.

TLC: R_f = 0.31 (pentane:ethyl acetate = 70:30)

^{1}H-NMR (400 MHz, $CDCl_3$): δ [ppm] = 3.44 (t, J = 6.7 Hz, 4H), 3.24 (t, J = 7.5 Hz, 4H), 2.59 (br, 4H), 1.46 (m, 22H), 1.29 (p, J = 3.4 Hz, 4H).

^{13}C-NMR (101 MHz, $CDCl_3$): δ [ppm] = 155.4, 118.5, 80.4, 48.7, 47.7, 44.0, 43.6, 28.9, 28.4, 26.6, 17.6, 17.1

HRMS (ESI): *m/z* calcd. for $[C_{12}H_{38}N_4O_4+H]^+$: 423.2966, found: 423.2956.

Di-*tert*-butyl hexane-1,6-diylbis((3-aminopropyl)carbamate) (DA-1)

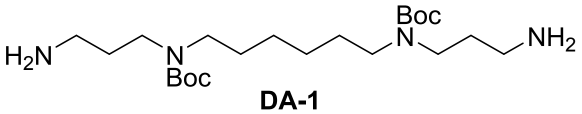

DA-1

Chemical Formula: $C_{22}H_{46}N_4O_4$
Molecular Weight: 430.63 g/mol

The reaction was performed according to a published procedure in a Schlenk flask.[14]

To a solution of di-*tert*-butyl hexane-1,6-diylbis((2-cyanoethyl)carbamate) (1.07 g, 2.54 mmol, 1.0 eq) in a mixture of methanol/water 5:1 (21.6 mL) were added a catalytical amount of RANEY-Nickel® (50% aqueous suspension), Pd/C (10 wt%, 135 mg, 0.13 mmol, 0.05 eq) as well as lithium hydroxide monohydrate (74.2 mg, 3.1 mmol, 1.2 eq) at room temperature. The reaction flask was carefully evacuated and purged several times with hydrogen gas using a balloon filled with hydrogen. After purging, the balloon needle was inserted into the reaction mixture to aerate the solution, as indicated by visible bubbling. To balance the internal pressure, an additional cannula was inserted through the septum alongside the balloon. The reaction mixture was stirred at room temperature for 48 h. During reaction periods overnight, the additional cannula was removed from the septum and the cannula of the hydrogen balloon was removed from the solution. After completion of the reaction, the mixture was filtered through a pad of celite® in a fritted Büchner funnel, and the reaction flask and the filter were repeatedly washed with methanol. The solvent was removed under reduced pressure to reveal the desired product (920 mg, 2.14 mmol, *84%*) as a grey, viscous oil.

^{1}H-NMR (400 MHz, CD_3OD): δ [ppm] = 3.27 (m, 4H), 3.19 (t, 4H, *J* = 7.5 Hz), 2.61 (m, 4H), 1.67 (m, 4H), 1.56 (m, 4H), 1.46 (s, 18H), 1.31 (m, 4H).

^{13}C-NMR (101 MHz, CD_3OD): δ [ppm] = 157.6, 157.3, 80.7, 48.0, 45.9, 45.0, 40.0, 39.5, 32.9, 32.1, 29.6, 29.1, 28.7, 28.7, 27.7.

HRMS (ESI): *m/z* calcd. for $[C_{22}H_{46}N_4O_4+H]^+$: 431.3592, found: 431.3580.

These data are in accordance with previous literature reports.[14]

Di-tert-butyl butane-1,4-diylbis((2-cyanoethyl)carbamate) (DA-2a)

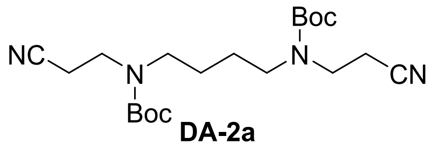

DA-2a

Chemical Formula: $C_{20}H_{34}N_4O_4$
Molecular Weight: 394.52 g/mol

The reactions were performed in an inert atmosphere using argon according to a published procedure.[14]

1,4-Diaminobutane (0.91 g, 10.1 mmol, 1.0 eq) was dissolved in anhydrous methanol (8 mL) and cooled to 0 °C. In a separate flask acrylonitrile (1.34 mL, 20.1 mmol, 2.0 eq) was dissolved in anhydrous methanol (23 mL) and the mixture was added dropwise to the reaction mixture. After that, the reaction mixture was stirred at 0 °C for 1 h, then allowed to warm up to room temperature, covered with aluminum foil and stirred for 2 h. The solvent was removed *in vacuo* to afford the intermediate nitrile. The intermediate was used for subsequent experiments without further purification. Intermediate nitrile (0.95 g, 4.90 mmol, 1.0 eq) was dissolved in anhydrous methanol (22 mL) and the solution was cooled to 0 °C. Then, triethylamine (1.51 mL, 10.8 mmol, 2.2 eq) was added to the mixture. In another flask di-tert-butyl pyrocarbonate (2.78 mL, 12.7 mmol, 2.6 eq) was dissolved in anhydrous methanol (23 mL) and the solution was added dropwise to the reaction mixture. After that, the reaction mixture was stirred at 0 °C for 10 min and then allowed to warm up to room temperature. After 20 h, the solvent was removed *in vacuo*. The residue was purified *via* column chromatography (pentane:ethyl acetate 100:0 → 70:30) to afford the desired product as a colorless solid (1.42 g, 3.60 mmol, *36%*).

TLC: R_f = 0.53 (pentane:ethyl acetate = 40:60)

^{1}H-NMR (500 MHz, $CDCl_3$): δ [ppm] = 3.46 (t, *J* = 6.7 Hz, 4H), 3.29 (t, *J* = 6.7 Hz, 4H), 2.67 – 2.53 (m, 4H), 1.54 – 1.51 (m, 4H), 1.47 (s, 18H).

Di-tert-butyl butane-1,4-diylbis((3-aminopropyl)carbamate)

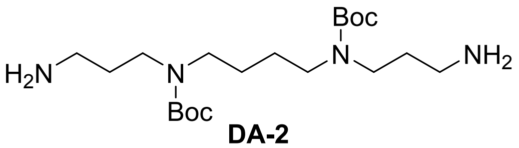

DA-2

Chemical Formula: $C_{20}H_{42}N_4O_4$
Molecular Weight: 402.58 g/mol

The reaction was performed according to a published procedure in a Schlenk flask.[14]

Di-tert-butyl butane-1,4-diylbis((2-cyanoethyl)carbamate) (0.71 g, 1.80 mmol, 1.0 eq) was dissolved in a mixture of methanol:water 4:1 (15 mL). Then, Pd/C (5 wt%, 0.19 g, 0.09 mmol, 0.1 eq) and lithium hydroxide (50.0 mg, 2.20 mmol, 1.2 eq) were added to

the mixture. After that, catalytical amounts of RANEY-Nickel® (50% aqueous suspension) were added to the reaction mixture. The reaction flask was carefully evacuated and purged several times with hydrogen gas using a balloon filled with hydrogen. After purging, the balloon needle was inserted into the reaction mixture to aerate the solution, as indicated by visible bubbling. To balance the internal pressure, an additional cannula was inserted through the septum alongside the balloon. The reaction mixture was stirred at room temperature for 22 h. During the reaction period overnight, the additional cannula was removed from the septum and the cannula of the hydrogen balloon was removed from the solution. Subsequently, the mixture was filtered through a celite® plug, washed with methanol several times and the solvent was removed *in vacuo* to afford the desired product (quant. yield) as a grey liquid.

^{1}H-NMR (400 MHz, CD_3OD): δ [ppm] = 3.30 – 3.23 (m, 4H), 3.22 (t, *J* = 6.9 Hz, 4H), 2.61 (t, *J* = 6.8 Hz, 4H), 1.73 – 1.63 (m, 4H), 1.57 – 1.49 (m, 4H), 1.46 (s, 18H).

^{13}C-NMR (101 MHz, CD_3OD): δ [ppm] = 157.5, 80.9, 48.0, 45.2, 39.6, 33.0, 32.3, 28.8.

These data are in accordance with previous literature reports.[15]

2,4-dibromo-5-methoxybenzaldehyde (CA-1a)

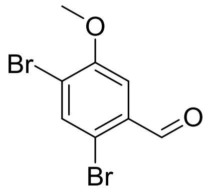

CA-1a

Chemical Formula: $C_8H_6Br_2O_2$
Molecular Weight: 293.94 g/mol

The reaction was performed in an inert atmosphere using argon according to a published procedure.[16]

To a solution of 2,4-dibromo-5-hydroxybenzaldehyde (840 mg, 3.00 mmol, 1.0 eq) in anhydrous dimethylformamide (25 mL) was added potassium carbonate (750 mg, 5.43 mmol, 1.8 eq), after which the mixture turned yellow. After the addition of iodomethane (705 mg, 4.96 mmol, 1.7 eq), the reaction was left to stir at room temperature for 5 h. Water (30 mL) was added to the reaction mixture and the aqueous phase was extracted with diethyl ether (3 × 20 mL). The combined organic layers were washed with LiCl solution (5 wt%, 2 × 15 mL), brine (1 × 20 mL), dried over $NaSO_4$, filtered, and concentrated *in vacuo*. The desired product (817 mg, 2.78 mmol, *93%*) was obtained as a white, flaky solid.

^{1}H-NMR (400 MHz, $CDCl_3$): δ [ppm] = 10.26 (s, 1H), 7.86 (s, 1H), 7.41 (s, 1H), 3.94 (s, 3H).

^{13}C-NMR (101 MHz, $CDCl_3$): δ [ppm] = 191.1, 156.1, 137.7, 133.2, 120.1, 118.0, 111.2, 56.8.

HRMS (EI): *m/z* calcd. for $[C_8H_6Br_2O_2\cdot]^+$: 293.8709, found: 293.8713.

These data are in accordance with previous literature reports.[16]

Ethyl (*E*)-3-(2,4-dibromo-5-methoxyphenyl)acrylate (CA-1b)

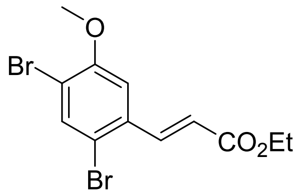

CA-1b

Chemical Formula: $C_{12}H_{12}Br_2O_3$
Molecular Weight: 364.03 g/mol

The reaction was performed in an inert atmosphere using argon according to a published procedure.[17]

To a mixture of LiCl (50.4 mg, 1.19 mmol, 1.8 eq) in anhydrous tetrahydrofuran (3.2 mL) was added triethyl phosphonoacetate (0.24 mL, 1.19 mmol, 1.8 eq) and stirred at room temperature for 15 minutes. Then 1,8-Diazabicyclo[5.4.0]undec-7-en (0.18 mL, 1.19 mmol, 1.8 eq) was added and stirred at room temperature for an additional 5 minutes, followed by the addition of 2,4-dibromo-5-methoxybenzaldehyde (194 mg, 0.66 mmol, 1.0 eq). After complete consumption of the starting material (TLC control), the solvent was removed under reduced pressure and the crude product was taken in saturated ammonium chloride solution (20 mL) and extracted with ethyl acetate (3 × 15 mL). The combined organic layers were washed with brine (1 × 10 mL), dried over $MgSO_4$, filtered, and concentrated *in vacuo*. The crude product was then purified by column chromatography (pentane:ethyl acetate = 100:0 → 97.5:2.5) to yield the desired product (179 mg, 0.49 mmol, *75%*) as a white, flaky solid.

TLC: R_f = 0.46 (pentane:ethyl acetate = 90:10)

^{1}H-NMR (400 MHz, $CDCl_3$): δ [ppm] = = 7.94 (d, 1H, *J* = 15.9 Hz), 7.78 (s, 1H), 7.06 (s, 1H), 6.39 (d, 1H, *J* = 15.9 Hz), 4.29 (q, 2H, *J* = 7.1 Hz), 3.92 (s, 3H), 1.35 (t, 3H, J = 7.1 Hz).

HRMS (ESI): *m/z* calcd. for $[C_{12}H_{12}Br_2O_3+H]^+$: 362.9225, found: 362.9222.

These data are in accordance with previous literature reports.[14]

(*E*)-3-(2,4-dibromo-5-methoxyphenyl)acrylic acid (CA-1c)

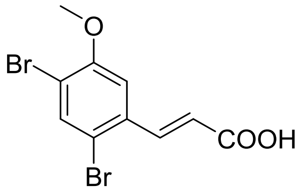

CA-1c

Chemical Formula: $C_{10}H_8Br_2O_3$
Molecular Weight: 335.98 g/mol

The reaction was performed according to a published procedure.[14]

To a cooled solution (0 °C) of Ethyl (*E*)-3-(2,4-dibromo-5-methoxyphenyl)acrylate (179 mg, 0.49 mmol, 1.0 eq) in a mixture of tetrahydrofuran/water 5:1 (6 mL) was added lithium hydroxide monohydrate (31 mg, 0.74 mmol, 1.5 eq). The reaction mixture was then allowed to warm up to room temperature and stirred overnight. After complete consumption of the starting materials (TLC control), the excess solvent was removed under reduced pressure. The residue was taken up in water (1 mL) and acidified using a 2M HCl solution to a pH of 2-3. The aqueous layer was extracted with ethyl acetate (4 × 5 mL). The combined organic phases were washed with brine (1 × 10 mL), dried over $MgSO_4$, filtered, and concentrated *in vacuo* to yield the desired product (154 mg, 0.46 mmol, *93%*) as a white solid.

TLC: R_f = 0.26 (pentane:ethyl acetate = 60:40 + 1% acetic acid)

^{1}H-NMR (400 MHz, $(CD_3)_2CO$): δ [ppm] = 7.92 (d, 1H, *J* = 15.9 Hz), 7.87 (s, 1H), 7.56 (s, 1H), 6.68 (d, 1H, *J* = 15.9 Hz), 4.03 (s, 3H).

^{13}C-NMR (101 MHz, $(CD_3)_2CO$): δ [ppm] = 167.1, 155.3, 140.6, 135.9, 133.9, 123.3, 115.1, 113.7, 111.3, 56.8.

HRMS (ESI): *m/z* calcd. for $[C_{10}H_8Br_2O_3+H]^+$: 332.8767, found: 332.8767.

These data are in accordance with previous literature reports.[14]

2,5-dioxopyrrolidin-1-yl-(*E*)-3-(2,4-dibromo-5-methoxyphenyl)acrylate (CA-1)

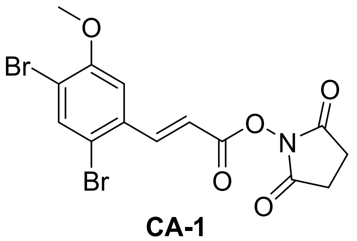

CA-1

Chemical Formula: $C_{14}H_{11}Br_2NO_5$
Molecular Weight: 433.05 g/mol

The reaction was performed in an inert atmosphere using argon according to a published procedure.[18]

To a solution of (*E*)-3-(2,4-dibromo-5-methoxyphenyl)acrylic acid (163 mg, 0.49 mmol, 1.0 eq) in a mixture of anhydrous tetrahydrofuran:dimethylformamide 9:1 (4.5 mL) was added EDC·HCl (112 mg, 0.58 mmol, 1.2 eq) and N-hydroxysuccinimide (73 mg, 0.63 mmol, 1.3 eq) at room temperature and stirred until full conversion (TLC control). The reaction was taken up in water (8 mL) and extracted with ethyl acetate (3 × 5 mL). The combined organic layers were washed with LiCl solution (5 wt%, 3 × 5 mL), brine (1 × 8 mL), dried over $MgSO_4$, filtered, and concentrated *in vacuo* to yield the desired product (190 mg, 0.42 mmol, *87%*) as a viscous, colorless oil.

^{1}H-NMR (400 MHz, $CDCl_3$): δ [ppm] = 8.20 (d, 1H, *J* = 16.0 Hz), 7.82 (s, 1H), 7.10 (s, 1H), 6.57 (d, 1H, *J* = 16.0 Hz), 3.94 (s, 3H), 2.90 (m, 4H).

^{13}C-NMR (101 MHz, $CDCl_3$): δ [ppm] = 169.2, 161.4, 155.8, 147.7, 137.4, 114.8, 110.1, 56.7, 25.8.

HRMS (ESI): *m/z* calcd. for $[C_{14}H_{11}Br_2NO_5+H]^+$: 431.9076, found: 431.9075.

2,4-dibromo-5-(prop-2-yn-1-yloxy)benzaldehyde (CA-2a)

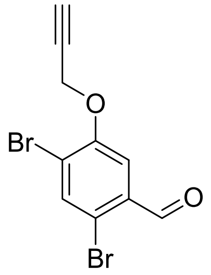

CA-2a

Chemical Formula: $C_{10}H_6Br_2O_2$
Molecular Weight: 317.96 g/mol

The reaction was performed in an inert atmosphere using argon according to a published procedure.[19]

To a solution of 2,4-dibromo-5-hydroxybenzaldehyde (840 mg, 3.00 mmol, 1.0 eq) in anhydrous dimethylformamide (25 mL) was added potassium carbonate (750 mg, 5.43 mmol, 1.8 eq), after which the mixture turned yellow. After the addition of propargyl bromide (607 mg, 4.95 mmol, 1.7 eq) the reaction was left to stir at room temperature for 21 hours, during which a brown coloration of the solution occurred. The reaction was stopped, taken up in water (40 mL) and extracted with diethyl ether (5 × 30 mL). The combined organic layers were washed with LiCl solution (5 wt%, 3 × 20 mL), brine (1 × 30 mL), dried over Na_2SO_4, filtered, and concentrated *in vacuo*. The desired product (817 mg, 2.78 mmol, *87%*) was obtained as an orange-brown solid.

^{1}H-NMR (400 MHz, $CDCl_3$): δ [ppm] = 10.26 (s, 1H), 7.88 (s, 1H), 7.56 (s, 1H), 4.84 (d, 2H, J = 2.4 Hz), 2.58 (t, 1H, *J* = 2.4 Hz).

^{13}C-NMR (101 MHz, $CDCl_3$): δ [ppm] = 190.8, 154.1, 138.0, 133.2, 120.6, 118.8, 113.1, 77.3, 77.2, 57.2.

HRMS (EI): *m/z* calcd. for $[C_{10}H_6Br_2O_2\cdot]^+$: 317.8709, found: 317.8704.

Ethyl (*E*)-3-(2,4-dibromo-5-(prop-2-yn-1-yloxy)phenyl)acrylate (CA-2b)

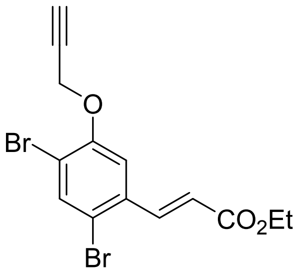

CA-2b

Chemical Formula: $C_{14}H_{12}Br_2O_3$
Molecular Weight: 388.06 g/mol

The reaction was performed in an inert atmosphere using argon according to a published procedure.[17]

To a mixture of LiCl (70.3 mg, 1.66 mmol, 1.3 eq) in anhydrous tetrahydrofuran (3.2 mL) was added triethyl phosphonoacetate (0.34 mL, 1.7 mmol, 1.3 eq) and stirred at room temperature for 15 minutes. Then 1,8-Diazabicyclo[5.4.0]undec-7-en (0.25 mL, 1.66 mmol, 1.3 eq) was added and stirred at room temperature for an additional 5 minutes, followed by the addition of 2,4-dibromo-5-(prop-2-yn-1-yloxy)benzaldehyde (405 mg, 1.27 mmol, 1.0 eq). After complete consumption of the starting material (TLC control), the solvent was removed under reduced pressure and the crude product was taken in saturated ammonium chloride solution (20 mL) and extracted with ethyl acetate (3 × 15 mL). The combined organic layers were washed with brine (1 × 10 mL), dried over $MgSO_4$, filtered, and concentrated *in vacuo*. The crude product was then

purified by column chromatography (pentane:ethyl acetate = 100:0 → 97.5:2.5) to yield the desired product (400 mg, 1.03 mmol, *81%*) as a white, flaky solid.

TLC: R_f = 0.25 (pentane:ethyl acetate = 95:5)

^{1}H-NMR (400 MHz, $CDCl_3$): δ [ppm] = = 7.94 (d, 1H, *J* = 16.0 Hz), 7.81 (s, 1H), 7.26 (s, 1H), 6.38 (d, 1H, *J* = 16.0 Hz), 4.80 (d, 2H, *J* = 2.4 Hz), 4.29 (q, 2H, *J* = 7.1 Hz), 2.60 (t, 1H, *J* = 2.4 Hz), 1.36 (t, 3H, *J* = 7.1 Hz).

^{13}C-NMR (101 MHz, $CDCl_3$): δ [ppm] = 166.8, 154.7, 142.2, 137.7, 135.3, 123.2, 117.2, 115.4, 113.4, 78.4, 77.9, 61.7, 58.1, 14.5.

HRMS (ESI): *m/z* calcd. for $[C_{14}H_{12}Br_2O_3+H]^+$: 386.9225, found: 386.9221.

(*E*)-3-(2,4-dibromo-5-(prop-2-yn-1-yloxy)phenyl)acrylic acid (CA-2c)

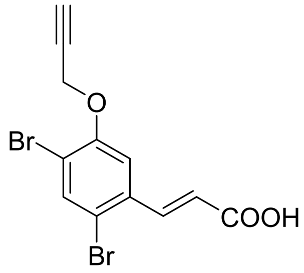

CA-2c

Chemical Formula: $C_{12}H_8Br_2O_3$
Molecular Weight: 360.00 g/mol

The reaction was performed according to a published procedure.[14]

To a cooled solution (0 °C) of Ethyl (*E*)-3-(2,4-dibromo-5-(prop-2-yn-1-yloxy)phenyl)acrylate (310 mg, 0.80 mmol, 1.0 eq) in a mixture of tetrahydrofuran/water 5:1 (6 mL) was added lithium hydroxide monohydrate (50.4 mg, 1.20 mmol, 1.5 eq). The reaction mixture was then allowed to warm up to room temperature and stirred overnight. After complete consumption of the starting materials (TLC control), the excess solvent was removed under reduced pressure. The residue was taken up in water (1 mL) and acidified using a 2M HCl solution to a pH of 2-3. The aqueous layer was extracted with ethyl acetate (4 × 5 mL). The combined organic phases were washed with brine (1 × 10 mL), dried over $MgSO_4$, filtered, and concentrated *in vacuo* to yield the desired product (275 mg, 0.75 mmol, *93%*) as a white solid.

^{1}H-NMR (400 MHz, $(CD_3)_2CO$): δ [ppm] = 7.93 (s, 1H), 7.93 (d, 1H, *J* = 15.9 Hz), 7.66 (s, 1H), 6.65 (d, 1H, *J* = 15.9 Hz), 5.07 (d, 2H, *J* = 2.4 Hz), 3.20 (t, 1H, *J* = 2.4 Hz).

^{13}C-NMR (101 MHz, $(CD_3)_2CO$): δ [ppm] = 167.0, 154.8, 142.4, 137.5, 135.2, 123.2, 116.9, 115.3, 113.5, 78.6, 78.2, 57.8.

HRMS (ESI): *m/z* calcd. for $[C_{12}H_8Br_2O_3+H]^+$: 356.8767, found: 356.8769.

2,5-dioxopyrrolidin-1-yl-(*E*)-3-(2,4-dibromo-5-(prop-2-yn-1-yloxy)phenyl)acrylate (CA-2)

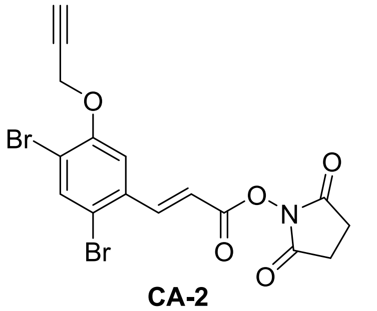

CA-2

Chemical Formula: $C_{16}H_{11}Br_2NO_5$
Molecular Weight: 457.07 g/mol

The reaction was performed in an inert atmosphere using argon according to a published procedure.[18]

To a solution of (*E*)-3-(2,4-dibromo-5-(prop-2-yn-1-yloxy)phenyl)acrylic acid (100 mg, 0.28 mmol, 1.0 eq) in a mixture of anhydrous tetrahydrofuran:dimethylformamide 9:1 (1.3 mL) was added EDC·HCl (64.2 mg, 0.34 mmol, 1.2 eq) and N-hydroxysuccinimide (41.7 mg, 0.36 mmol, 1.3 eq) at room temperature and stirred until full conversion (TLC control). The reaction was taken up in water (8 mL) and extracted with ethyl acetate (3 × 5 mL). The combined organic layers were washed with LiCl solution (5 wt%, 3 × 5 mL), brine (1 × 8 mL), dried over $MgSO_4$, filtered, and concentrated *in vacuo* to yield the desired product (112 mg, 0.25 mmol, *88%*) as a white solid.

TLC: R_f = 0.30 (pentane:ethyl acetate = 70:30 + 1% acetic acid)

^{1}H-NMR (400 MHz, $CDCl_3$): δ [ppm] = 8.20 (d, 1H, *J* = 16.0 Hz), 7.84 (s, 1H), 7.31 (s, 1H), 6.56 (d, 1H, *J* = 16.0 Hz), 4.83 (d, 2H, *J* = 2.4 Hz), 2.90 (m, 4H), 2.63 (t, 1H, *J* = 2.4 Hz).

^{13}C-NMR (101 MHz, $CDCl_3$): δ [ppm] = 169.2, 161.4, 153.8, 147.5, 137.6, 133.3, 117.9, 117.0, 115.0, 112.5, 77.5, 77.1, 57.2, 25.8.

HRMS (ESI): *m/z* calcd. for $[C_{16}H_{11}Br_2NO_5+H]^+$: 455.9076, found: 455.9074.

Ethyl (*E*)-3-(3,5-dibromo-4-hydroxyphenyl)acrylate (CA-3a)

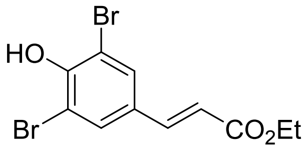

CA-3a

Chemical Formula: $C_{11}H_{10}Br_2O_3$
Molecular Weight: 350.01 g/mol

The reaction was performed in an inert atmosphere using argon according to a published procedure.[18]

3,5-dibromo-4-hydroxybenzaldehyde (0.50 g, 1.79 mmol, 1.0 eq) was dissolved in anhydrous toluene (10 mL). Then, ethyl-(triphenylphosphoranylidene)acetate (1.25 g, 3.58 mmol, 2.0 eq) was added to the solution which turned from yellow to dark brown. The mixture was heated to 70 °C under reflux for 19 h. The solution was concentrated *in vacuo*. The residue was purified via column chromatography (pentane:ethyl acetate = 100:0 + 0.5% acetic acid → 90:10 + 0.5% acetic acid) to afford the desired product (0.53 g, 1.52 mmol, 85%) as a solid.

TLC: R_f = 0.44 (pentane:ethyl acetate = 80:20 + 0.5% acetic acid)

^{1}H-NMR (500 MHz, $CDCl_3$): δ [ppm] = 7.63 (s, 2H), 7.48 (d, *J* = 15.9 Hz, 1H), 6.31 (d, *J* = 15.9 Hz, 1H), 6.07 (s, 1H), 4.25 (q, *J* = 7.1 Hz, 2H), 1.33 (t, *J* = 7.1 Hz, 3H).

These data are in accordance with previous literature reports.[18]

(*E*)-3-(3,5-dibromo-4-hydroxyphenyl)acrylic acid (CA-3b)

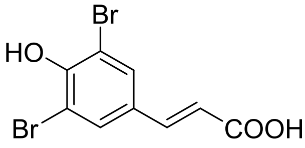

CA-3b

Chemical Formula: $C_9H_6Br_2O_3$
Molecular Weight: 321.95 g/mol

Ethyl (*E*)-3-(3,5-dibromo-4-hydroxyphenyl)acrylate (0.20 g, 0.58 mmol, 1.0 eq) was dissolved in a tetrahydrofuran:water 5:1 mixture (4 mL). After that, potassium carbonate (0.24 g, 1.73 mmol, 3.0 eq) was added and the solution was heated to 70 °C under reflux. After 15 h, the solution was cooled to 0 °C and lithium hydroxide (110 mg, 2.59 mmol, 4.5 eq) was added. The mixture was heated to 70 °C under reflux. After full conversion, tetrahydrofuran was removed *in vacuo*. Subsequently, the pH was adjusted to 1-2 by the addition of 1 M HCl. The aqueous phase was extracted with ethyl acetate (3 × 20 mL), the combined organic phases were washed with brine (1 × 70 mL), dried over $MgSO_4$, filtered and the solvent was removed *in vacuo* to obtain the desired product (187 mg, 0.58 mmol, quant. yield) as a solid.

^{1}H-NMR (500 MHz, $(CD_3)_2CO$): δ [ppm] = = 7.92 (s, 2H), 7.55 (d, *J* = 16.1 Hz, 1H), 6.52 (d, *J* = 16.1 Hz).

These data are in accordance with previous literature reports.[18]

2,5-dioxopyrrolidin-1-yl-(*E*)-3-(3,5-dibromo-4-methoxyphenyl)acrylate (CA-3)

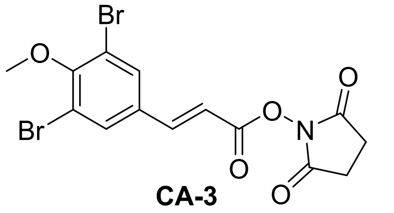

CA-3

Chemical Formula: $C_{14}H_{11}Br_2NO_5$
Molecular Weight: 433.05 g/mol

The reactions were performed in an inert atmosphere using argon according to a published procedure.[18]

(*E*)-3-(3,5-dibromo-4-hydroxyphenyl)acrylic acid (0.18 g, 0.57 mmol, 1.0 eq) was dissolved in a mixture of anhydrous tetrahydrofuran:dimethylformamide 8:1 (2.5 mL). Then, 1-Ethyl-3-(3-dimethylaminopropyl)carbodiimide hydrochloride (0.33 g, 1.70 mmol, 3.0 eq) and N-hydroxysuccinimide (0.20 g, 1.70 mmol, 3.0 eq) were added and the reaction mixture turned dark orange. The solution was stirred at room temperature for 16 h. The solvent was removed *in vacuo* and the yellow reaction mixture was partitioned between ethyl acetate (30 mL) and water (30 mL). The organic phase was separated, and the aqueous phase was extracted with ethyl acetate (2 × 30 mL). The combined organic phases were extracted with LiCl solution (5 wt%, 3 × 50 mL, washed with brine (1 × 100 mL), dried over $MgSO_4$, filtered and the solvent was removed *in vacuo* to afford the active ester. The intermediate was used for the subsequent reaction without further purification.

The intermediate active ester (0.29 g, 0.68 mmol, 1.0 eq) was dissolved in anhydrous dimethylformamide (5 mL). Then, potassium carbonate (0.19 g, 1.36 mmol, 2.0 eq), iodomethane (80.0 µL, 1.36 mmol, 2.0 eq) were added and the mixture was stirred at room temperature for 5 h. After full conversion (TLC control), water (30 mL) was added to the reaction and the solution was partitioned in diethyl ether (30 mL). The organic phase was separated, and the aqueous phase was extracted with diethyl ether (3 × 30 mL). The combined organic phases were washed with LiCl solution (5 wt%, 3 × 50 mL), washed with brine (1 × 100 mL), dried over magnesium sulfate, filtered and the solvent was removed *in vacuo*. The crude product was purified *via* column chromatography (pentane:ethyl acetate = 70:30) to afford the desired product as a solid (50 mg, 0.12 mmol, *21%*).

TLC: R_f = 0.36 (pentane:ethyl acetate = 60:40)

^{1}**H-NMR** (500 MHz, $CDCl_3$): δ [ppm] = 7.78 (d, *J* = 16.0 Hz, 1H), 7.72 (s, 2H), 6.52 (d, *J* = 15.9 Hz, 1H), 3.93 (s, 3H), 2.88 (s, 4H).

These data are in accordance with previous literature reports.[18]

(E)-N-(3-((6-((3-aminopropyl)amino)hexyl)amino)propyl)-3-(2,4-dibromo-5-methoxyphenyl)acrylamide tris trifluoroacetate(Ian-MPD-1)

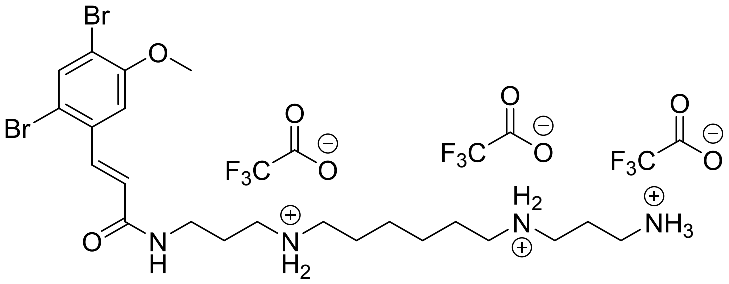

Ian-MPD-1

Chemical Formula: $C_{28}H_{39}Br_2F_9N_4O_8$
Molecular Weight: 890.43 g/mol

The reaction was performed in an inert atmosphere using argon according to a published procedure.[18]

A solution of the **CA-1** (40.0 mg, 0.09 mmol, 1.0 eq) in anhydrous dimethylformamide (2.5 mL) was added dropwise to a cooled solution (0 °C) of the **DA-1** (159 mg, 0.37 mmol, 4.0 eq) and DIPEA (19.3 µL, 0.11 mmol, 1.2 eq) in anhydrous dimethylformamide (0.3 mL). The reaction was stirred at 0 °C for approximately 10 minutes before allowing the solution to warm up to room temperature and stirred overnight. The reaction mixture was taken up in dichlormethane (10 mL) and the organic phase washed with saturated $NaHCO_3$ (5 mL). The organic layer was washed with LiCl solution (5 wt%, 3 × 5 mL) and the combined aqueous layer extracted with dichlormethane (3 × 5 mL). The combined organic phases were washed with brine (1 × 10 mL), dried over $MgSO_4$, filtered, and concentrated *in vacuo*. The crude product was dissolved in anhydrous dichlormethane (5 mL) and the solution cooled to 0 °C before addition of trifluoroacetic acid (0.5 mL, 6.53 mmol, 77 eq). The reaction was allowed to warm up to room temperature and stirred overnight. The excess solvent was removed *in vacuo* and the crude product was purifed *via* HPLC (P2, gradient 2). The fractions containing the desired product were combined and lyophilized to yield the final product (19.2 mg, 21.6 µmol, *23%*) as a colorless oil.

^{1}**H-NMR** (400 MHz, $(CD_3)_2SO$): δ [ppm] = 8.59 (br, 4H), 7.99 (br, 2H), 7.92 (s, 1H), 7.59 (d, 1H, *J* = 15.6 Hz) 7.36 (s, 1H), 6.84 (d, 1H, *J* = 15.6 Hz), 3.92 (s, 3H), 3.27 (q, 2H, *J* = 6.7 Hz), 2.94 (m, 10H), 1.86 (m, 4H), 1.58 (m, 4H), 1.31 (m, 4H).

^{13}C-NMR (101 MHz, $(CD_3)_2SO$): δ [ppm] = 164.7, 158.3, 155.2, 136.0, 135.9, 134.8, 126.1, 118.6, 115.6, 114.8, 112.8, 110.6, 56.5, 46.6, 44.7, 43.8, 36.1, 25.9, 25.4, 25.3, 25.2, 23.7.

HRMS (ESI): *m/z* calcd. for $[C_{22}H_{36}Br_2N_4O_2+H]^+$: 547.1277, found: 547.1274.

These data are in accordance with previous literature reports.[14]

(*E*)-N-(3-((6-((3-aminopropyl)amino)hexyl)amino)propyl)-3-(2,4-dibromo-5-(prop-2-yn-1-yloxy)phenyl)acrylamide tris trifluoroacetate (Ian-P-1)

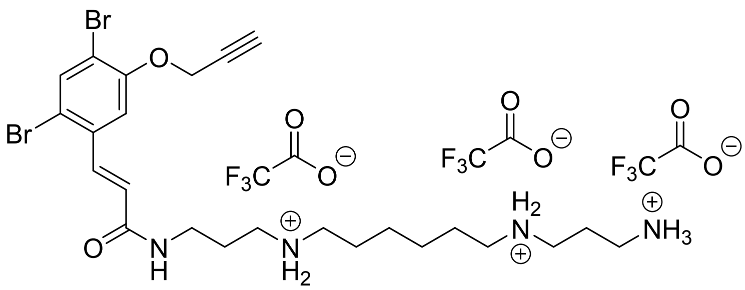

Ian-P-1

Chemical Formula: $C_{30}H_{39}Br_2F_9N_4O_8$
Molecular Weight: 914.46 g/mol

The reaction was performed in an inert atmosphere using argon according to a published procedure.[18]

To a cooled solution (0 °C) of **DA-1** (146 mg, 0.340 mmol, 4.0 eq) in anhydrous dimethylformamide (0.3 mL) was added dropwise a solution of the **CA-2** (38.9 mg, 85 µmol, 1.0 eq) in anhydrous dimethylformamide (2.5 mL). The reaction was stirred at 0 °C for approximately 10 minutes before allowing the solution to warm up to room temperature and left at room temperature overnight. The reaction mixture was taken up in dichlormethane (10 mL) and saturated sodium bicarbonate solution (5 mL) and the layers separated. The organic layer was washed with LiCl solution (5 wt%, 3 × 5 mL), brine (1 × 10 mL), dried over MgSO4, filtered and concentrated *in vacuo*. The crude product was dissolved in anhydrous dichlormethane (5 mL) and the solution cooled to 0 °C before the addition of trifluoroacetic acid (0.5 ml, 6.53 mmol, 77 eq). The reaction was allowed to warm up to room temperature and stirred overnight. The excess solvent was removed *in vacuo* and the crude product was purified *via* HPLC (P2, gradient 2) to yield the desired product (19.6 mg, 21.4 µmol, 25%) as a colorless viscose oil.

^{1}H-NMR (400 MHz, $(CD_3)_2SO$): δ [ppm] = 8.73 (br, 4H), 8.01 (br, 2H), 7.96 (s, 1H), 7.60 (d, 2H, , *J* = 15.7 Hz), 7.48 (s, 1H), 6.80 (d, 1H, *J* = 15.7 Hz), 5.03 (d, 2H, *J* = 15.7 Hz), 3.68 (t, 1H, *J* = 2.3 Hz), 3.26 (q, 2H, *J* = 6.3 Hz), 2.92 (m, 10H), 1.84 (m, 4H), 1.57 (m, 4H), 1.31 (m, 4H).

^{13}C-NMR (101 MHz, $(CD_3)_2SO$): δ [ppm] = 164.7, 153.2, 136.3, 136.0, 134.6, 126.1, 120.7, 118.3, 116.0, 115.7, 113.6, 113.6, 113.4, 112.3, 79.3, 78.2, 56.8, 46.7, 44.7, 43.9, 36.2, 36.1, 26.0, 25.5, 25.4, 25.3, 23.8.

HRMS (ESI): *m/z* calcd. for $[C_{24}H_{36}Br_2N_4O_2+H]^+$: 571.1278, found: 571.1284.

(*E*)-N-(3-((4-((3-aminopropyl)amino)butyl)amino)propyl)-3-(3,5-dibromo-4-methoxyphenyl)acrylamide tris trifluoroacetate (Ian-A)

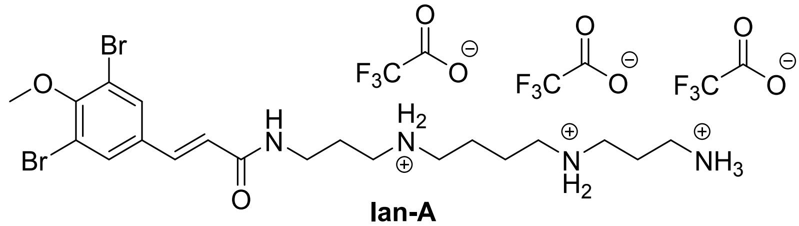

Ian-A

Chemical Formula: $C_{26}H_{35}Br_2F_9N_4O_8$
Molecular Weight: 862.38 g/mol

The reaction was performed in an inert atmosphere using argon according to a published procedure.[18]

DA-2 (0.19 g, 0.46 mmol, 4.0 eq) was dissolved in anhydrous dimethylformamide (0.4 mL) and cooled to 0 °C. *N*,*N*-diisopropylethylamine (24.0 µL, 0.14 mmol, 1.2 eq) was added to the mixture and stirred for 10 min. Then, in a separated flask **CA-3** (50 mg, 0.12 mmol, 1.0 eq) was dissolved in anhydrous dimethylformamide (2.6 mL) and the mixture was added dropwise to the reaction. The reaction mixture was allowed to warm up to room temperature and stirred until full conversion. After that, the solution was partitioned between dichlormethane (30 mL) and saturated sodium hydrogen carbonate solution (30 mL). The aqueous phase was separated, and the organic phase was washed with LiCl solution (5wt%, 3 × 20 mL). The combined organic phases were washed with brine (1 × 150 mL), dried over $MgSO_4$, filtered and the solvent was removed *in vacuo* to afford Boc-protected intermediate. The intermediate was used for subsequent experiments without further purification. Boc-protected Ianthelliformisamine A (0.28 g, 0.38 mmol, 1.0 eq) was dissolved in anhydrous dichlormethane (4.5 mL) and cooled to 0 °C. After that, trifluoroacetic acid (1.0 mL, 13.1 mmol, 34.0 eq.) was added, the solution was stirred for 10 min and then allowed to warm up to room temperature. After full conversion the solvent was removed *in vacuo*. The crude was purified *via* HPLC (P1, gradient 3) to obtain the desired product a white solid (24.0 mg, 0.03 mmol, *25%*).

^{1}H-NMR (500 MHz, CD_3OD): δ [ppm] = 7.81 (s, 2H), 7.43 (d, *J* = 15.7 Hz), 6.58 (d, *J* = 15.7 Hz), 3.88 (s, 3H), 3.43 (t, *J* = 6.6 Hz, 2H), 3.14 (t, *J* = 7.8 Hz, 2H), 3.11 – 3.02 (m, 8H), 2.12 – 2.04 (m, 2H), 1.98 – 1.92 (m, 2H), 1.85 – 1.79 (m, 4H).

^{13}C-NMR (126 MHz, CD_3OD): δ [ppm] = 168.8, 156.5, 138.8, 135.2, 133.1, 123.3, 119.6, 61.2, 48.3, 48.1, 46.4, 45.9, 37.8, 37.1, 27.8, 25.4, 24.33, 24.27.

HRMS (ESI): *m/z* calcd. for $[C_{20}H_{32}Br_2N_4O_2+H]^+$: 519.0965, found: 519.0961

These data are in accordance with previous literature reports.[20]

Di-tert-butyl-butane-1,4-diylbis((3-((*E*)-3-(3,5-dibromo-4-methoxyphenyl)acrylamido)propyl)carbamate)

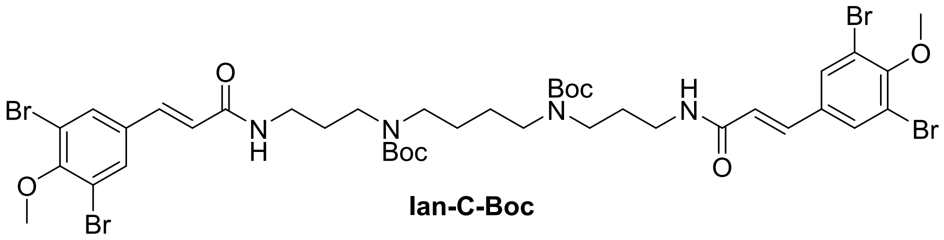

Ian-C-Boc

Chemical Formula: $C_{40}H_{54}Br_4N_4O_8$

Molecular Weight: 1038.51 g/mol

The reaction was performed in an inert atmosphere using argon according to a published procedure.[18]

CA-3 (50.0 mg, 0.11 mmol, 2.5 eq) was added in a flask. **DA-2** (20.0 mg, 0.04 mmol, 1.0 eq) was dissolved in anhydrous 1,4-dioxane (1 mL) and the diamine solution was added to **CA-3**. The mixture was stirred at room temperature for 24 h. Then, the solution was partitioned between dichlormethane (20 mL) and saturated sodium hydrogen carbonate solution (20 mL). The aqueous phase was separated, and the organic phase was washed with saturated sodium hydrogen carbonate solution (3 × 20 mL). The combined organic phases were washed with brine (1 × 150 mL), dried over $MgSO_4$, filtered and the solvent was removed *in vacuo*. The crude product was purified *via* column chromatography (dichloromethane:methanol = 97.5/2.5 → 95/5) to afford the desired product (40.1 mg, 0.04 mmol, 88%) as a solid.

TLC: R_f = 0.11 (dichloromethane:methanol = 95/5)

^{1}H-NMR (500 MHz, $CDCl_3$): δ [ppm] = 7.62 (s, 4H), 7.42 (d, *J* = 15.6 Hz, 2H), 6.37 (d, *J* = 15.6 Hz, 2H), 3.88 (s, 6H), 3.38 – 3.27 (m, 8H), 3.20 – 3.09 (m, 4H), 1.74 – 1.66 (m, 4H), 1.47 (s, 18H), 1.31 – 1.20 (m, 4H).

These data are in accordance with previous literature reports.[18]

(2*E*,2'*E*)-N,N'-((butane-1,4-diylbis(azanediyl))bis(propane-3,1-diyl))bis(3-(3,5-dibromo-4-methoxyphenyl)acrylamide) bis trifluoroacetate (Ian-C)

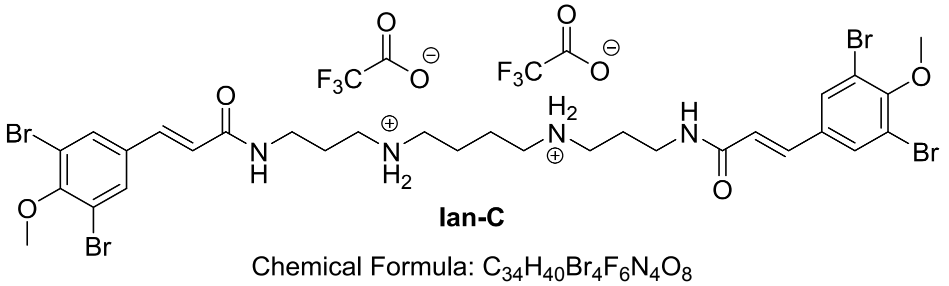

Chemical Formula: $C_{34}H_{40}Br_4F_6N_4O_8$
Molecular Weight: 1066.32 g/mol

Ian-C-Boc (50 mg, 0.05 mmol, 1.0 eq) was dissolved in anhydrous dichlormethane (0.8 mL) and cooled to 0 °C. After that, trifluoroacetic acid (0.20 mL, 2.61 mmol, 54.2 eq) was added, the solution was stirred for 10 min and then allowed to warm up to room temperature. After 20 h, the solvent was removed *in vacuo*. The crude product was purified *via* HPLC (P1, gradient 3) to afford the desired product as a white solid (9.30 mg, 0.01 mmol, 18%).

^{1}H-NMR (500 MHz, CD_3OD): δ [ppm] = 7.77 (s, 4H), 7.41 (d, *J* = 15.7 Hz, 2H), 6.57 (d, *J* = 15.7 Hz, 2H), 3.87 (s, 6H), 3.44 (t, *J* = 6.3 Hz, 4H), 3.12 – 3.04 (m, 8H), 1.99 – 1.93 (m, 4H), 1.87 – 1.83 (m, 4H).

^{13}C-NMR (126 MHz, CD_3OD): δ [ppm] = 168.9, 156.5, 138.9, 135.1, 133.1, 123.2, 119.6, 61.2, 48.2, 46.3, 37.0, 27.8, 24.3.

HRMS (ESI): *m/z* calcd. for $[C_{30}H_{38}Br_4N_4O_4+H]^+$: 834.9700, found: 837.9689

These data are in accordance with previous literature reports.[18]

Literature

[1] C. L. M. Gilchrist, M. Mirdita, M. Steinegger, **2024**, bioRxiv preprint, DOI: 10.1101/2024.08.01.606130.

[2] T. Reinhardt, K. M. Lee, L. Niederegger, C. R. Hess, S. A. Sieber, "Indolin-2-one Nitroimidazole Antibiotics Exhibit an Unexpected Dual Mode of Action" *ACS Chem. Biol.* **2022**, *17*, 3077–3085.

[3] G. Sautrey, M. E. Khoury, A. G. dos Santos, L. Zimmermann, M. Deleu, L. Lins, J.-L. Décout, M.-P. Mingeot-Leclercq, "Negatively Charged Lipids as a Potential Target for New Amphiphilic Aminoglycoside Antibiotics: A BIOPHYSICAL STUDY *" *JBC* **2016**, *291*, 13864–13874.

[4] I. Hübner, J. A. Shapiro, J. Hoßmann, J. Drechsel, S. M. Hacker, P. N. Rather, D. H. Pieper, W. M. Wuest, S. A. Sieber, "Broad Spectrum Antibiotic Xanthocillin X

Effectively Kills Acinetobacter baumannii via Dysregulation of Heme Biosynthesis" *ACS Cent. Sci.* **2021**, *7*, 488–498.

[5] P. Le, E. Kunold, R. Macsics, K. Rox, M. C. Jennings, I. Ugur, M. Reinecke, D. Chaves-Moreno, M. W. Hackl, C. Fetzer, F. A. M. Mandl, J. Lehmann, V. S. Korotkov, S. M. Hacker, B. Kuster, I. Antes, D. H. Pieper, M. Rohde, W. M. Wuest, E. Medina, S. A. Sieber, "Repurposing human kinase inhibitors to create an antibiotic active against drug-resistant Staphylococcus aureus, persisters and biofilms" *Nat. Chem.* **2020**, *12*, 145–158.

[6] D. Mostert, J. Braun, M. D. Zimmerman, C. A. Engelhart, S. Berndl, P. K. Quoika, A. M. Kany, J. Proietto, S. Penalva-Lopez, J. B. Wallach, A. K. H. Hirsch, M. Zacharias, D. Schnappinger, V. Dartois, S. A. Sieber, "Tailored phenyl ureas eradicate drug-resistant Mycobacterium tuberculosis by targeting mycolic acid cell wall assembly" *Chem. Sci.* **2025**, *16*, 9472–9483.

[7] F. Coscia, S. Doll, J. M. Bech, L. Schweizer, A. Mund, E. Lengyel, J. Lindebjerg, G. I. Madsen, J. M. Moreira, M. Mann, "A streamlined mass spectrometry–based proteomics workflow for large-scale FFPE tissue analysis" *J. pathol.* **2020**, *251*, 100–112.

[8] V. Demichev, C. B. Messner, S. I. Vernardis, K. S. Lilley, M. Ralser, "DIA-NN: neural networks and interference correction enable deep proteome coverage in high throughput" *Nat Methods* **2020**, *17*, 41–44.

[9] S. Tyanova, T. Temu, P. Sinitcyn, A. Carlson, M. Y. Hein, T. Geiger, M. Mann, J. Cox, "The Perseus computational platform for comprehensive analysis of (prote)omics data" *Nat Methods* **2016**, *13*, 731–740.

[10] M. Wieland, M. Holm, E. J. Rundlet, M. Morici, T. O. Koller, T. P. Maviza, D. Pogorevc, I. A. Osterman, R. Müller, S. C. Blanchard, D. N. Wilson, "The cyclic octapeptide antibiotic argyrin B inhibits translation by trapping EF-G on the ribosome during translocation" *Proc. Natl. Acad. Sci. U.S.A.* **2022**, *119*, e2114214119.

[11] D. B. Allan, T. Caswell, N. C. Keim, C. M. van der Wel, R. W. Verweij **2021**, DOI 10.5281/zenodo.4682814.

[12] S. van der Walt, J. L. Schönberger, J. Nunez-Iglesias, F. Boulogne, J. D. Warner, N. Yager, E. Gouillart, T. Yu, "scikit-image: image processing in Python" *PeerJ* **2014**, *2*, e453.

[13] C. R. Harris, K. J. Millman, S. J. van der Walt, R. Gommers, P. Virtanen, D. Cournapeau, E. Wieser, J. Taylor, S. Berg, N. J. Smith, R. Kern, M. Picus, S. Hoyer, M. H. van Kerkwijk, M. Brett, A. Haldane, J. F. del Río, M. Wiebe, P. Peterson, P. Gérard-Marchant, K. Sheppard, T. Reddy, W. Weckesser, H. Abbasi, C. Gohlke, T. E. Oliphant, "Array programming with NumPy" *Nature* **2020**, *585*, 357–362.

[14] F. A. Khan, S. Ahmad, N. Kodipelli, G. Shivange, R. Anindya, “Syntheses of a library of molecules on the marine natural product ianthelliformisamines platform and their biological evaluation” *Org. Biomol. Chem.* **2014**, *12*, 3847–3865.

[15] P. Singh, C. Samorì, F. M. Toma, C. Bussy, A. Nunes, K. T. Al-Jamal, C. Ménard-Moyon, M. Prato, K. Kostarelos, A. Bianco, “Polyamine functionalized carbon nanotubes: synthesis, characterization, cytotoxicity and siRNA binding” *J. Mater. Chem.* **2011**, *21*, 4850–4860.

[16] M. Ramírez Osuna, G. Aguirre, R. Somanathan, E. Molins, “Asymmetric synthesis of amathamides A and B: novel alkaloids isolated from *Amathia wilsoni*” *Tetrahedron: Asymmetry* **2002**, *13*, 2261–2266.

[17] R. Meiß, K. Kumar, H. Waldmann, “Divergent Gold(I)-Catalyzed Skeletal Rearrangements of 1,7-Enynes” *Chem. Eur. J.* **2015**, *21*, 13526–13530.

[18] R. A. Allen, C. E. M. McCormack, W. M. Wuest, “Deriving Novel Quaternary Ammonium Compound Disinfectant Scaffolds from a Natural Product: Mechanistic Insights of the Quaternization of Ianthelliformisamine C” *ChemMedChem* **2023**, *18*, e202300253.

[19] R. H. Hans, E. M. Guantai, C. Lategan, P. J. Smith, B. Wan, S. G. Franzblau, J. Gut, P. J. Rosenthal, K. Chibale, “Synthesis, antimalarial and antitubercular activity of acetylenic chalcones” *Bioorg. Med. Chem. Lett.* **2010**, *20*, 942–944.

[20] C. Pieri, D. Borselli, C. Di Giorgio, M. De Méo, J.-M. Bolla, N. Vidal, S. Combes, J. M. Brunel, “New Ianthelliformisamine Derivatives as Antibiotic Enhancers against Resistant Gram-Negative Bacteria” *J. Med. Chem.* **2014**, *57*, 4263–4272.

^{1}H, ^{13}C-NMR spectra

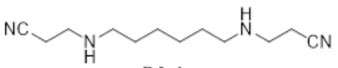

DA-1a

Chemical Formula: $C_{12}H_{22}N_4$
Molecular Weight: 222.33 g/mol

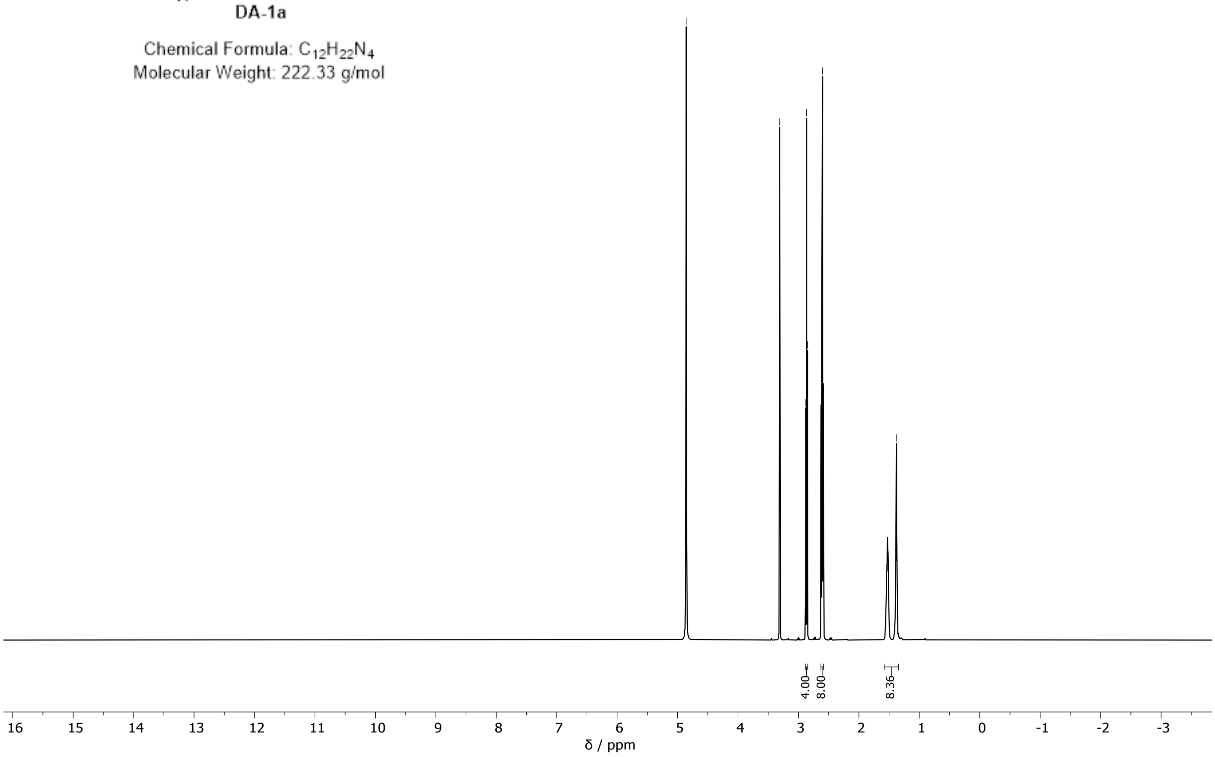

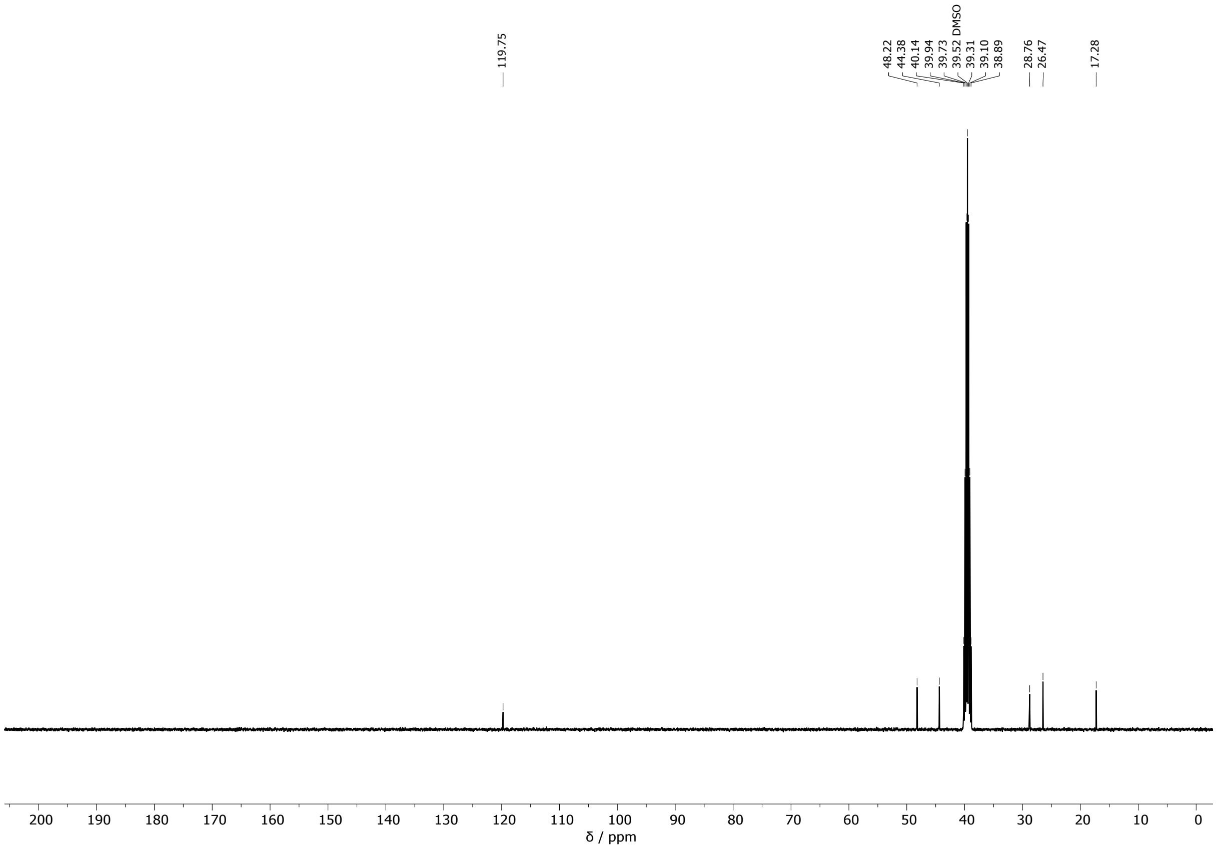

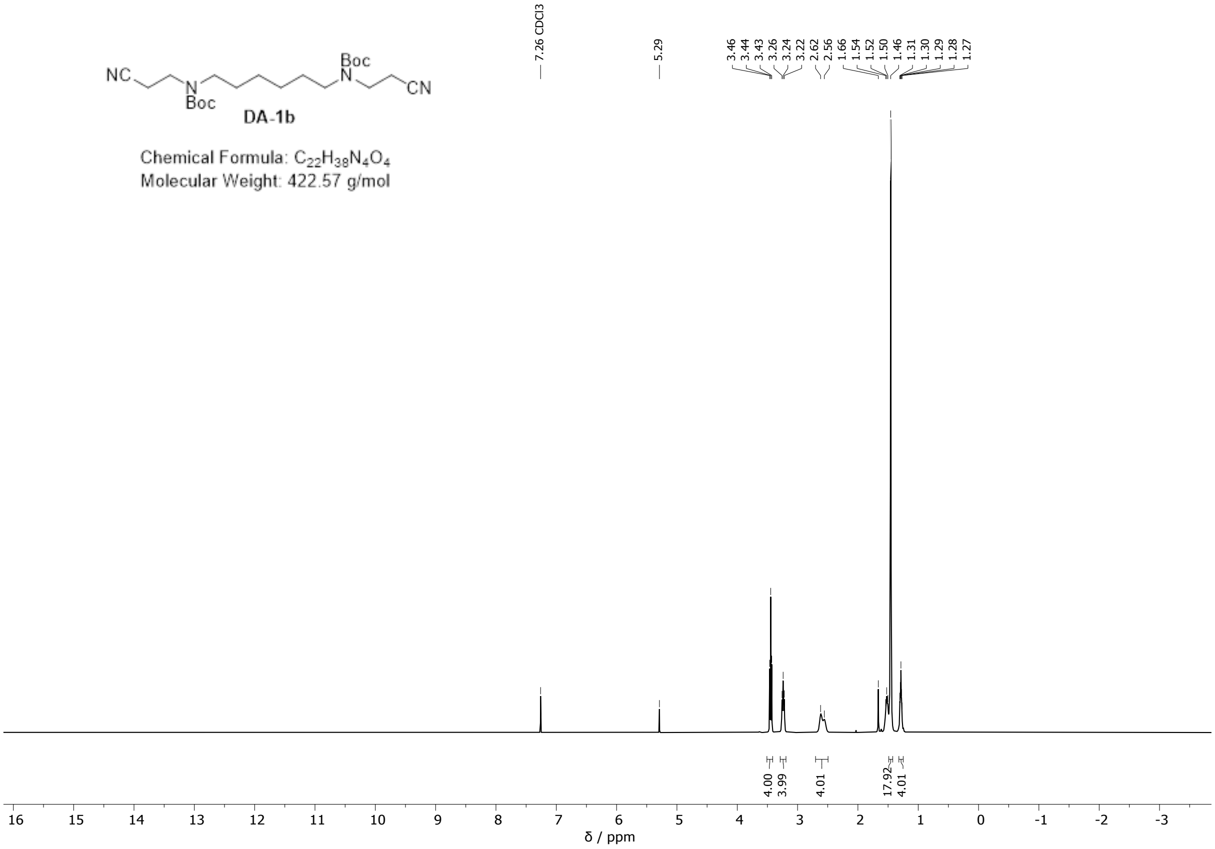
NC
N
Boc
Boc
N
CN
DA-1b
Chemical Formula: C22H38N4O4
Molecular Weight: 422.57 g/mol
7.26 CDCl3
5.29
3.46
3.44
3.43
3.26
3.24
3.22
2.62
2.56
1.66
1.54
1.52
1.50
1.46
1.31
1.30
1.29
1.28
1.27
4.00
3.99
4.01
17.92
4.01
16 15 14 13 12 11 10 9 8 7 6 5 4 3 2 1 0 -1 -2 -3
δ / ppm

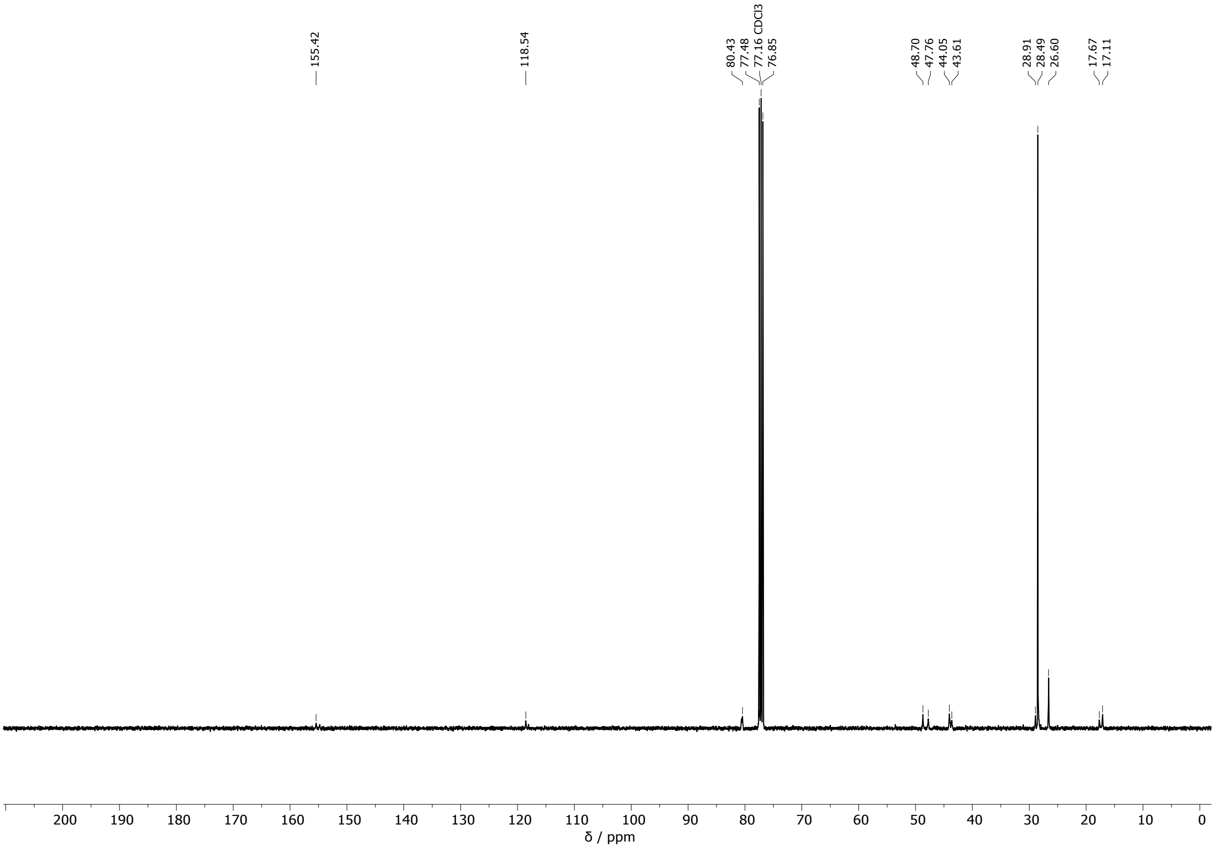
155.42
118.54
80.43
77.48
77.16 CDCl3
76.85
48.70
47.76
44.05
43.61
28.91
28.49
26.60
17.67
17.11
200 190 180 170 160 150 140 130 120 110 100 90 80 70 60 50 40 30 20 10 0
δ / ppm

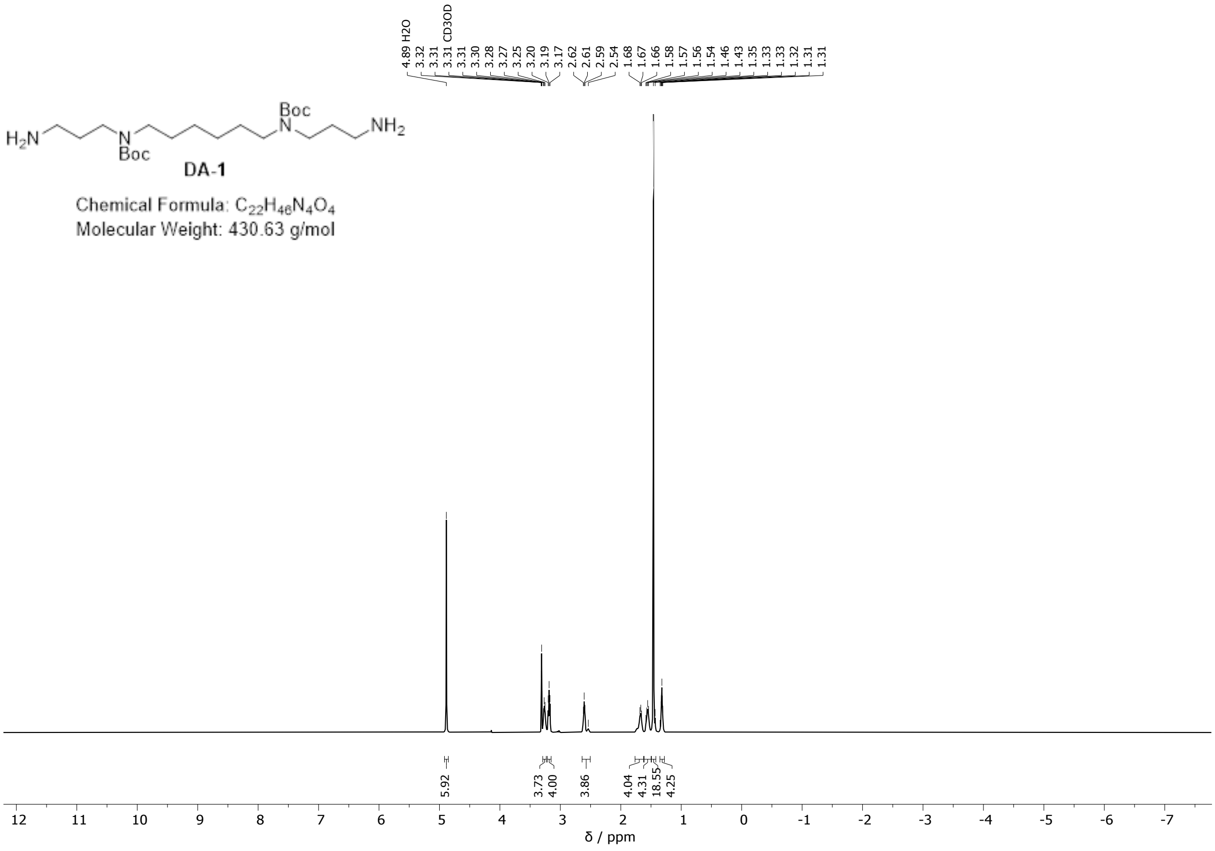

DA-1

Chemical Formula: $C_{22}H_{46}N_4O_4$

Molecular Weight: 430.63 g/mol

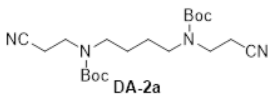

DA-2a

Chemical Formula: $C_{20}H_{34}N_4O_4$
Molecular Weight: 394.52 g/mol

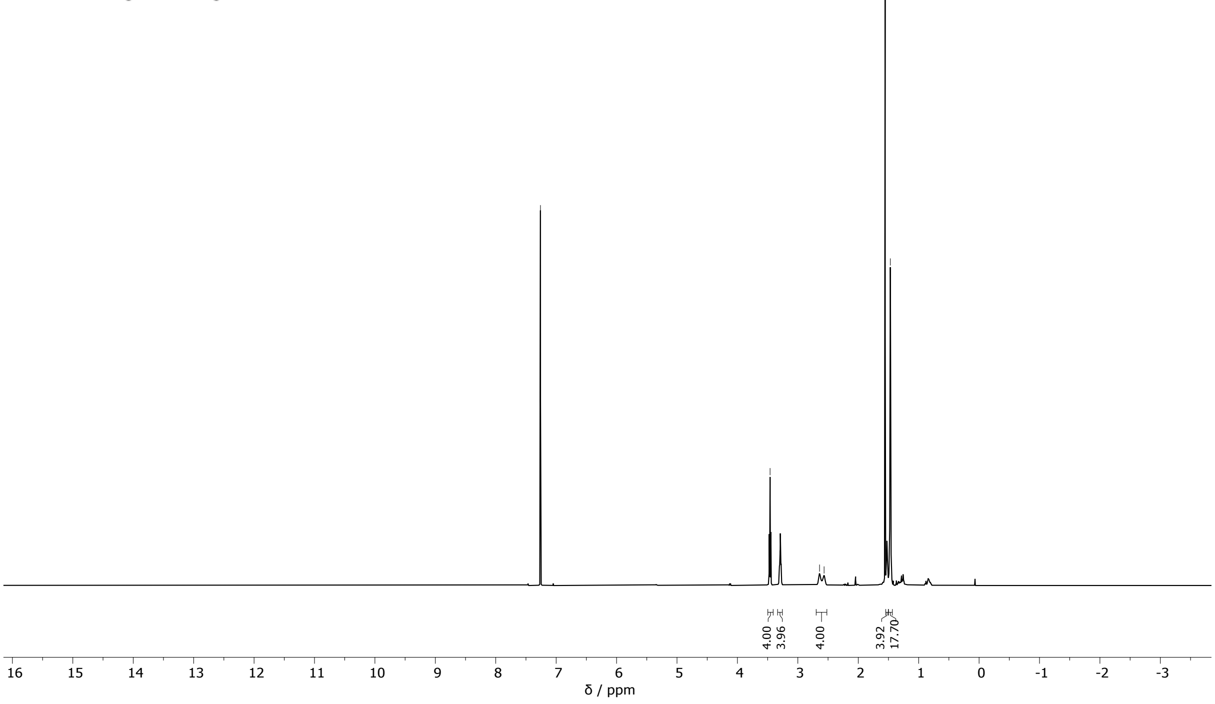

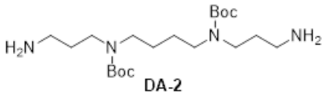

DA-2

Chemical Formula: $C_{20}H_{42}N_4O_4$
Molecular Weight: 402.58 g/mol

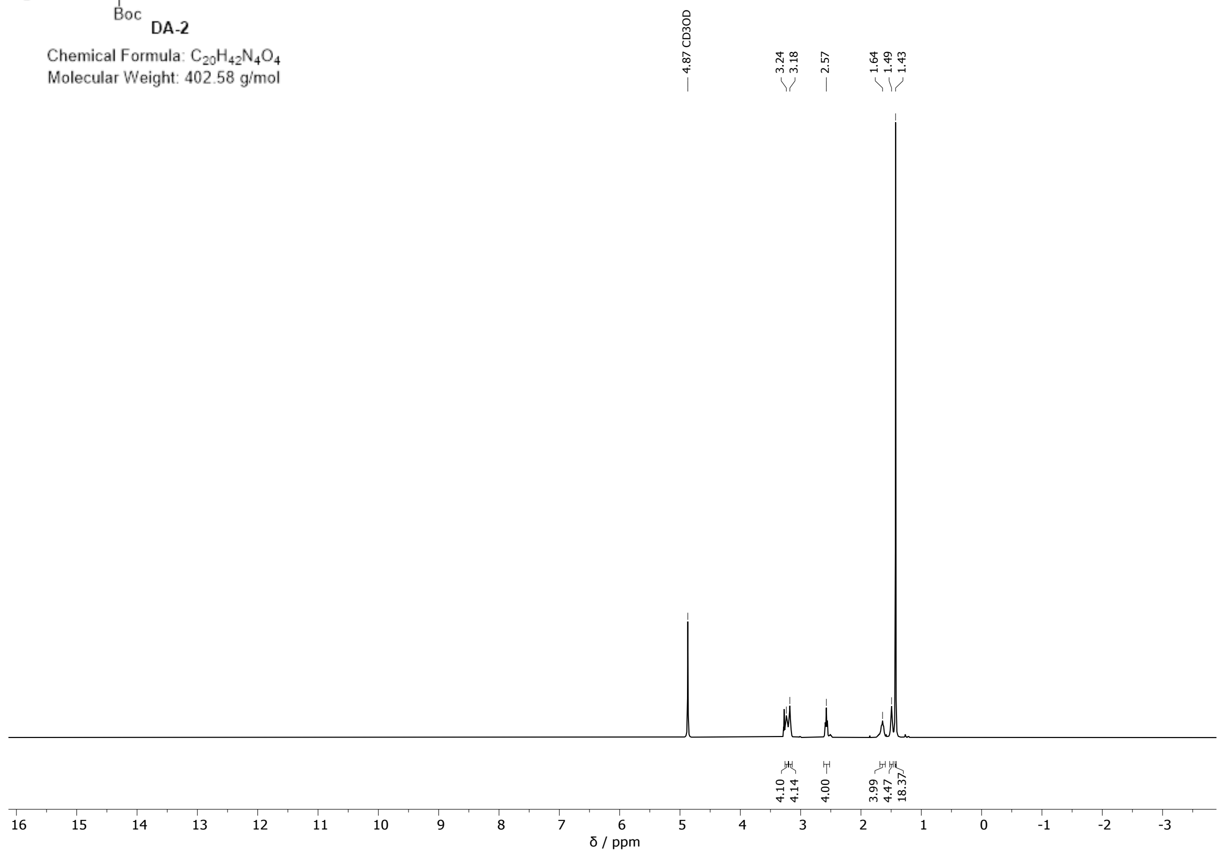

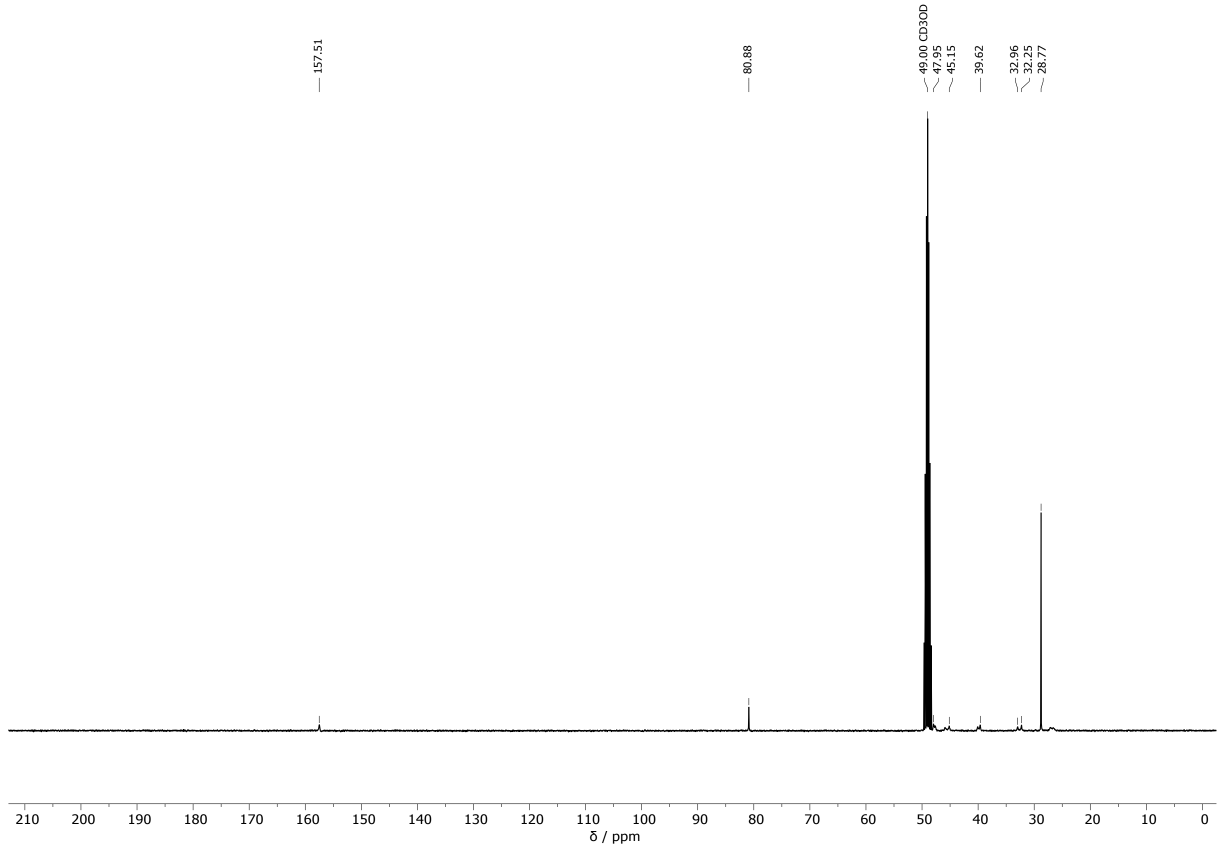

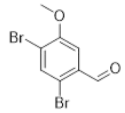

CA-1a

Chemical Formula: $C_8H_6Br_2O_2$
Molecular Weight: 293.94 g/mol

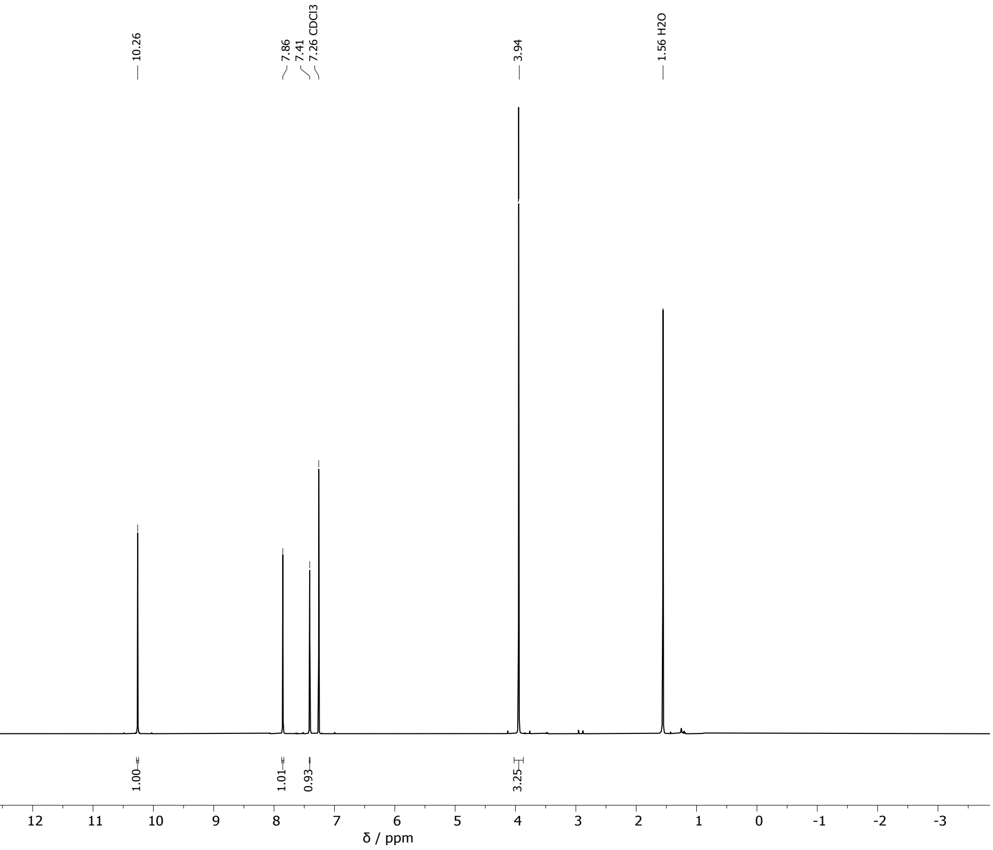

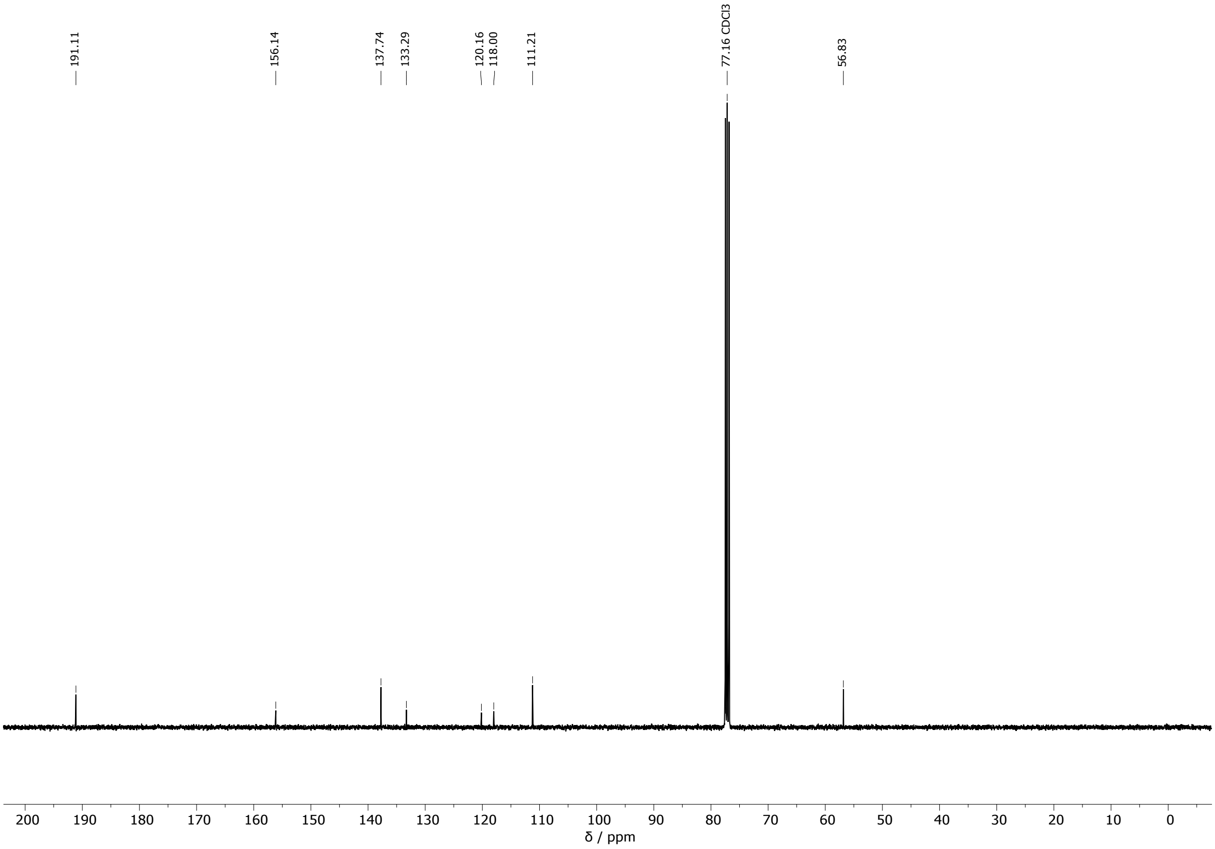

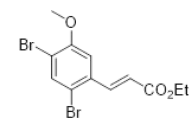

CA-1b

Chemical Formula: $C_{12}H_{12}Br_2O_3$

Molecular Weight: 364.03 g/mol

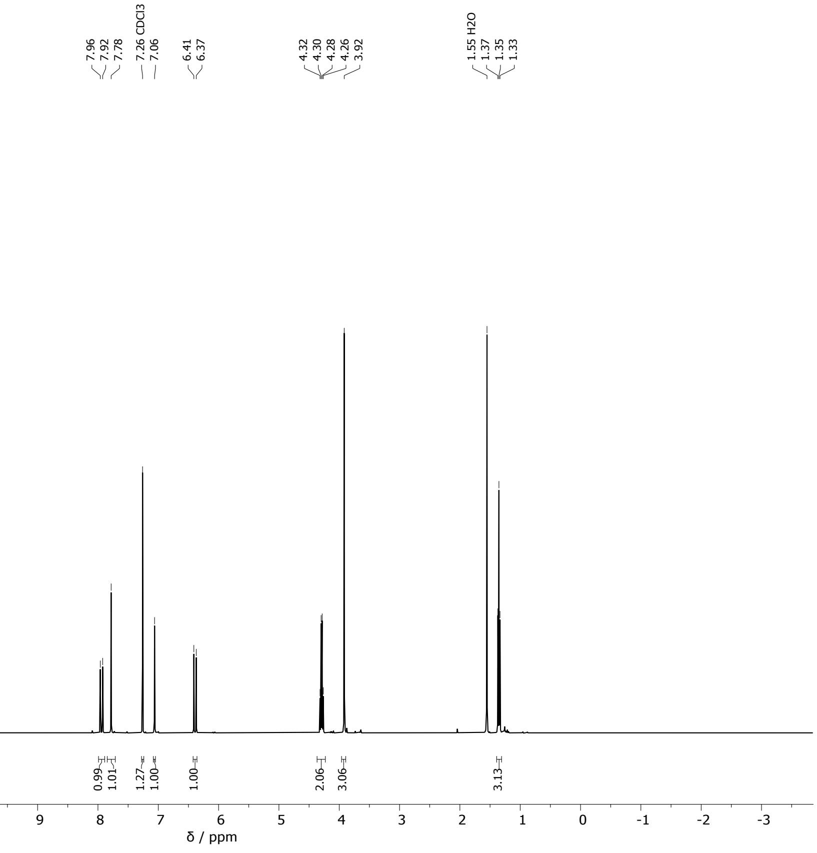

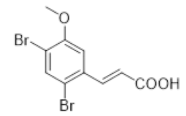

CA-1c

Chemical Formula: $C_{10}H_8Br_2O_3$

Molecular Weight: 335.98 g/mol

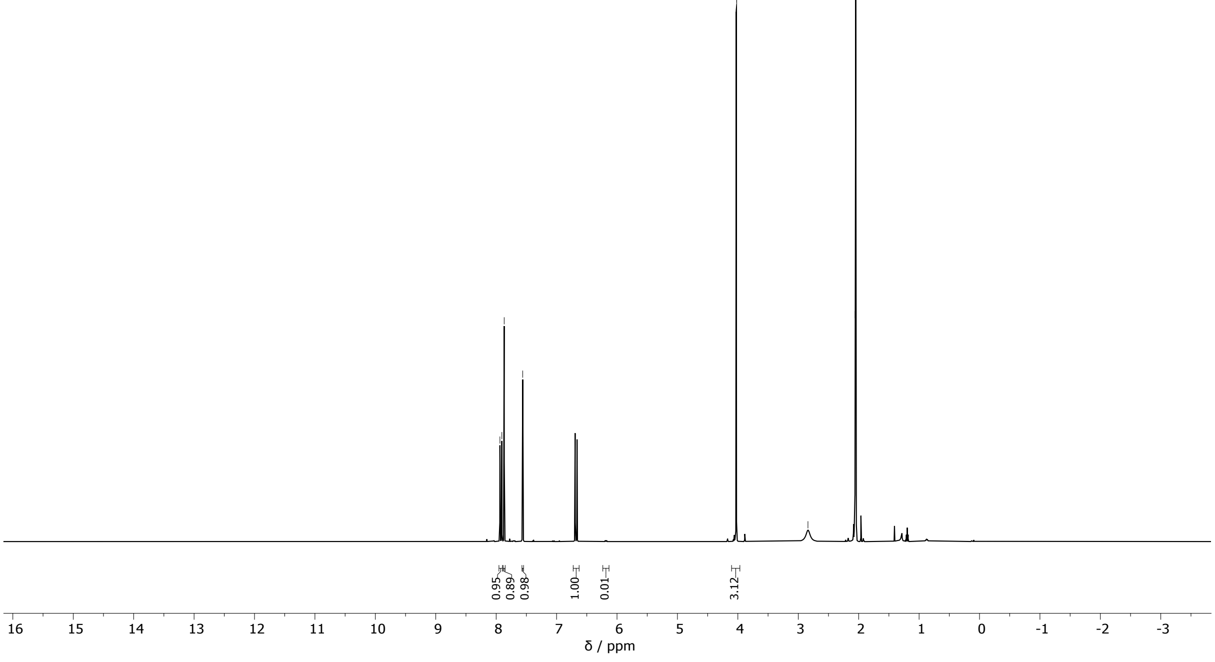

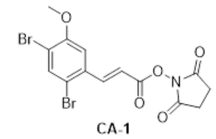

CA-1

Chemical Formula: $C_{14}H_{11}Br_2NO_5$
Molecular Weight: 433.05 g/mol

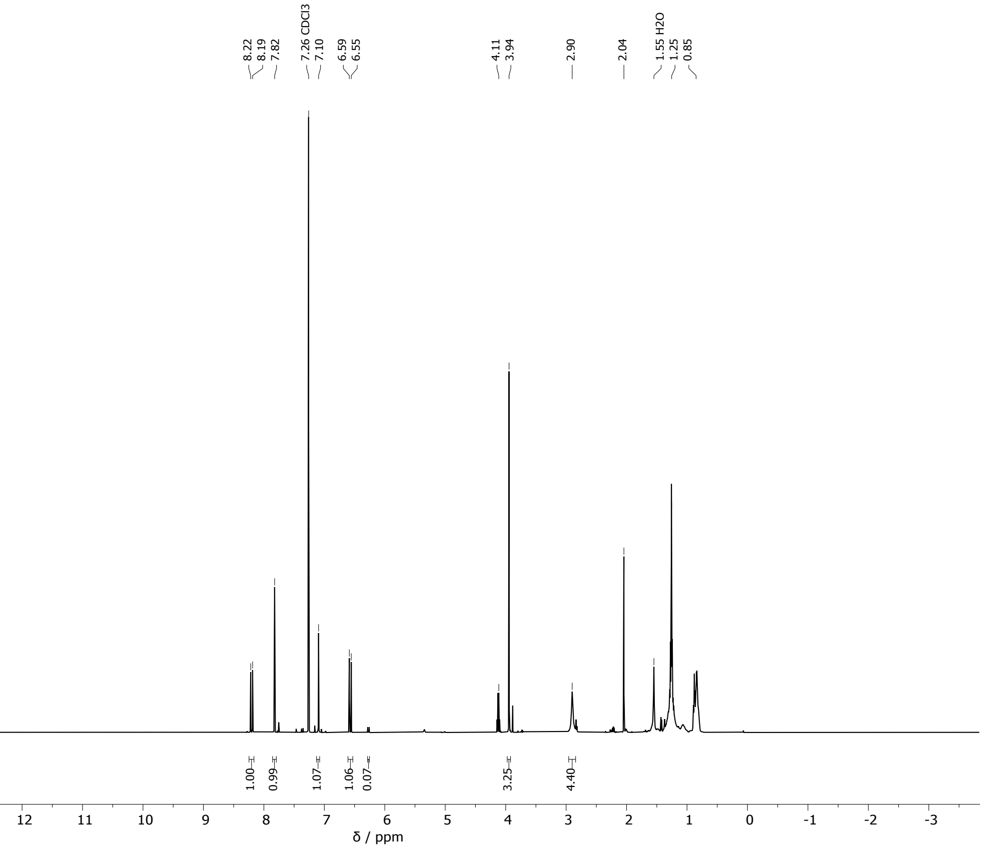

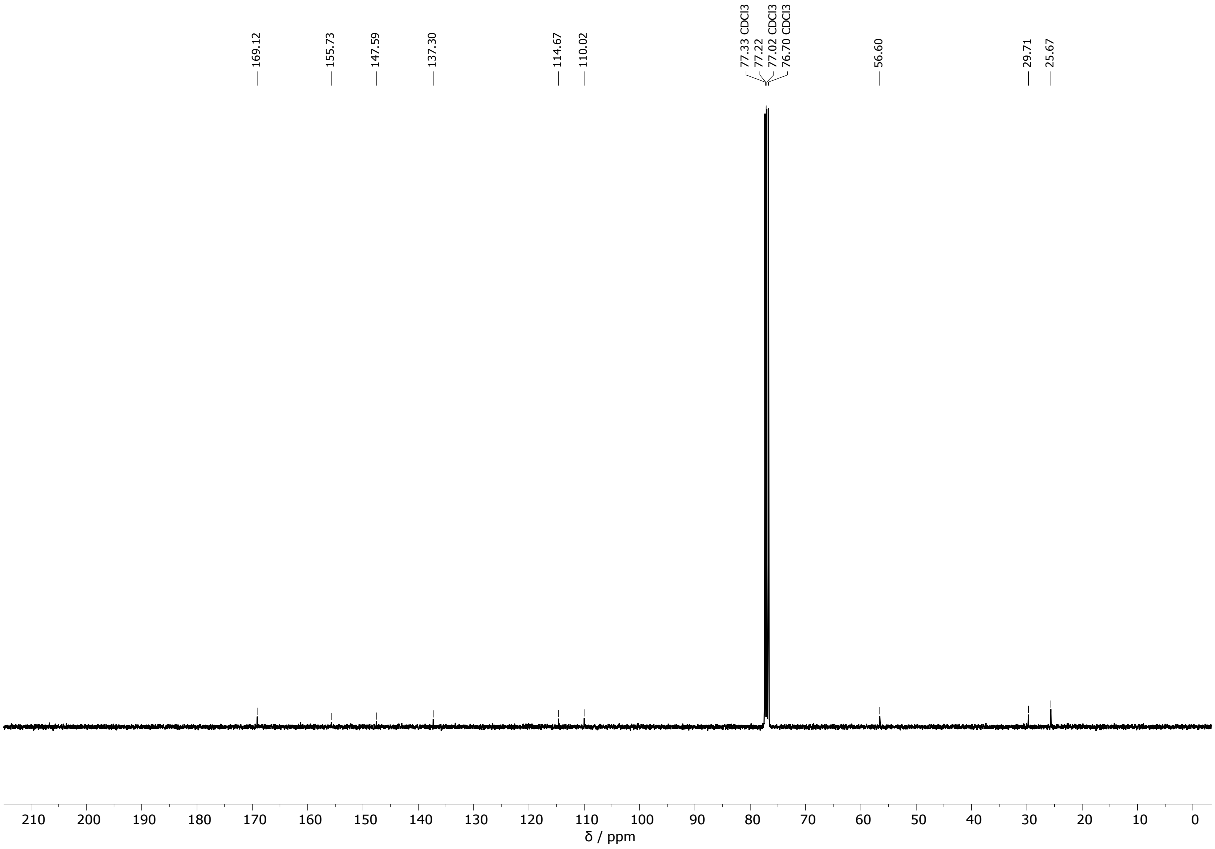

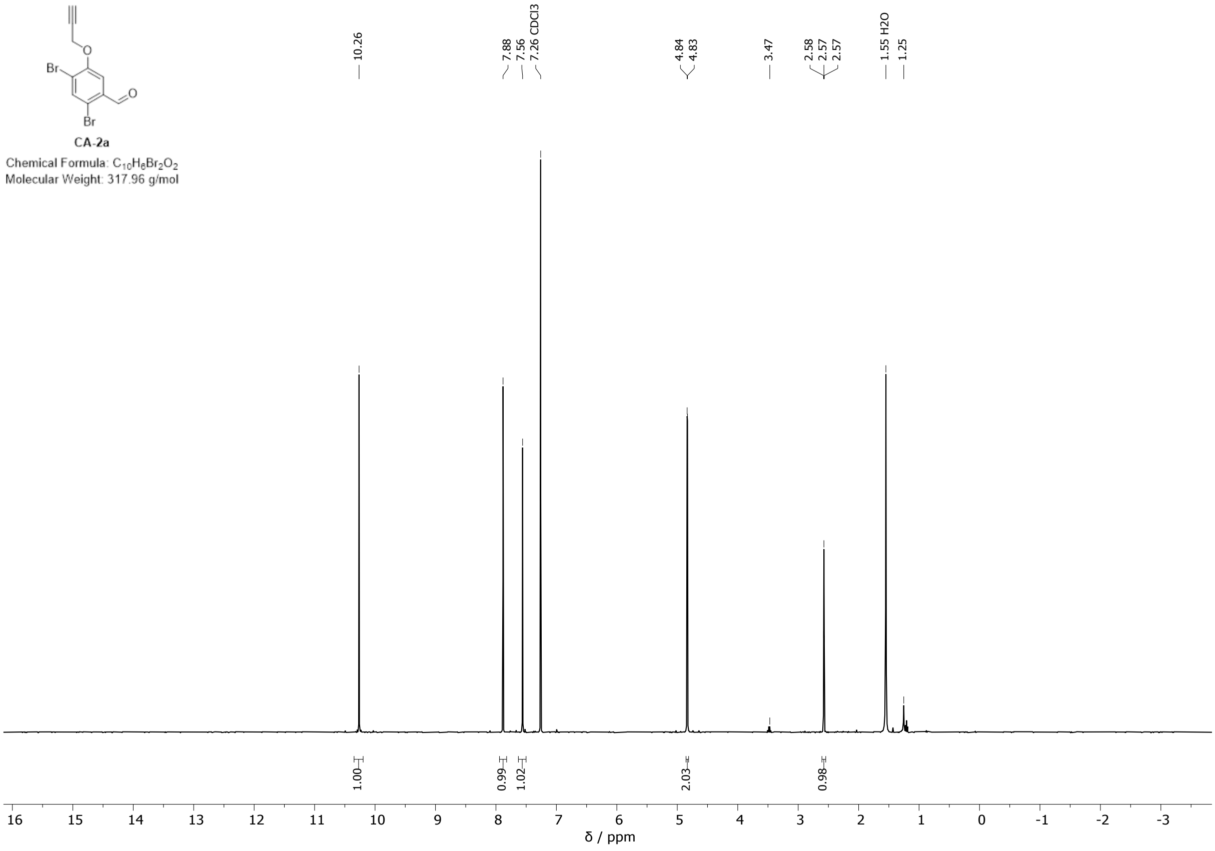
Br
O
Br
Br
CA-2a
Chemical Formula: $C_{10}H_6Br_2O_2$
Molecular Weight: 317.96 g/mol
10.26
7.88
7.56
7.26 CDCl3
4.84
4.83
3.47
2.58
2.57
2.57
1.55 H2O
1.25
1.00
0.99
1.02
2.03
0.98
δ / ppm

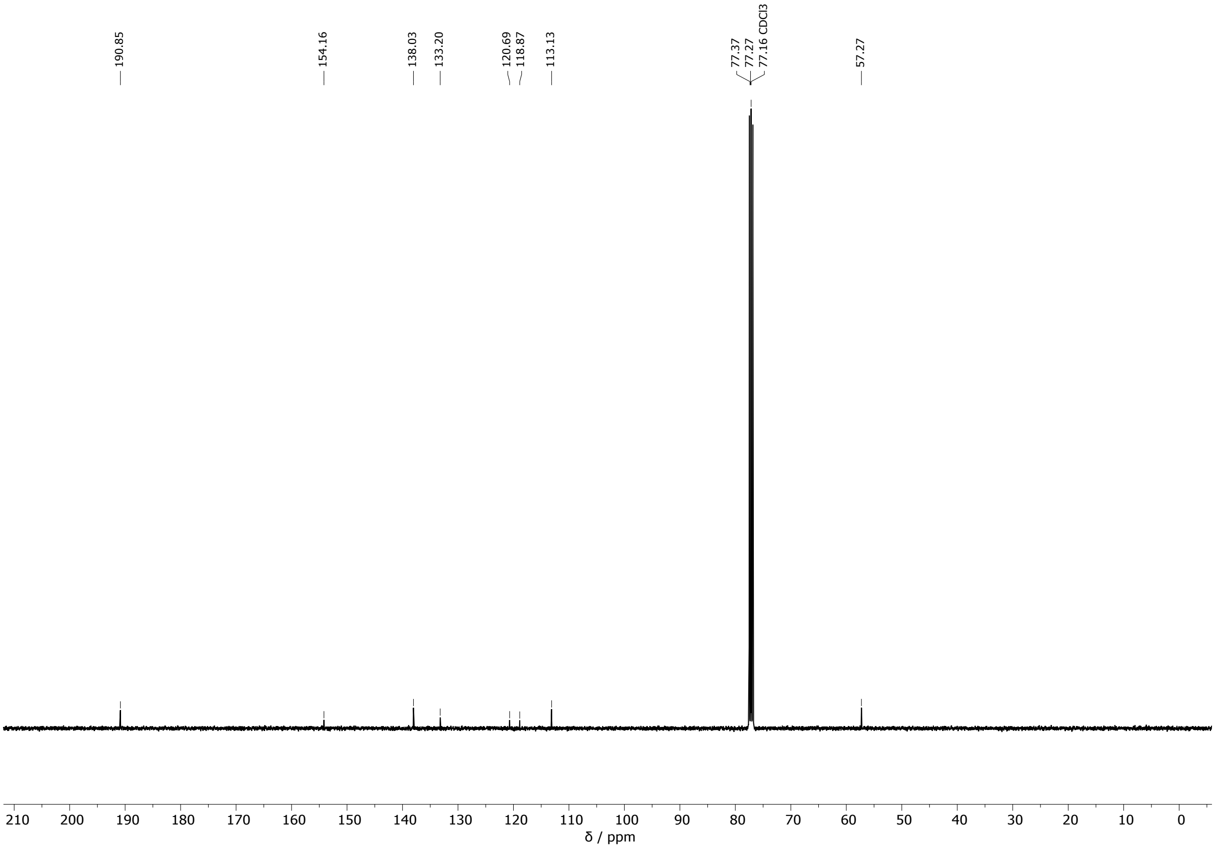
190.85
154.16
138.03
133.20
120.69
118.87
113.13
77.37
77.27
77.16 CDCl3
57.27
δ / ppm

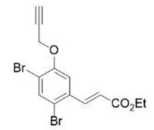

CA-2b

Chemical Formula: $C_{14}H_{12}Br_2O_3$

Molecular Weight: 388.06 g/mol

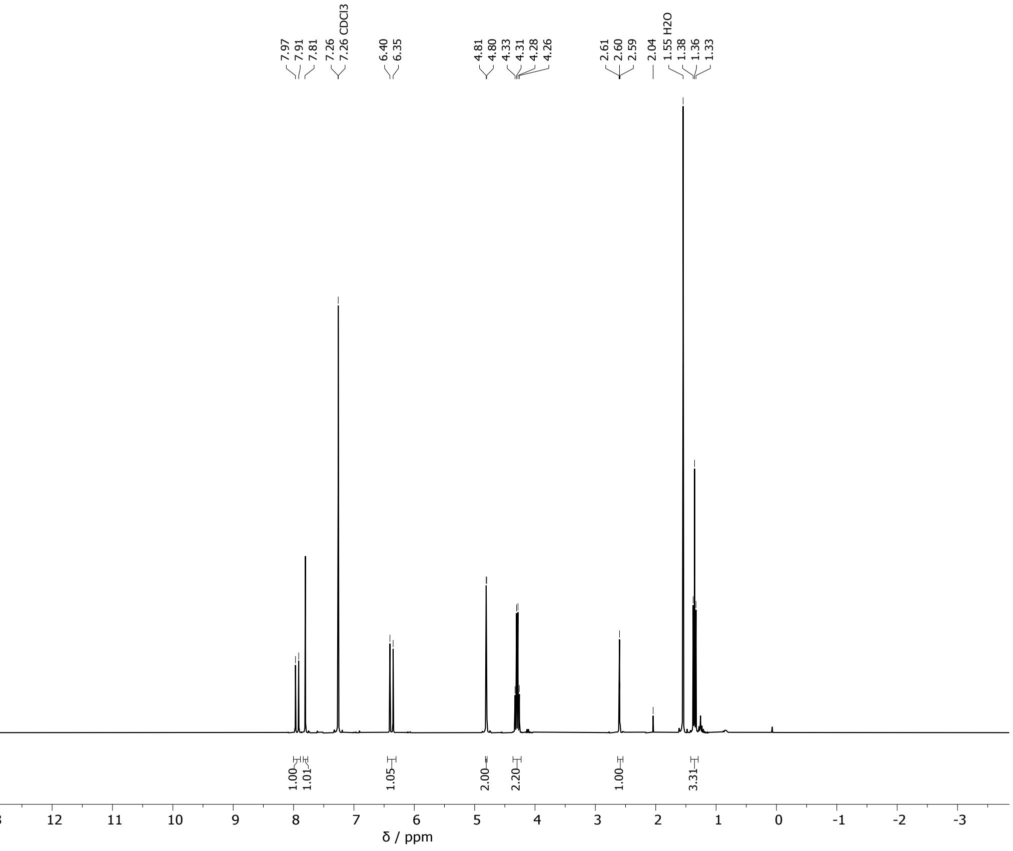

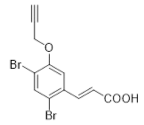

CA-2c

Chemical Formula: $C_{12}H_8Br_2O_3$
Molecular Weight: 360.00 g/mol

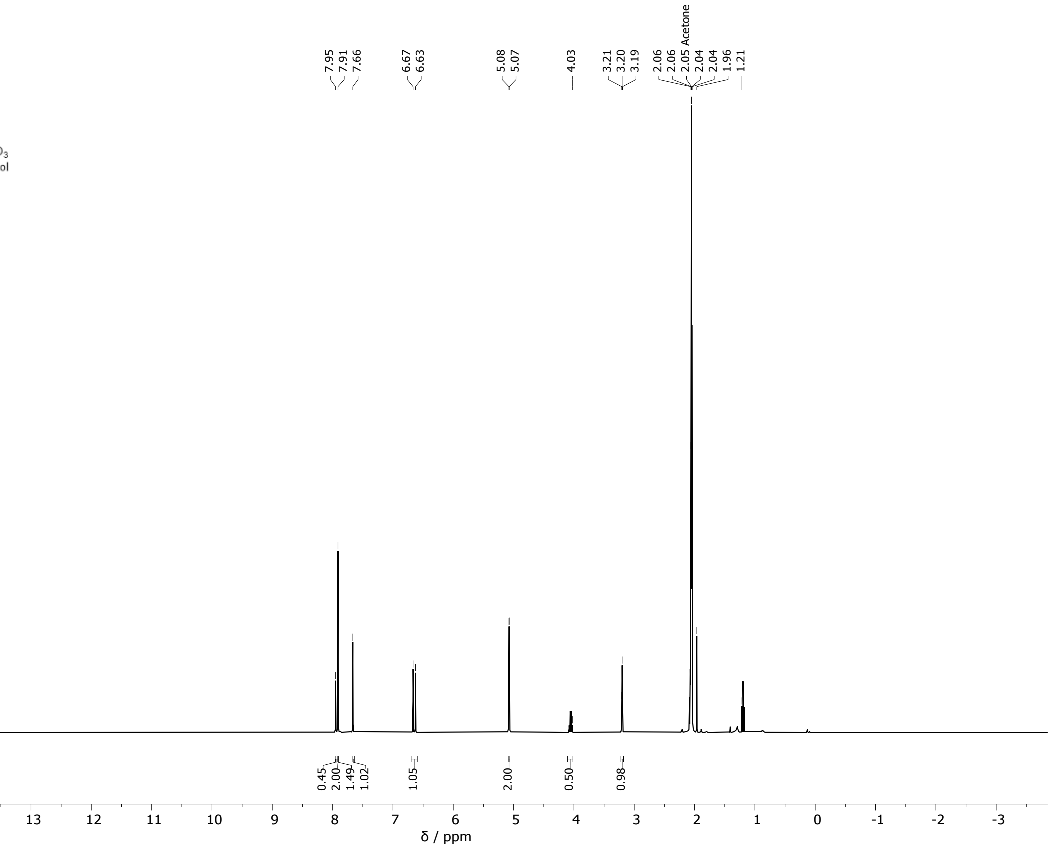

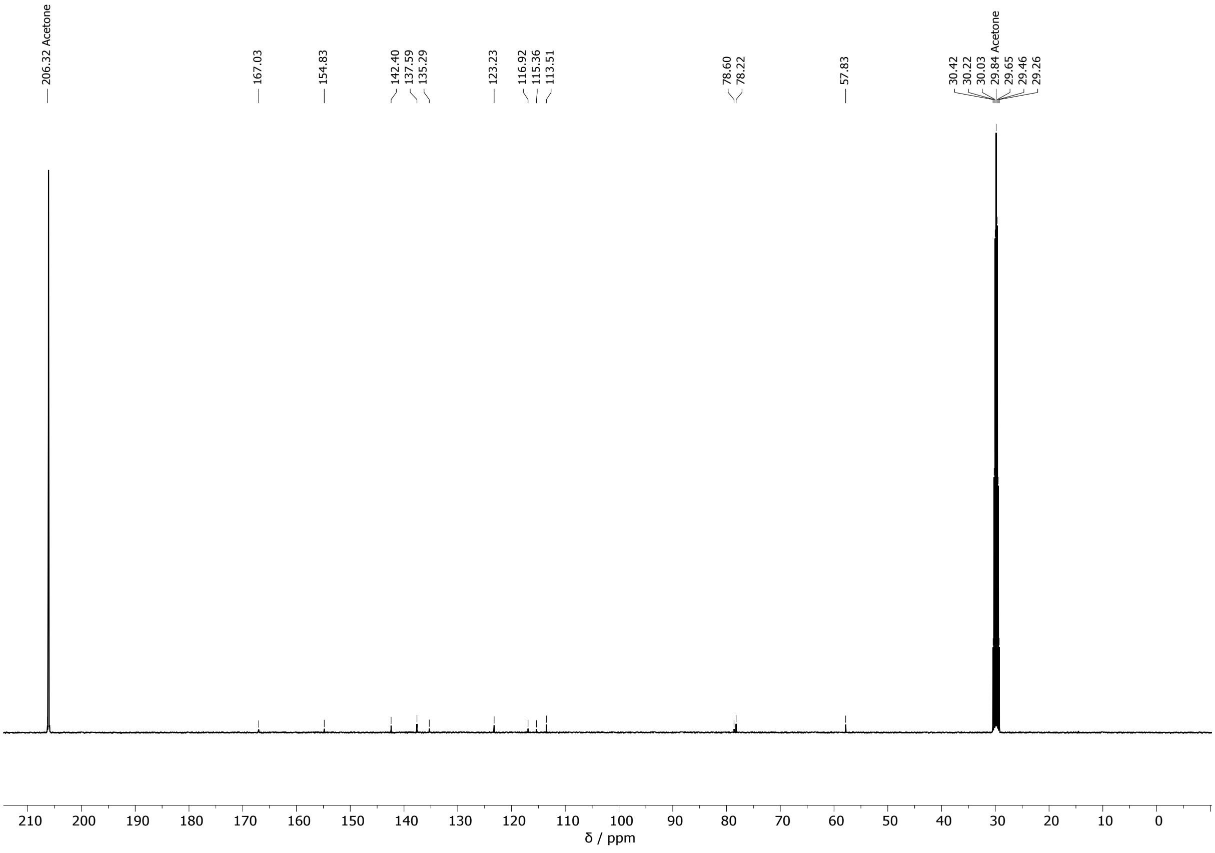

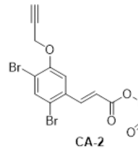

CA-2

Chemical Formula: $C_{16}H_{11}Br_2NO_5$
Molecular Weight: 457.07 g/mol

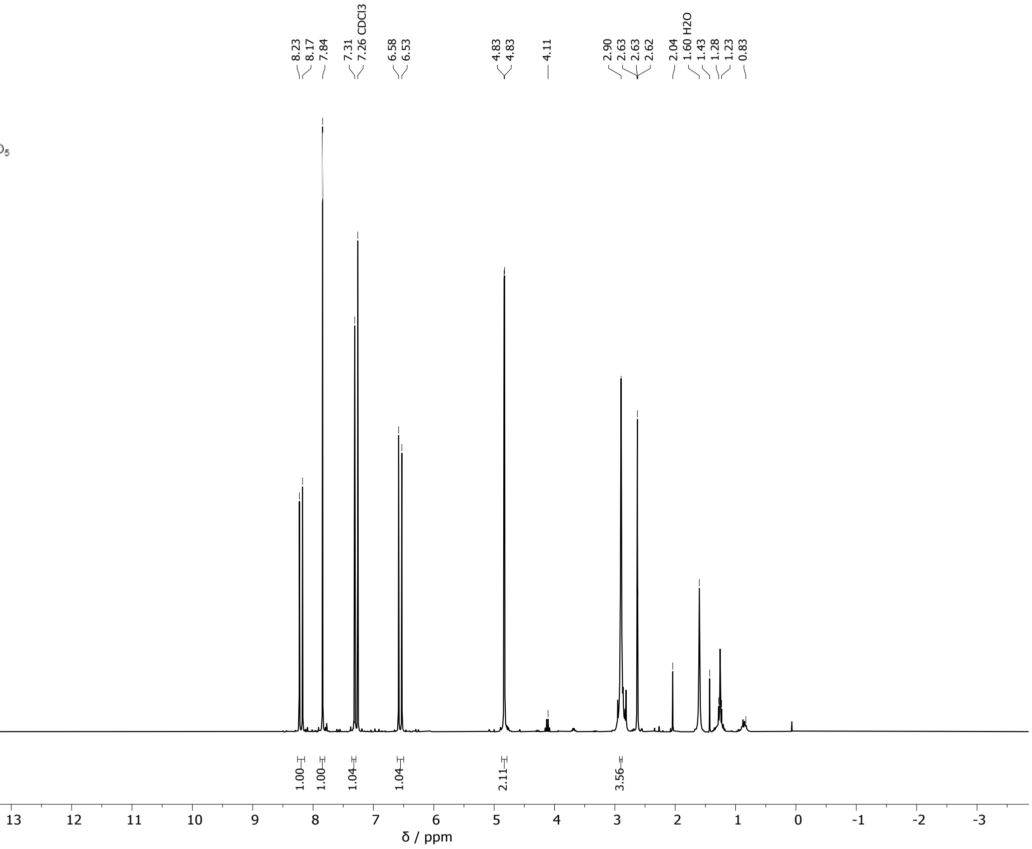

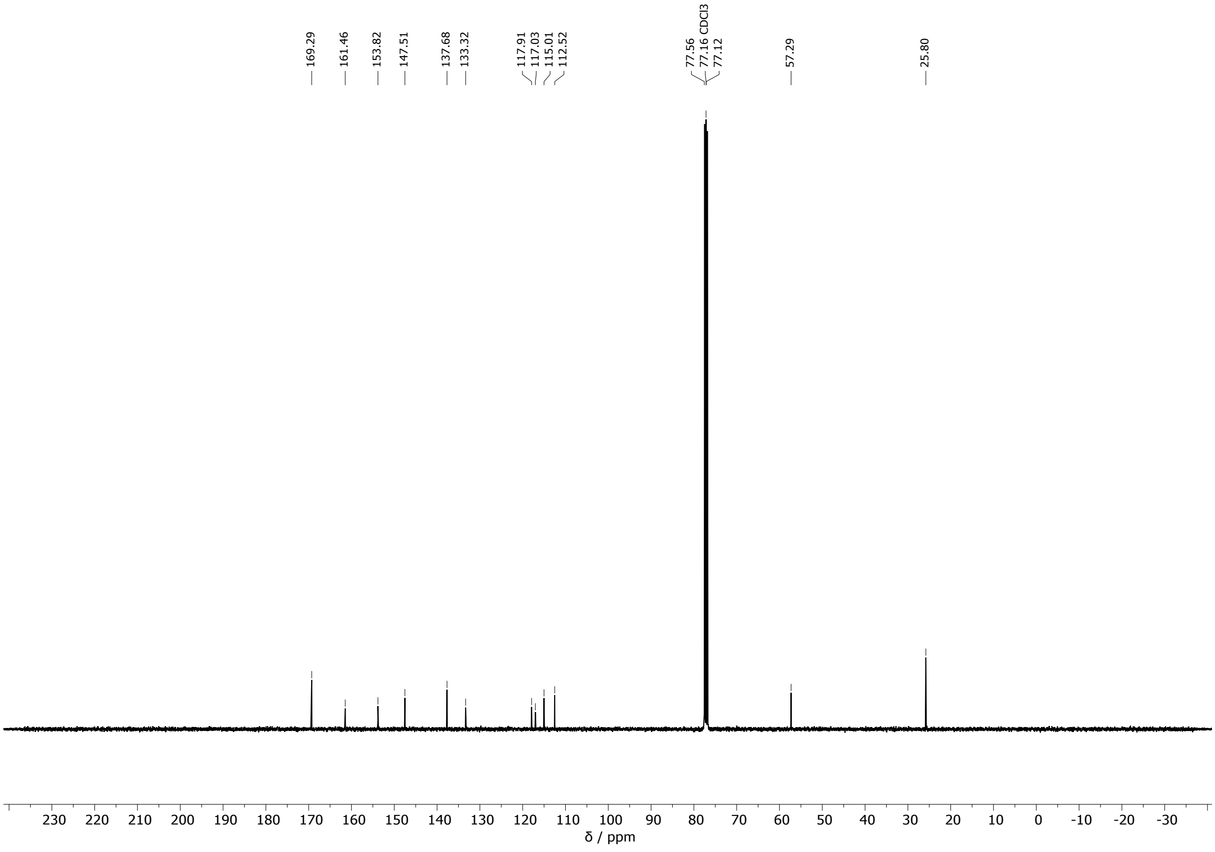

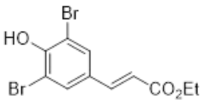

CA-3a

Chemical Formula: $C_{11}H_{10}Br_2O_3$
Molecular Weight: 350.01 g/mol

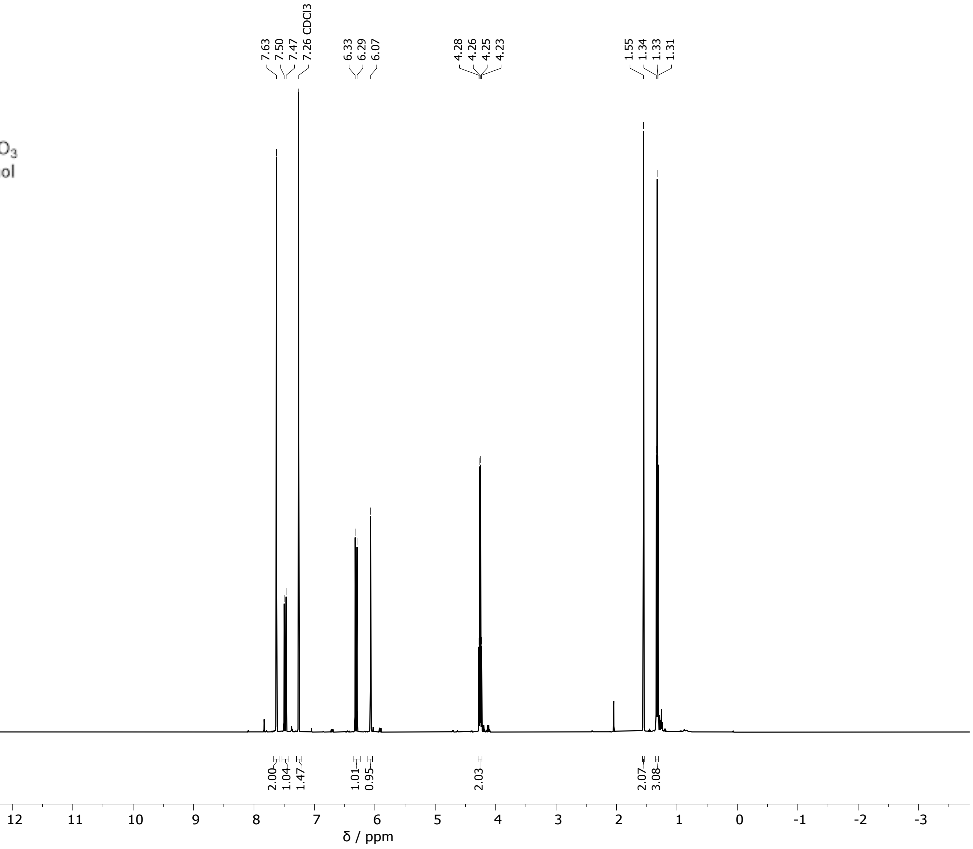

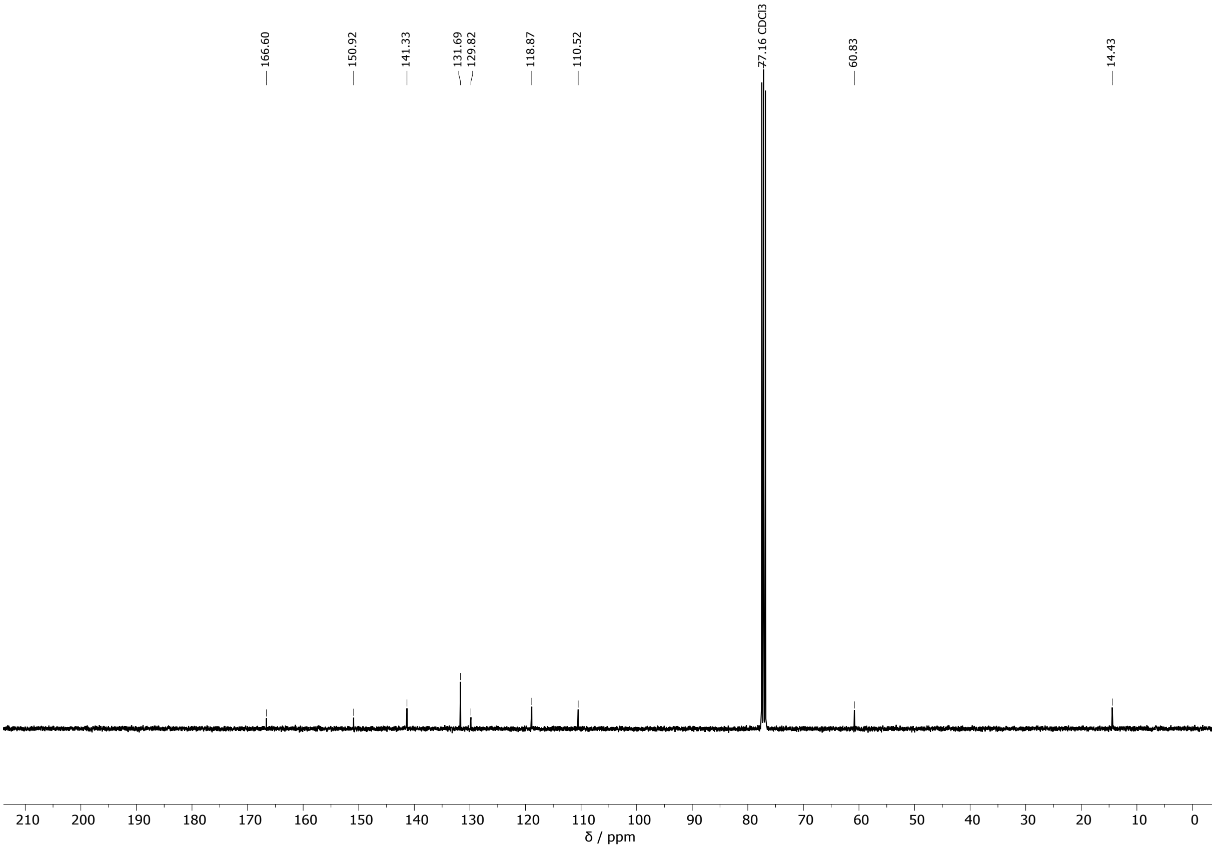

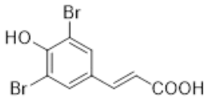

CA-3b

Chemical Formula: $C_9H_6Br_2O_3$
Molecular Weight: 321.95 g/mol

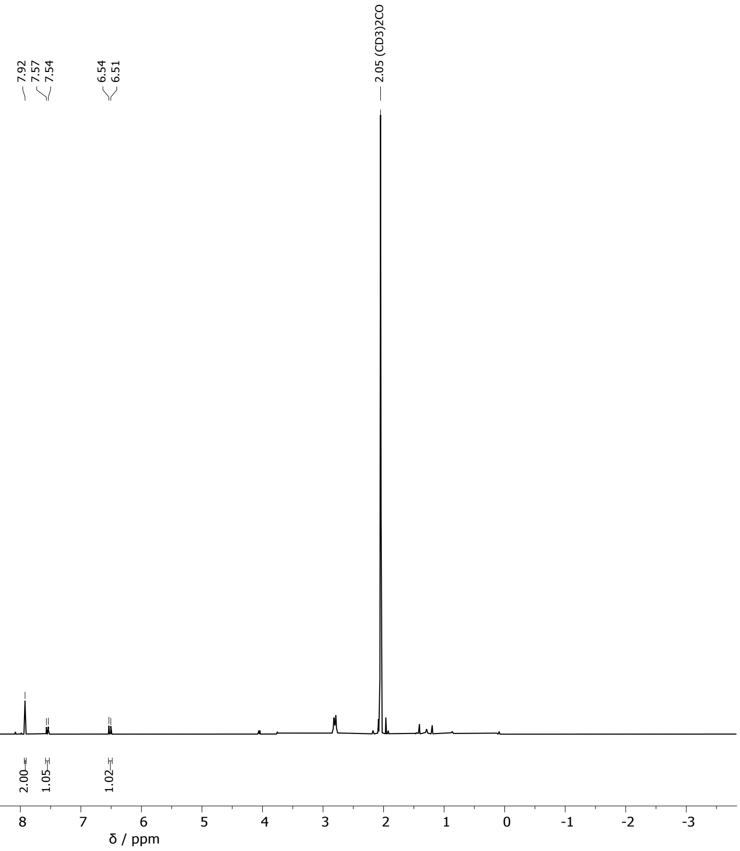

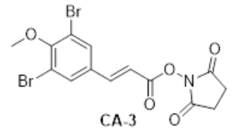

CA-3

Chemical Formula: $C_{14}H_{11}Br_2NO_5$
Molecular Weight: 433.05 g/mol

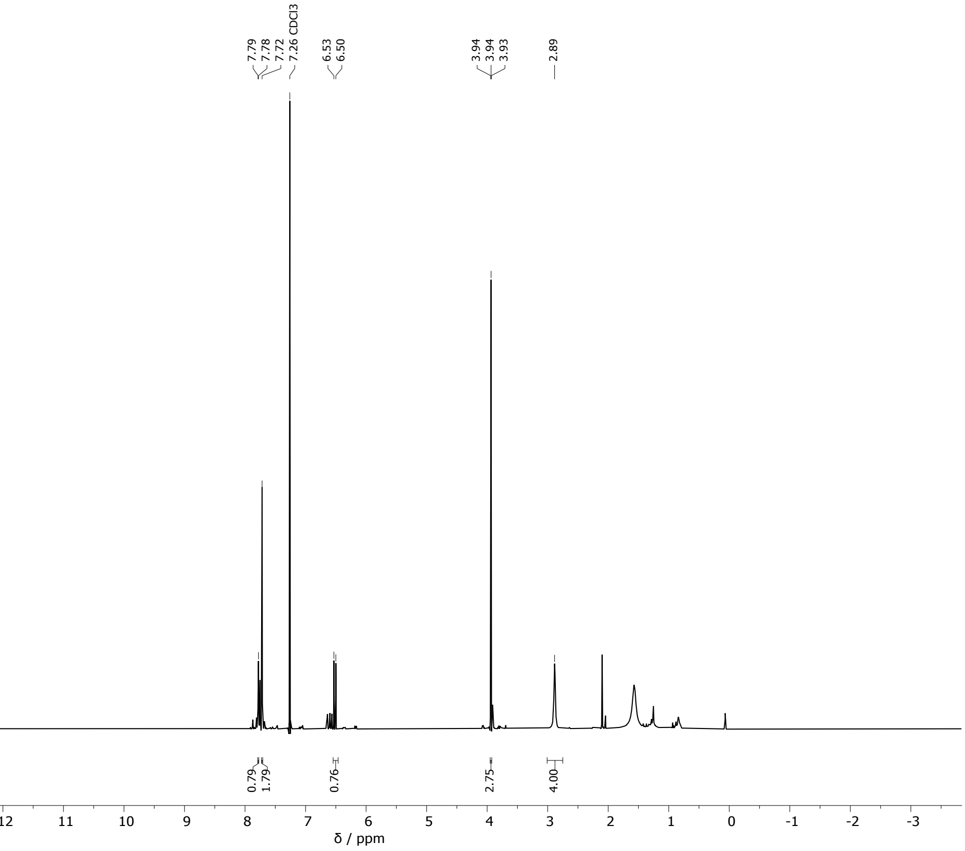

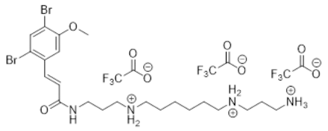

Ian-MPD-1

Chemical Formula: $C_{28}H_{38}Br_2F_9N_4O_8$
Molecular Weight: 890.43 g/mol

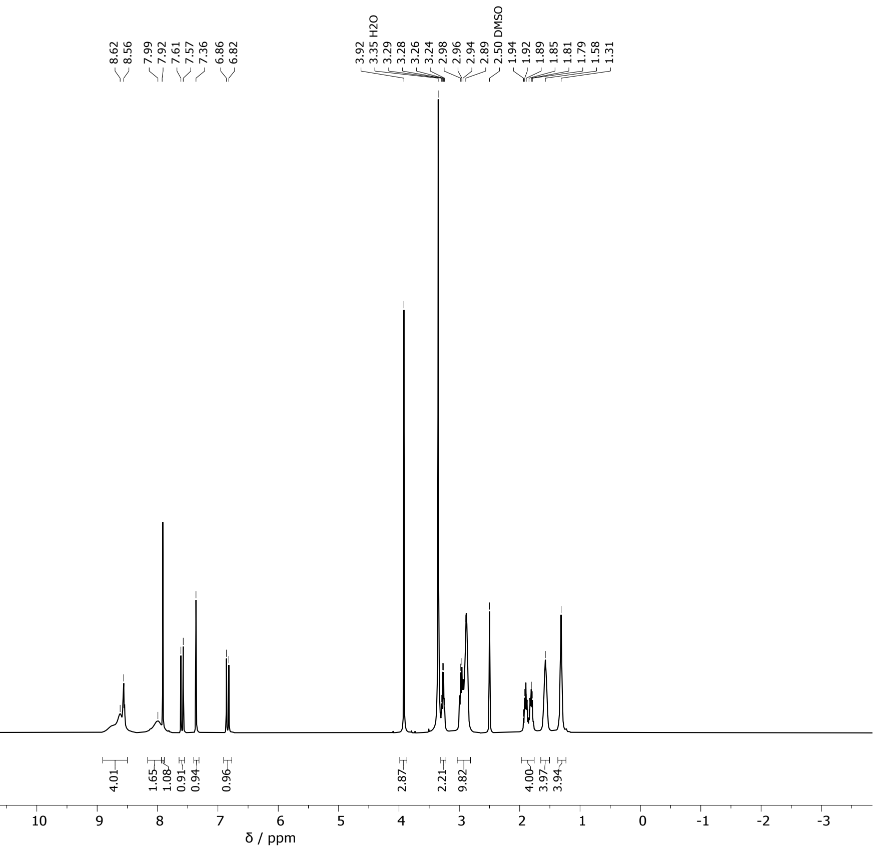

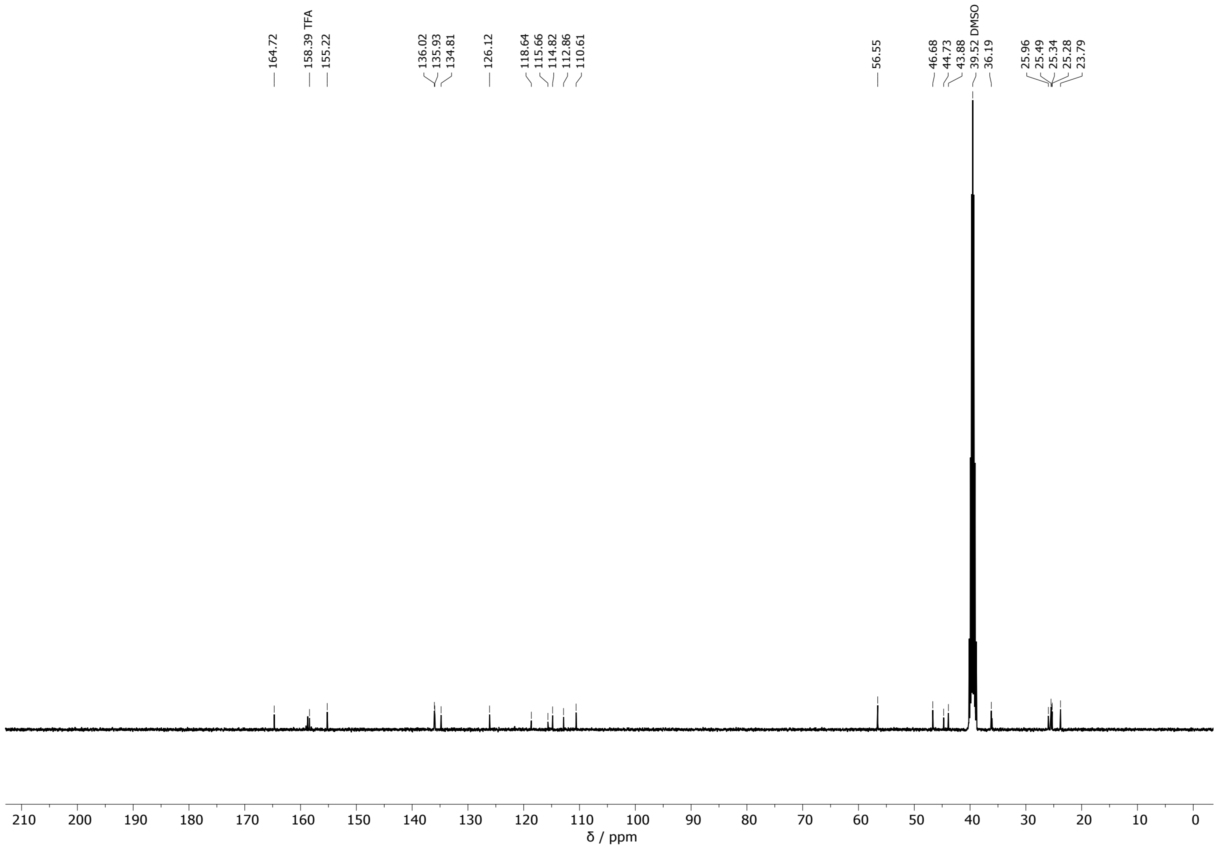

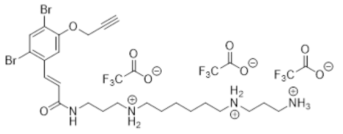

Ian-P-1

Chemical Formula: $C_{30}H_{39}Br_2F_9N_4O_8$

Molecular Weight: 914.46 g/mol

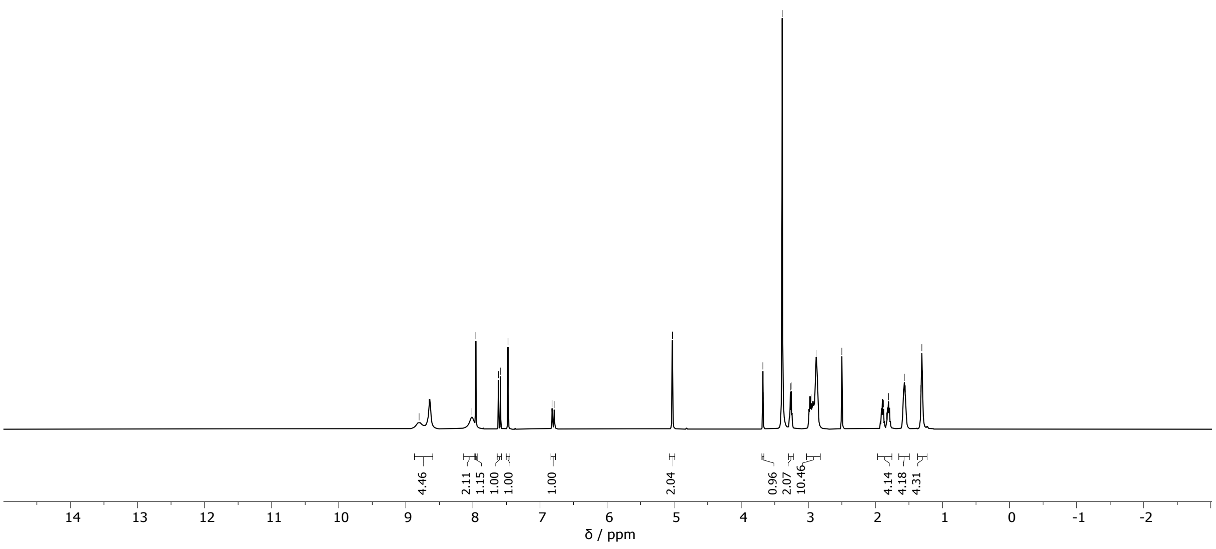

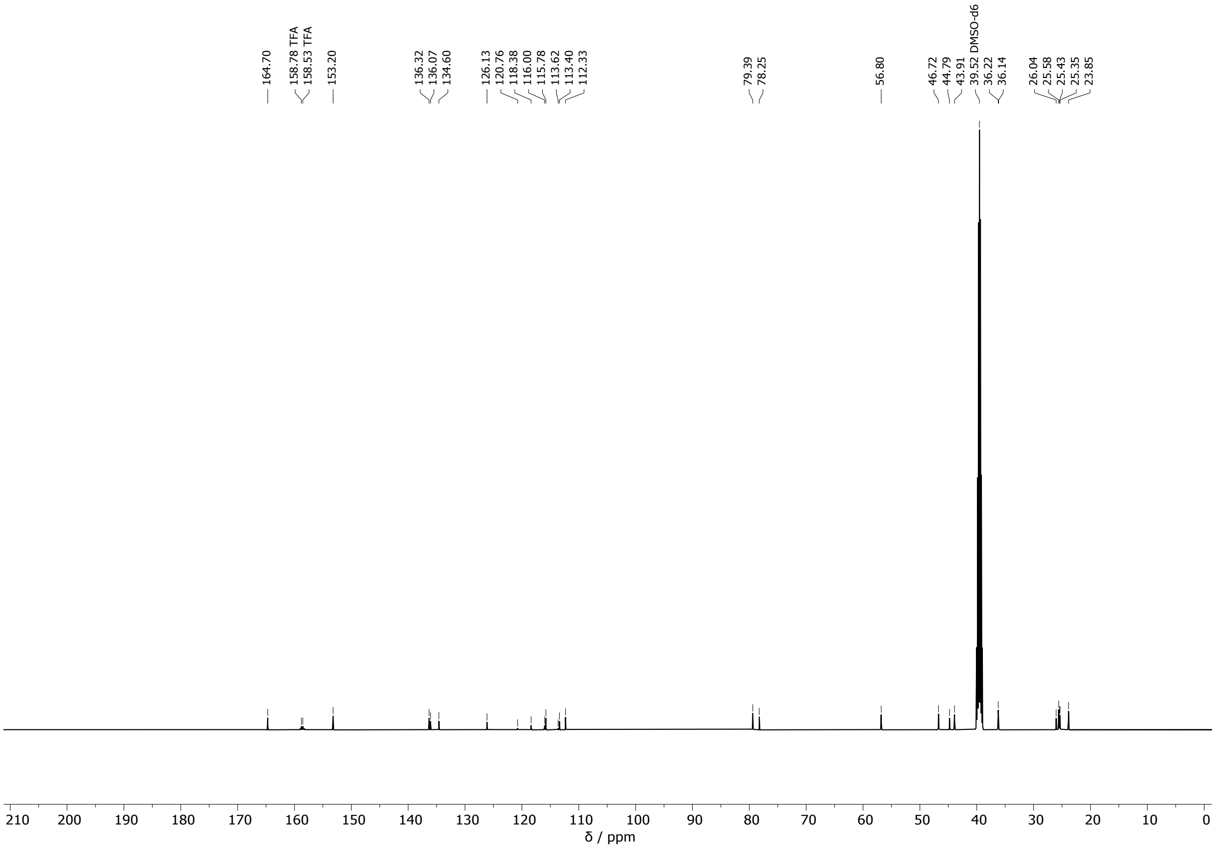

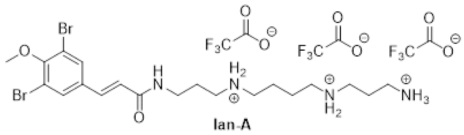

Ian-A

Chemical Formula: $C_{26}H_{35}Br_2F_9N_4O_8$
Molecular Weight: 862.38 g/mol

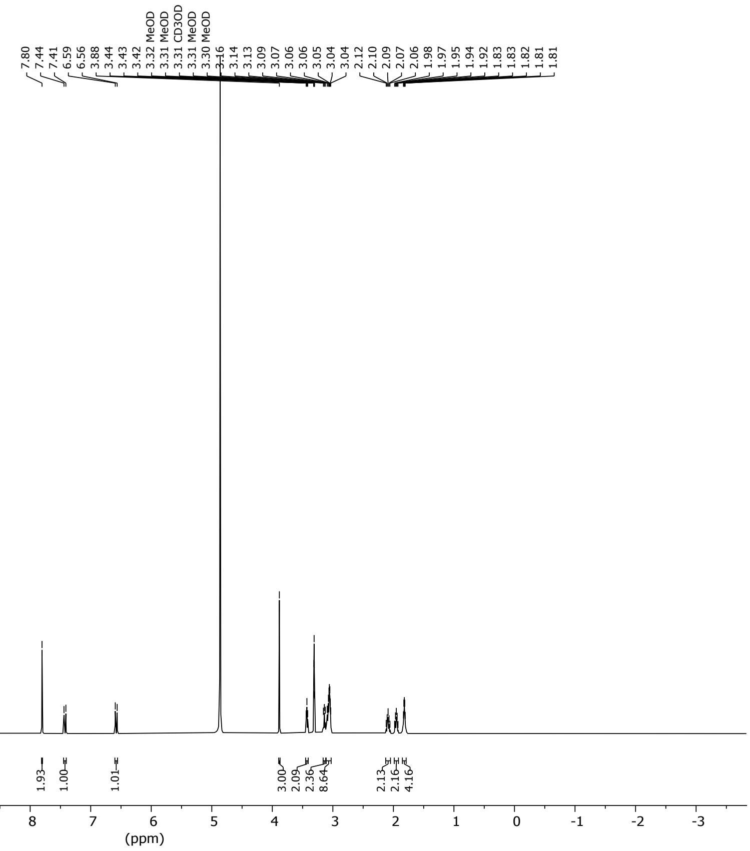

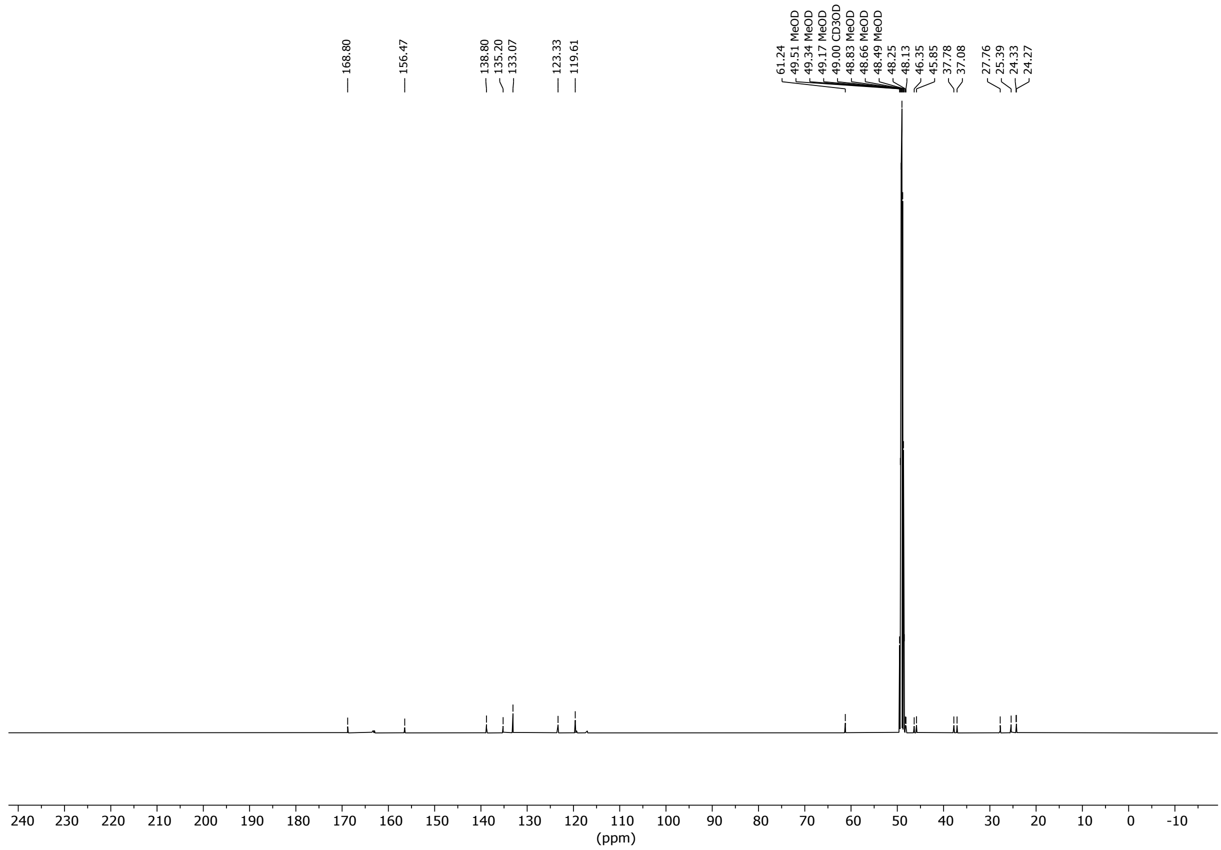

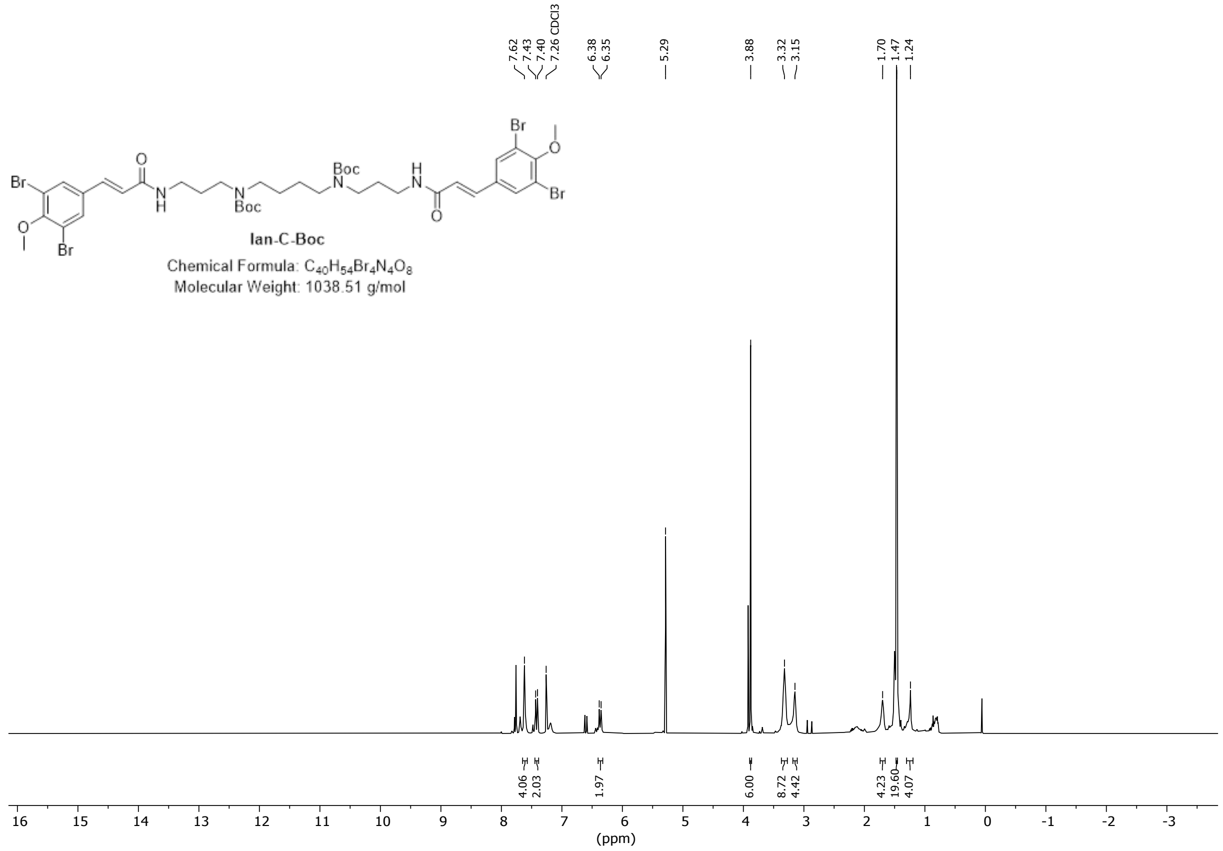
Ian-C-Boc
Chemical Formula: C40H54Br4N4O8
Molecular Weight: 1038.51 g/mol
7.62
7.43
7.40
7.26 CDCl3
6.38
6.35
5.29
3.88
3.32
3.15
1.70
1.47
1.24
4.06
2.03
1.97
6.00
8.72
4.42
4.23
19.60
4.07
(ppm)

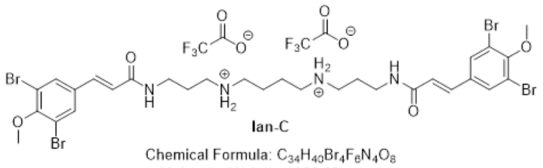

Ian-C

Chemical Formula: $C_{34}H_{40}Br_4F_6N_4O_8$
Molecular Weight: 1066.32 g/mol

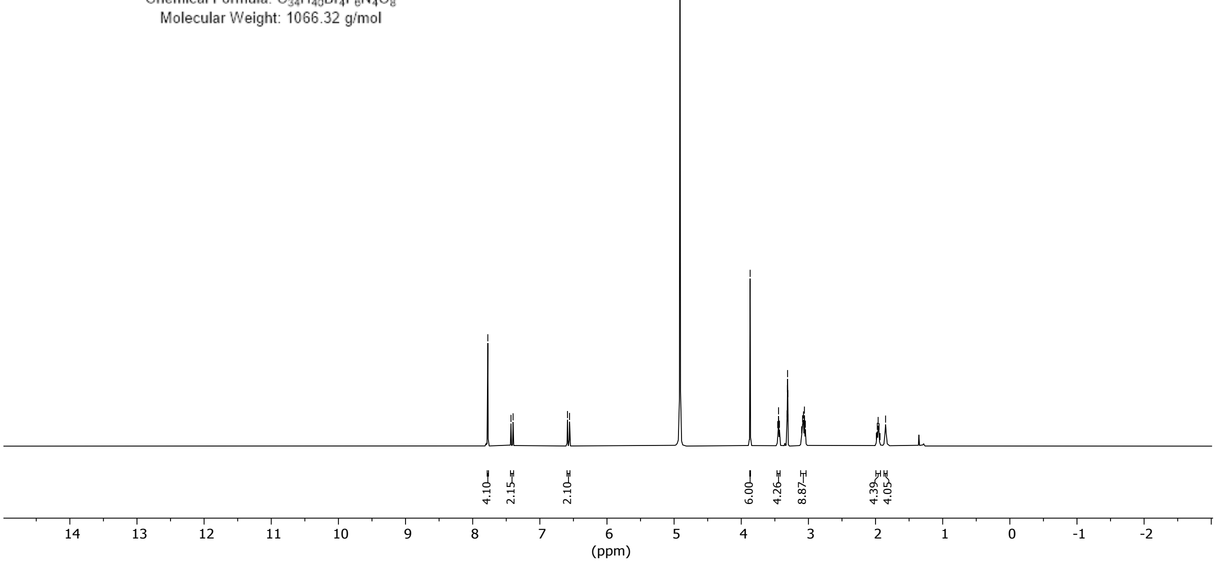

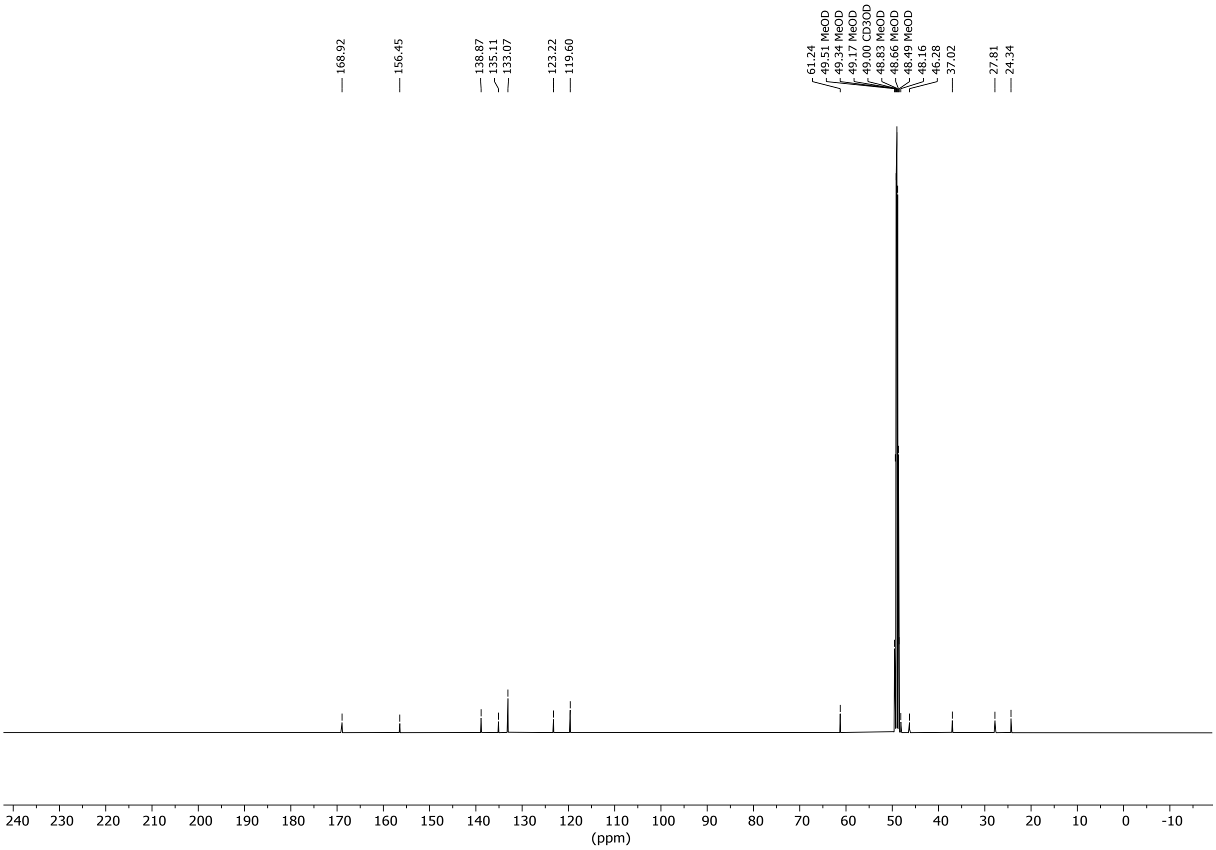

Supplement: Supplementary file 4 [file cb5c01018_si_004.pdf]
